# Highly Stable and Sensitive Fluorescent Probes (LysoProbes) for Lysosomal Labeling and Tracking

**DOI:** 10.1038/srep08576

**Published:** 2015-02-26

**Authors:** Nazmiye B. Yapici, Yue Bi, Pengfei Li, Xin Chen, Xin Yan, Srinivas Rao Mandalapu, Megan Faucett, Steffen Jockusch, Jingfang Ju, K. Michael Gibson, William J. Pavan, Lanrong Bi

**Affiliations:** 1 Department of Chemistry, Michigan Technological University, Houghton, MI 49931; 2 Second Hospital of HeBei Medical University, Shijiazhuang, China 050000; 3 Department of Chemistry, Columbia University, NY 10027; 4 Translational Research Laboratory, Stony Brook Medicine, Stony Brook, NY 11794; 5 Experimental and Systems Pharmacology, College of Pharmacy, Washington State University, Spokane WA 99202; 6 National Human Genome Research Institute, NIH, Bethesda, Maryland 20892

## Abstract

We report the design, synthesis and application of several new fluorescent probes (LysoProbes I-VI) that facilitate lysosomal pH monitoring and characterization of lysosome-dependent apoptosis. LysoProbes are superior to commercially available lysosome markers since the fluorescent signals are both stable and highly selective, and they will aid in characterization of lysosome morphology and trafficking. We predict that labeling of cancer cells and solid tumor tissues with LysoProbes will provide an important new tool for monitoring the role of lysosome trafficking in cancer invasion and metastasis.

Lysosomes play key roles in cellular metabolism, endocytosis, and the synthesis/assembly of hydrolases involved in macromolecule digestion. Lysosomes have an acidic lumen (pH 4.0–6.0) and contain various proteases which are active at acidic pH. Lysosome dysfunction has been implicated in disorders such as inflammation, cancer, neurodegenerative disease and numerous lysosomal storage diseases^1^,^2^,^3^,^4^,^5^,^6^,^7^,^8^,^9^,^10^,^11^,^12^,^13^,^14^,^15^,^16^. At present, there are two types of lysososmal markers whose function correlates with organelle acidity and the capacity for marker endocytosis. Weakly basic amines, including DAMP, neutral red, acridine orange, and LysoTracker probes can selectively accumulate in lysosomes in relation to the acidic lumen pH^17^. Nonetheless, there are still deficiencies with these compounds. For example, DAMP is not fluorescent, and an additional fluorophore, must be employed to visualize organelle staining. Additionally, neutral red and acridine orange commonly stain acidic organelles but are not specific for lysosomes. On the other hand, LysoTracker dyes are commercially available fluorescent acidotropic dyes that can specifically label lysosomes. When LysoTrackers aggregate intracellularly for longer periods, however, the cellular pH increases which may lead to quenching of the flourescent dye in addition to morphological/physiological changes of the lysosomes^17^,^18^,^19^,^20^,^21^,^22^,^23^,^24^,^25^,^26^,^27^,^28^,^29^. As mentioned above, the second category of lysosomal markers are designed based upon endocytotic properties - the ability of large molecules to enter living cells. Along these lines, dextran and bovine serum albumin (BSA) labeled with a fluorophore are often used for endosome/lysosome labeling. Nonetheless, rapid degradation and low photostablility make these biomarkers unsuitable for live cell imaging over several hours. Modified quantum dots have been explored as long-lived, photostable endosome/lysosome markers to image live cells, but continuing questions of cellular toxicity may limit the utility of these compounds^2^. Recently, Belfield et al developed a novel, two-photon-absorbing fluorene derivatives exhibiting good selectivity for lysosomes of HCT 116 colon cancer cells^26^. We describe a new approach to highly sensitive and specific fluorophore labeling of lysosomes in the current report.

## Results and Discussion

### Design rationale

**Figure 1:**
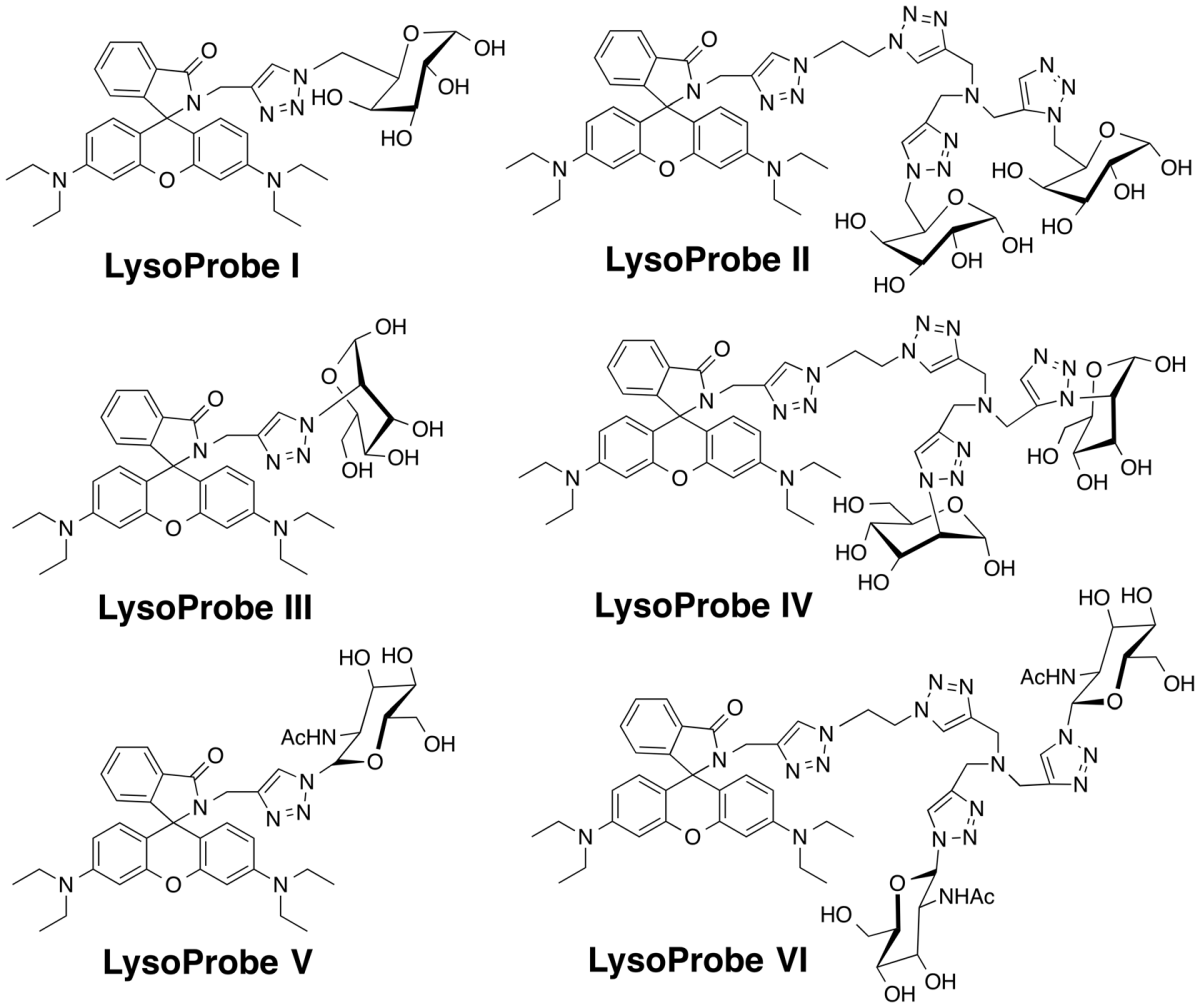
Chemical structures of LysoProbes I-VI.

We have recently developed several fluorescent probes that aggregate in lysosomes and exploited them to monitor intracellular pH and localize lysosomes in cultured cells^28^. These acidotropic probes, unfortunately, are limited in specificity since they label compartments based upon their pKa values, which are not unique for lysosomes. We have now synthesized a series of novel fluorescent probes that combine a fluorescence-responsive H^+^ domain with a lysosome-targeting moiety (chemical structure shown in Fig. 1). To design a lysosome-targeting moiety into the probes, we focused attention on lysosomal membrane proteins which are highly glycosylated and carry several N-linked glycans. The individual glycan components include mannose, N-acetylglucosamine, fucose, galactose, and sialic acid moieties, which may protect lysosomal proteins from protease degradation. Based upon the carbohydrate backbone of these glycans, we predicted that lysosome targeting could be achieved using rhodamine conjugated to N-linked glycans. Here we describe the design, synthesis, and spectroscopic characteristics of these fluorescent probes in addition to preliminary investigations into their utility in defining lysosome structure and function.

### Synthesis and structural characterization of LysoProbes I-VI

To assess the extent of potential fluorophore/N-glycan cleavage in the cellular environment, various N-linked glycans were introduced into the fluorescent probes via “click” chemistry^29^. As shown in Scheme S1, generation of LysoProbes I, III, and V were achieved using mild conditions. A “double click” methodology was employed to introduce duplicate N-linked glycan moieties into LysoProbes II, IV and VI. The spirocyclic structures of LysoProbes I-VI, rhodamine lactam-type derivatives, were confirmed by NMR. In the lactam form, spirocyclics lack measurable absorbance and fluorescence in the visible spectrum, which is restored when they are converted to the corresponding amides. The spirocyclic structure of LysoProbes I-VI was further confirmed using UV-Vis titration. A shift from basic to acidic pH extinguishes the absorbance of LysoProbes. The absorption and fluorescence emission spectra of LysoProbes I-VI are highly pH-dependent, with maximal absorption at 563 ± 4 nm under acidic conditions (Fig. S10).

LysoProbes I-VI yield highly fluorescent, pink chromophores under acidic conditions, readily facilitating visual identification of the pH environment. The fluorescence maximum of LysoProbe **I** was 583 ± 4 nm (Fig. S10) with minimal intensity beyond pH 7.2; as the pH decreased from 8.4 to 4.6, the fluorescence intensity increased 215-fold. Similarly, the fluorescence intensity of LysoProbe II increased 315-fold as pH decreased from 8.4 to 4.9. These probes maintained strong fluorescence at low pH and constant concentration, making them superior to other probes (e.g., fluorescein) whose fluorescence is significantly quenched under acidic conditions.

### Intracellular localization of LysoProbes I-VI

**Figure 2:**
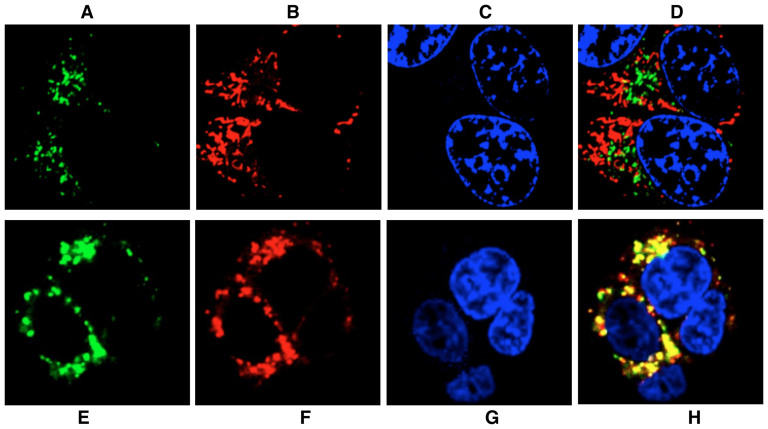
Intracellular distribution of LysoProbe II compared to MitoTracker and LysoTracker Green. LysoProbe **II** (30 μM, B) was incubated with cells in DMEM media without FBS and counterstained with MitoTracker (80 nM, A), Hoechst 33342 (1 μg/mL, C); and overlay (D); (E–H): HeLa cells incubated with LysoProbe **II** (10 μM, E), followed by counterstaining with LysoTracker (2 μM, F), Hoechst 33342 (1 μg/mL, G); and overlay (H); Cells were imaged on an inverted laser scanning fluorescent microscope (Olympus) using a 60 × oil immersion objective lens.

Cancer cells produce the bulk of their energy requirement via glycolysis, with concomitant lactic acid production. Increased acidity depletes oxygen content in tissues. Malignant cells thrive in this oxygen poor, acidic environment with enhanced resistance to therapeutic interventions. Accordingly, we examined the capacity of LysoProbes to monitor intracellular pH alterations in HepG2 cells (human hepatocellular liver carcinoma cell line) and HeLa cells (human cervical cancer cells). Experiments were performed in non-fixed, growing cells to prevent cell-fixation artifacts. Following 15-min incubation with LysoProbes I-VI, there was a clearly visible intracellular localization (Fig. 2 and Fig. S1). Repetitive washing and incubation of probe-loaded cells (30 min, LysoProbes I-VI) in dye-free medium revealed high intracellular fluorescent signal over a 24-hour period. Studies with increasing probe concentrations revealed no evidence of cell damage associated with probe internalization, and bright-field examination revealed cellular viability at the highest concentration (30 μM) of LysoProbes I-VI.

**Figure 3:**
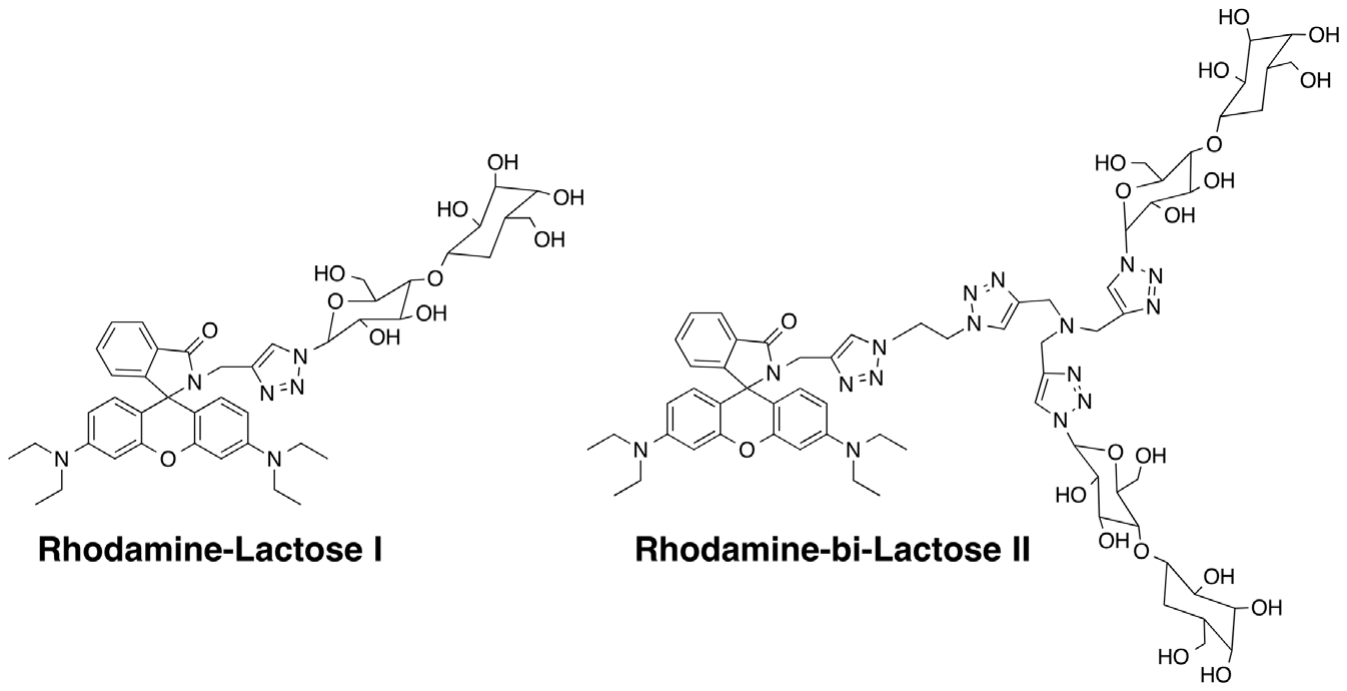
Chemical structures of rhodamine-lactose conjugates (I and II) without glycan moieties.

The cellular localization of fluorescent probes was examined using distinct organalle markers. Cellular probe distribution suggested localization in either acidic vesicles or mitochondria following 45 min incubation with probes I-VI. Subsequently, a double stain with MitoTracker and LysoProbes I-VI was undertaken in HeLa cells. This resulted in a distinct labelling pattern that was non-mitochondrial in pattern (A–D, Fig. 2 and Fig. S1). These studies were followed by double staining with LysoTracker Green (a lysosome selective stain), indicating localization of fluorescent probes I-VI in lysosomes (45 min., E–H, Fig. 2 and Fig. S1). Nuclear staining (Hoechst 33342) verified cellular viability throughout the imaging experiments. Consistent with our prediction that N-linked glycan moieties of LysoProbes would facilitate lysosomal localization, we observed a punctuate fluorescence pattern indicative of lysosomal targeting. To further confirm the necessity of N-glycan presence for lysosomal targeting, we prepared two control compounds lacking N-glycans, rhodamine-lactose I and rhodamine-bi-lactose II (chemical structure shown in Fig. 3). Subsequent studies verified that these compounds did not target to lysosomes (Fig. S2 & S3), lending further support to our hypothesis that N-linked glycans on LysoProbe I-VI facilitate specific lysosomal accumulation and retention.

### Further characterization of the lysosome-targeting moiety

**Figure 4:**
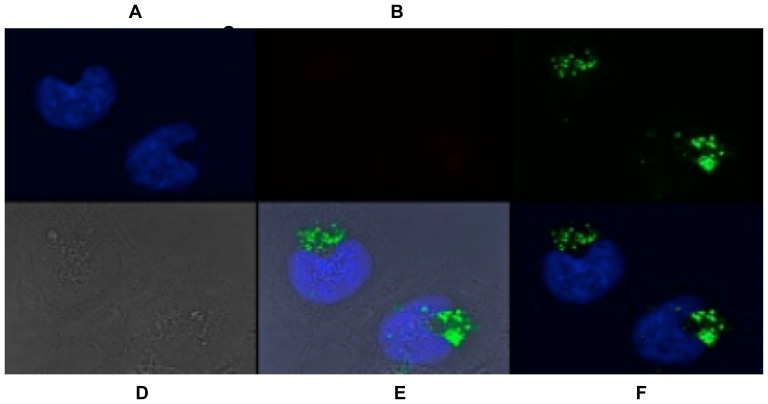
Confocal laser-scanning fluorescent images of LysoProbe I in HeLa cells. LysoProbe I (2 μM, green, C) was incubated with cells in non-FBS DMEM media for 15 min., and then counterstained with LysoTracker (2 μM, no fluorescence, B), Hoechst 33342 (1 μg/mL, blue, A); DIC image (D), overlay (A+B+C) with DIC (E), overlay (A+B+C) without DIC image (F). All images were acquired using a 60 × objective lens following 24 h incubation.

For compounds 2 & 5 (Scheme S1; LysoProbe I-VI precursors), fluorescence of HeLa cells following probe loading quickly dissipated, likely due to rapid leakage, thereby reducing sensitivity. Conversely, LysoProbe I-VI (containing N-linked glycans) was well-retained in living cells for long periods of post-probe loading (Fig. 4 and Fig. S4–S8). This result suggested that the presence of N-glycans enhances intracellular retention, although this retention is likely to vary between different cell lines. We further examined alterations of fluorescence properties induced by the inclusion of N-linked glycans. The pKa values of LysoProbes containing N-glycans were comparable to their synthetic precursors. Moreover, the inclusion of up to two N-linked glycan moieties enabled LysoProbes to be well-retained in living cells without a corresponding decrease of fluorescence intensity (Table S1).

### Intracellular pH evaluations

**Figure 5:**
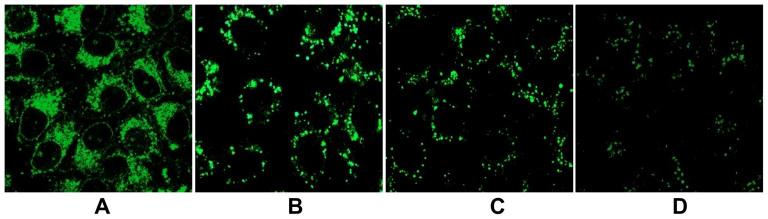
Characterization of intracellular pH using LysoProbe II (10 μM). After washing three times with corresponding pH buffers, the cells pre-loaded with LysoProbe II were incubated with nigericin and subsequently imaged in pH 4.4 (A), pH 5.0 (B), pH 5.5 (C) and pH 6.5 (D) buffers. The cells were imaged for fluorescence on an inverted laser scanning fluorescent microscope (Olympus) using a 60 × oil immersion objective lens.

We next examined intracellular alterations of pH in LysoProbe-loaded cells. These studies were facilitated in cells treated with the H^+^/K^+^ antiporter nigericin, followed by fluorescent imaging of LysoProbe-loaded cells at various pHs. The intracellular pH responsiveness of LysoProbe **II** was inversely related to pH (Fig. 5). In all experiments, the intracellular pH was maintained in equilibrium with the extracellular pH. Additionally, we noticed that HeLa cells maintained at an acidic extracellular pH exhibited altered lysosomal morphology (Fig. 5), in which lysosomes localized to the perinuclear region. Conversely, the lysosomes of the cells maintained at pH 6.5 (Fig. 5D) were more dispersed and located nearer to the periphery of cells.

### Lighting up cancer cells

**Figure 6:**
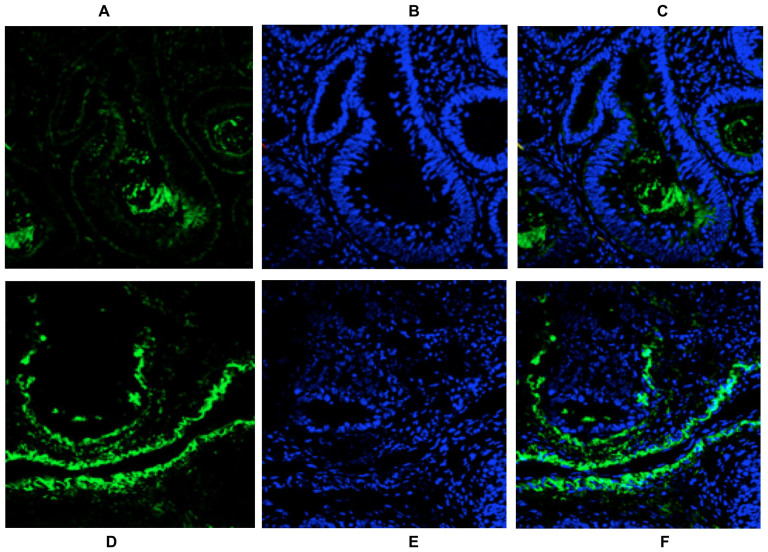
Confocal laser-scanning fluorescence imaging of freshly frozen colon tumor tissue-slices from a patient following incubation of LysoProbe I/II (20 μM). LysoProbe fluorescence images are displayed in green (A, D), with nuclei counterstained by Hoechst 33342 and displayed in blue (B, E), and overlay images (C, F). Tissues were imaged on an inverted laser scanning fluorescent microscope (Olympus) using a 60 × oil immersion objective lens.

Enhanced glycosis in cancer cells results in an up-regulation of the galactose transporter^30^. Taking advantage of this change, we hypothesized that LysoProbes might serve as substrates in cancer cells that could donate galactose residues required for the synthesis of glycosylated proteins. In line with this hypothesis, we predicted that LysoProbes should manifest selective accumulation in tumors. To examine our hypothesis, we undertook fluorescence microscopy of fresh tumor slices pre-loaded with our LysoProbe (Fig. 6). The fluorescence intensity of this LysoProbe was more prominent in selected tumor areas, although it was present in varying degrees in all tumor regions. We believe this finding reflects accumulation of this LysoProbe in more aggressive tumor areas in which increased protein glycosylation is occurring. Nonetheless, these initial findings suggest that selected LysoProbes may be a useful adjuvant for tumor identification, especially in the early stages of development. Moreover, although further analyses are in order, selected LysoProbes should aid in the differentiation of more aggressive tumors in correlation with increased fluorescence intensity.

### Characterization of lysosome-dependent apoptosis using LysoProbes

Lysosomes may be involved in apoptotic signaling pathways via release of capthesins. The pairing of autophagy inhibition with caloric restriction has recently emerged as a novel treatment paradigm in cancer therapeutics. With this in mind, we hypothesized that chloroquine (CQ), an antimalarial drug recently shown to induce apoptosis in cancer cells^30^, would sensitize the human cholangiocarcinoma (CCA) cell line RBE to nutrient deprivation. Our rationale for this hypothesis centers on the observation that cancer cells are hypersensitized to nutrient deprivation due to an increased reliance on glucose oxidation. To test our hypothesis, we monitored the lysosomal changes using LysoProbe in intact cells following combinatorial treatment with CQ and nutrient deprivation.

#### Treatment of RBE cells with CQ leads to lysosome swelling

**Figure 7:**
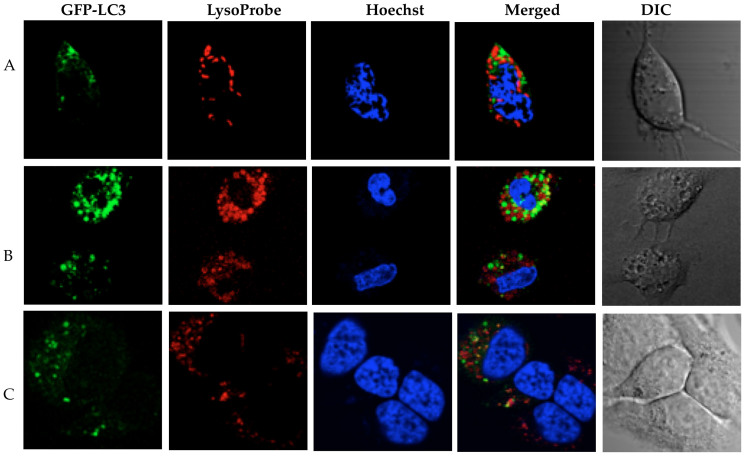
RBE cells transfected with GFP-LC3 and subsequently loaded with LysoProbe II (10 μM). RBE cells treated with CQ in the presence of standard serum: (A) CQ (20 μM) for 1 h; (B) CQ (20 μM) for 24 h; (C) CQ (20 μM) + BafA1 (10 nM) for 24 h. Cells were imaged on an inverted laser scanning fluorescent microscope (Olympus) using a 60 × oil immersion objective lens.

To measure autophagy following CQ treatment, we transfected RBE cells with the autophagosome marker LC3. To estimate autophagolysosome formation, transfected RBE cells that had been transfected with GFP-LC3 which were loaded with LysoProbe. Vacuole formation was observed following 1 h of CQ treatment (media with standard sera; Fig. 7A). Lysosomes became swollen after 24 h of CQ treatment (Fig. 7B). Since CQ is a weak base, we predicted that it might preferentially accumulate in the acidic lysosome, and we hypothesized that lysosomal acidification would block CQ toxicity. Accordingly, bafilomycin A1 was employed to inhibit the vacuolar H+-ATPase (V-ATPase) and prevent the formation of autophagolysosomes. Our data revealed that bafilomycin A1 inhibited vacuole formation, lysosomal swelling and cell death in CQ-treated RBE cells (Fig. 7C). We speculate that protonation/deprotonation of CQ is critical for its accumulation in lysosomes. Thus, in the protonated (uncharged) form CQ can freely traverse the lysosome membrane, but once protonated (charged) it is trapped within the organelle, inducing expansion of lysosomal volume and inhibition of key proteolytic enzymes.

#### Autophagosomes and swollen lysosomes fail to fuse in RBE cells undergoing combinatorial CQ/nutrient deprivation treatment

**Figure 8:**
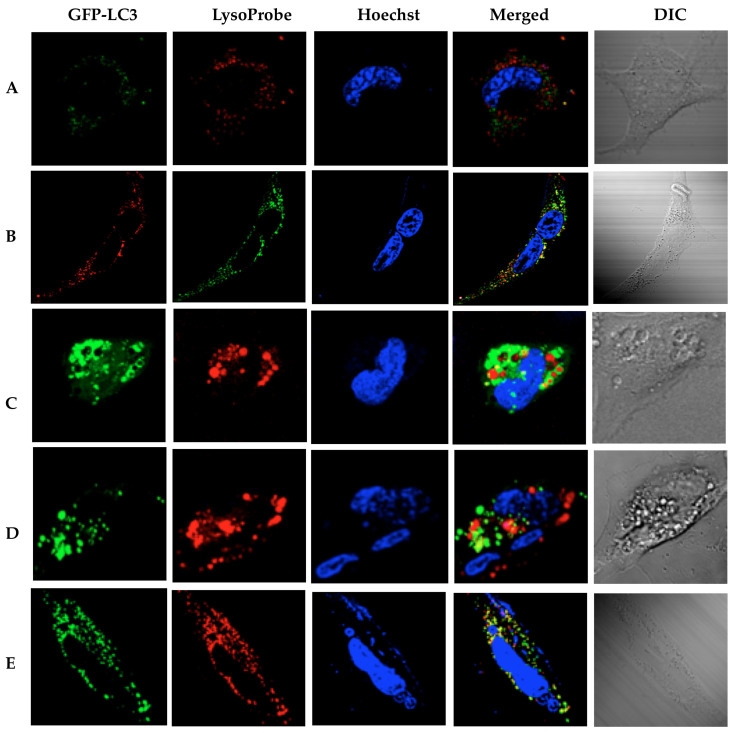
Autophagosomes and swollen lysosomes fail to fuse in CQ-treated RBE cells (loaded with LysoProbe II, 10 μM): (A) the control cells in medium with standard serum, and (B) after nutrient (serum) deprivation; (C) after CQ treatment (40 μM, 6 h) in the absence of standard serum; (D) after CQ treatment (40 μM, 16 h) in the absence of standard serum; (E) as positive control, RBE cells expressing GFP-LC3 were treated with rapamycin (1 μM, 16 h) in the absence of standard serum.Cells were imaged on an inverted laser scanning fluorescent microscope (Olympus) using a 60 × oil immersion objective lens.

Serum-deprived RBE cells, in the absence of any additional treatment, manifested a prominent overlap of GFP-LC3 and lysosome signals (Fig. 8B), suggesting that autophagy and lysosomes are both active. Following exposure to CQ (40 μM; 1 h), serum-deprived cells revealed the presence of ring-shaped autophagic vesicles (AVs) that were likely autophagosomes; these increased significantly after 6 h of CQ treatment. Moreover, 16 h exposure to CQ (40 μM; serum deprivation) revealed cells with a complete separation of GFP-LC3 and lysosome signals (Fig. 8C & D), indicative of decreased autophagolysosome formation. To further confirm this, the serum-deprived RBE cells expressing GFP-LC3 were treated with an inducer of autophagy, rapamycin (1 μM). Rapamycin intervention led to significantly increased levels of punctuate GFP-LC3 staining, while simultaneously enhancing the overlap of GFP-LC3/lysosome signals. These findings indicated functional autophagy.

**Figure 9:**
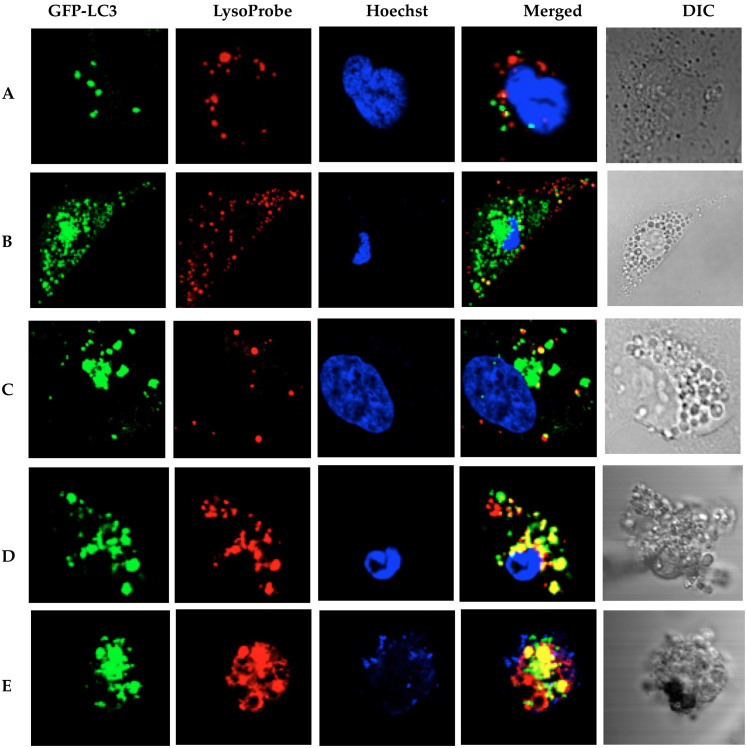
Visualization of lysosomal alterations using LysoProbe II in RBE cells undergoing apoptosis: (A–C): cells treated with CQ in standard serum for 16 h: (A) CQ (50 μM); (B) CQ (100 μM); (C) CQ (200 μM); (D) CQ (300 μM); (E): after treatment with CQ (50 μM), in the absence of standard serum, for 16 h. Cells were imaged on an inverted laser scanning fluorescent microscope (Olympus) using a 60 × oil immersion objective lens.

We further found that the absolute magnitude of GFP-LC3 punctate staining was also quite dependent upon CQ concentration (Fig. 9). This increase may have correlated with an inhibition of proper lysosome function due to the induction of autophagy and/or the corresponding formation of autophagolysosomes. To further investigate the etiology of increased GFP-LC3 punctate staining due to CQ, we employed standard assays to examine the levels of specific autophagy substrates, including p62/SQSTM1^30^.

Based upon LysoProbe fluorescence, our data indicated that serum deprivation alone resulted in a slight increase of acidic vesicles. Moreover, the number of acidic vesicles increased with CQ intervention in a concentration-dependent manner (Fig. 9). In nutrient-rich medium (complete sera), lysosome numbers were sparse throughout the cytoplasm of control cells (image not shown). Treatment with CQ at low concentration (50 μM) in nutrient-rich medium resulted in increased lysosome numbers focused around the cell nucleus (Fig. 9A). Increasing the CQ concentration resulted in movement of lysosomes toward the peripheral boundaries of the cells (Fig. 9B). Still higher CQ (>200 μM) led to obvious morphological differences of RBE cells, characterized by a rounded appearance and noticeable aggregation (Fig. 9C &D). On the other hand, nutrient deprivation resulted in the development of a compact lysosome mass at the center of the cell, even at lower CQ concentration (50 μM), which suggests an impairment of lysosomal membrane stability (Fig. 9E). Taken together, our data indicated that CQ (even at concentrations > 300 μM) induced only a moderate and transient vacuolization of cells cultured in nutrient-rich medium. However, conditions of nutrient depletion, combined with much lower CQ concentrations, proved effective in cancer cell death. These data indicate that nutrient deprivation increases the sensitivity of cancer cells to CQ-induced lysosomal swelling and ensuing cell death.

In samples from the RBE cells not transfected with LC3, there was an increase in LC3-II 1 h after treatment with CQ, which continued to rise as time passed, shown by western blots with anti-LC3 antibody. To examine whether protein degradation via autophagy was activated in CQ-treated cells, we performed immunoblotting with the anti-p62 antibody. Interacting with LC3-II and ubiquitin protein, the p62 protein degraded during autophagy, whereas an increase of p62 indicates inhibited autophagic degradation. After 16 h exposure of CQ, the p62 level elevated, implying autophagic degradation was inhibited. Combined serum deprivation and CQ treatment showed increased levels of autophagosome associated LC3-II, which suggests the initiation of autophagy and deficiency of proteolysis of LC3-II by CQ. P62 protein expression level was reduced during serum depletion but noticeably increased with CQ treatment regardless of serum concentration which confirms its ability to block autophagic proteolysis. (Fig. S9). Cumulatively, our data also demonstrates that nutrient deprivation primes human RBE cancer cells for the synergistic effects of CQ treatment. Potential CQ toxicity can be reduced by using drug concentrations much lower than those currently employed in the clinic, accordingly, we predict that combined treatment with chloroquine and caloric deprivation holds considerable promise in cancer therapy.

## Conclusions

Several features (absence of cytotoxicity, high cellular permeability, long-lived intracellular fluorescence, selective accumulation within acidic organelles) of our newly synthesized probes identify them as superior candidates for examining acidic intracellular pH alterations and intracellular labeling of acidic organelles (lysosomes). Unlike commercially available lysosomal markers, we did not observe a decrease in fluorescent signal when preloaded cells were incubated in dye-free medium, suggesting excellent intracellular retention. As well, our fluorescent probes demonstrated excellent stability, even in dye-free incubation medium, over long periods of time. This feature enables broad experimental opportunities for live imaging over extended time periods in dye-free medium without the confound of fluorescent signal loss. Thus, our probes provide a novel and functional set of tools to evaluate lysosomal morphology and trafficking in intact cells. Furthermore, the use of our probes for noninvasive labeling of lysosomes in cancer cells, and potentially in solid tumor tissues, will provide new avenues for characterization and investigation of lysosomal trafficking and its role in invasion, metastasis and maintenance of cancerous tissues.

## Author Contributions

L.B. designed the study. N.B.Y.; C.X. and S.R.M. carried out the synthesis of these fluorescent probes, N.B.Y.; Y.B.; P.L.; Y.X. and F.M. performed the cell assays. S.J. and N.B.Y. performed the probes characterization. L.B., K.M.G., J.J. and W.J.P. performed data analysis, wrote and edited the manuscript. All authors reviewed the manuscript.

## Supplementary Material

Supplementary Information Highly Stable and Sensitive Fluorescent Probes (LysoProbes) for Lysosomal Labeling and Tracking
