# Supplementary material for: Highly Stable and Sensitive Fluorescent Probes (LysoProbes) for Lysosomal Labeling and Tracking
Source: Sci Rep. 2015 Feb 26;5:8576. doi: 10.1038/srep08576 (PMC4341211; doi:10.1038/srep08576)
Supplement: Supplementary Information — Highly Stable and Sensitive Fluorescent Probes (LysoProbes) for Lysosomal Labeling and Tracking [file srep08576-s1.pdf]

Highly Stable and Sensitive Fluorescent Probes (LysoProbes) for Lysosomal Labeling and Tracking

Nazmiye B. Yapici, [†] Yue Bi, [‡] Pengfei Li, [‡] Xin Chen, [†] Xin Yan, [†] Srinivas Rao Mandalapu, [†] Megan Faucett, [†] Steffen Jockusch, [ζ] Jingfang Ju, [*¶] K. K. Michael Gibson, [*§] William J. Pavan, [⊥] Lanrong Bi*

†Department of Chemistry, Michigan Technological University, Houghton, MI 49931

‡School of Basic Medical Sciences, HeBei Medical University, Shijiazhuang, China 050000

ζDepartment of Chemistry, Columbia University, NY 10027

¶Translational Research Laboratory, Stony Brook Medicine, Stony Brook, NY 11794

§ College of Pharmacy, Washington State University, Spokane WA 99202

National Human Genome Research Institute, NIH, Bethesda, Maryland 20892

SUPLEMENTARY INFORMATION:

EXPERIMENTAL SECTION:

Cell Culture: HeLa cells were obtained from American Type Cell Culture collection (ATCC). HeLa cells were grown in Eagle's Minimal Essential Medium (EMEM) and 10% FBS (Sigma-Aldrich, heat inactivated). All cells were maintained in a 5% CO_2 humidified atmosphere at 37°C. Human cholangiocarcinoma RBE cells were maintained in RPMI-1640 (Gibco BRI, Carlsbad, CA, USA) supplemented with 10% fetal bovine serum (Gibco) and 100 units/mL penicillin and 0.1mg/mL streptomycin (Penicillin and Streptomycin Solution (100×), Gibco, USA) at 37°C in a humidified atmosphere containing 5% CO_2.

Live cell imaging: Cells were grown in 35 mm glass bottom dishes for 24 h in media. The media was removed and cells were washed three times with 1X DPBS without Ca^{2+} or Mg^{2+} (Hyclone, Fisher Sci.). LysoProbes **I-VI** were incubated with cells in non-FBS media. After each step, cells were washed with DPBS buffer. Cells were imaged using an Olympus confocal laser-scanning microscope.

Immunoblotting: Cells were lysed in lysis buffer (30 mM Tris-HCl pH 8.0, 150 mM NaCl, 1% NP-40, 1 mM phenylmethylsulfonylfluoride and protease inhibitor cocktail) on ice for 30 min, centrifuged at 14000 g for 15 min at 4 °C, and the supernatants were collected. Equal amounts of protein from each sample were separated by SDS-PAGE and transferred to nitrocellulose membranes (Bio-Rad, Marnes-la-Coquette, France). Following incubation with antibodies against microtubule-associated protein 1 light-chain 3 (LC3), p62 and actin (Cell Signaling Technology, Beverly, MA) as primary antibodies and peroxidase-conjugated goat anti-rabbit IgG (Jackson IP Laboratories, West Grove, PA) as a secondary antibody, specific protein bands were visualized using enhanced chemiluminescence reagents for Western blot analysis (Amersham Pharmacia Biotech, Piscataway, NJ). The signal intensity was determined by densitometry and the results were presented relative to control values, which was set to 1.

Western Blotting Assay: Western blotting was performed as previously described. In brief, the cells were lysed by sonication in RIPA buffer. Protein concentrations were measured by BCA (Thermo scientific, 23228). Proteins were loaded onto standard SDS–PAGE gels and transferred to polyvinylidene difluoride membranes, which were detected with the primary antibodies: anti-LC3 rabbit polyclonal (dilution, 1:1000; Abcam, ab48394); anti-β-actin (1:2000 dilution; Sigma) antibodies followed by treatment with horseradish peroxidase-conjugated goat anti-mouse IgG (1:10000 dilution; Pierce, Woodstock, IL, USA). For p62 immunocytochemistry, treatment with anti-p62 (1:500, Santa Cruz Biotech, Santa Cruz, CA) was followed by treatment with horseradish peroxidase-conjugated goat anti-mouse IgG (1:10000 dilution; Pierce, Woodstock, IL, USA).

Expression of Green Fluorescent Protein-LC3: The green fluorescent protein (GFP)-human LC3 fusion protein expressing plasmid pEGFP-LC3 was purchased from Life Technologies. RBE cells (5×10^4 per well) were seeded in six-well plates the day before transfection and 2 μg of plasmid was transfected with FuGENER HD Transfection Reagent (Roche, 04709691001). GFP fusion proteins were observed under a laser scanning microscope system. The percentage of GFP-LC3-positive cells with GFP-LC3 punctate patterning was determined from three independent experiments and the means derived.

Statistical Analysis: The data were expressed as means ± S.D. Statistical analysis was performed with Student's t-test or by one-way ANOVA followed by Dunnett's test for multiple comparisons. Statistical significance was set at $P<0.05$.

Intracellular localization of LysoProbes:

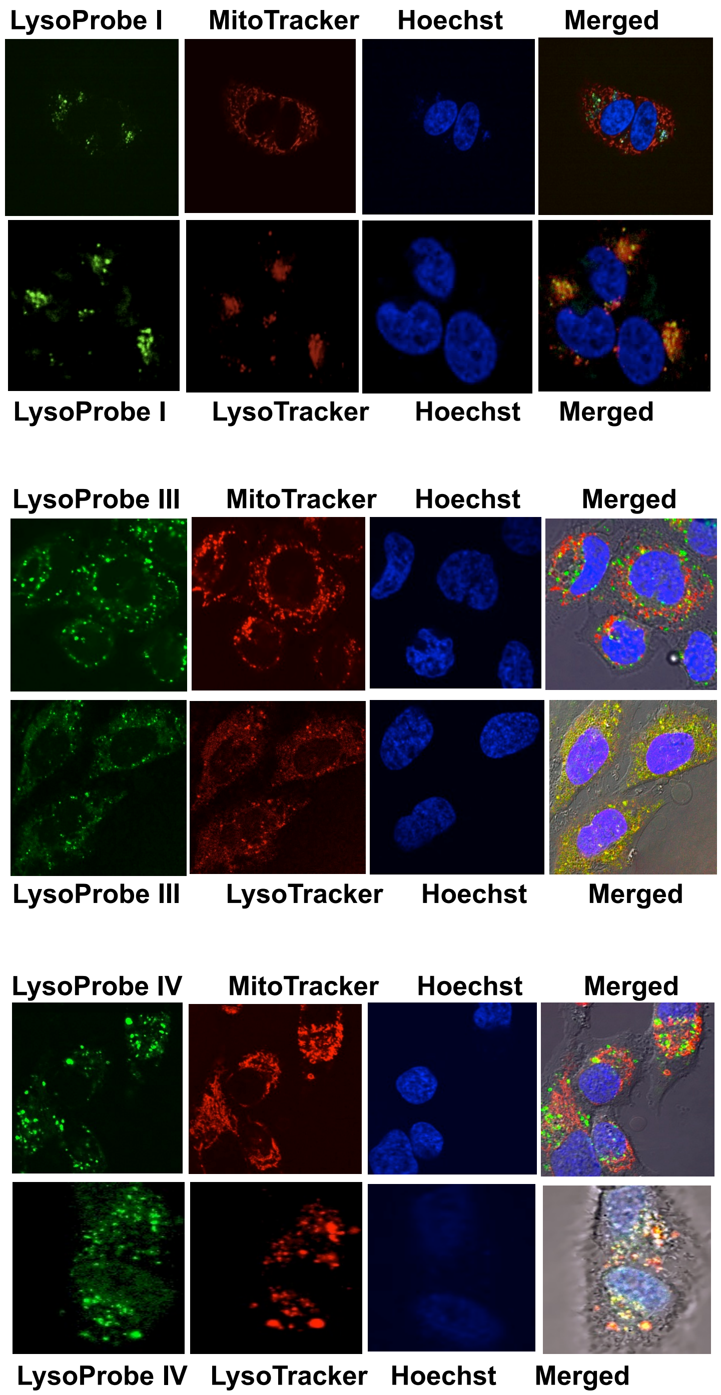

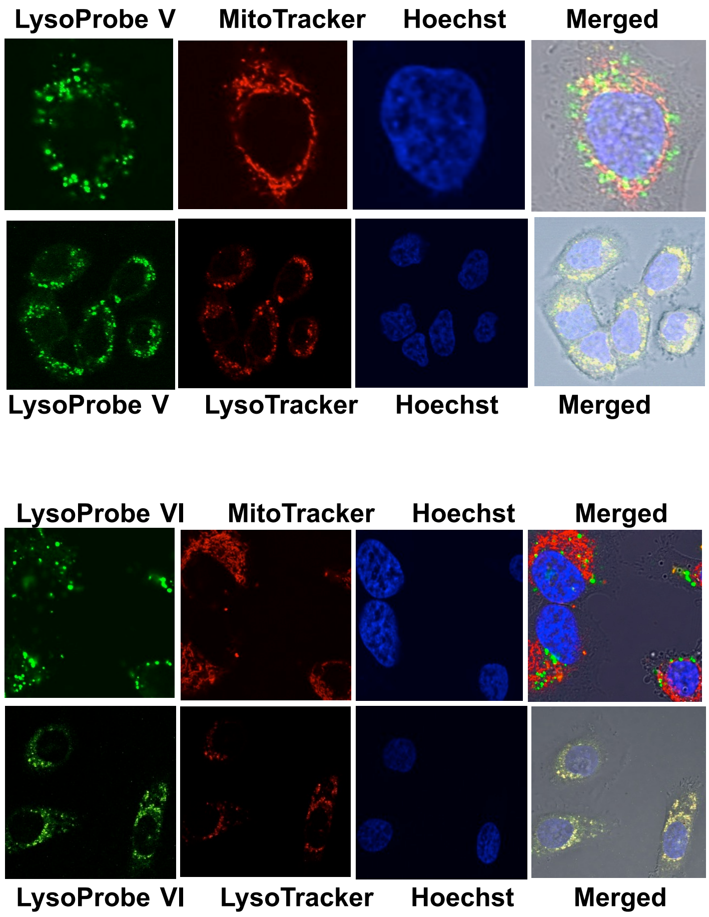

Figure S1. Confocal laser-scanning fluorescent images of LysoProbes in HeLa cells. LysoProbe (30 μM, green) was incubated with cells in non-FBS DMEM media for 45-min and counterstained with MitoTracker (80 nM, red), Hoechst 33342 (1 μg/mL, blue); and overlay (D); HeLa cells incubated with LysoProbes (20 μM) in media for 45min, followed by counterstain with LysoTracker (2 μM), Hoechst 33342 (1 μg/mL); and overlay; All images were acquired with an 60 X objective.

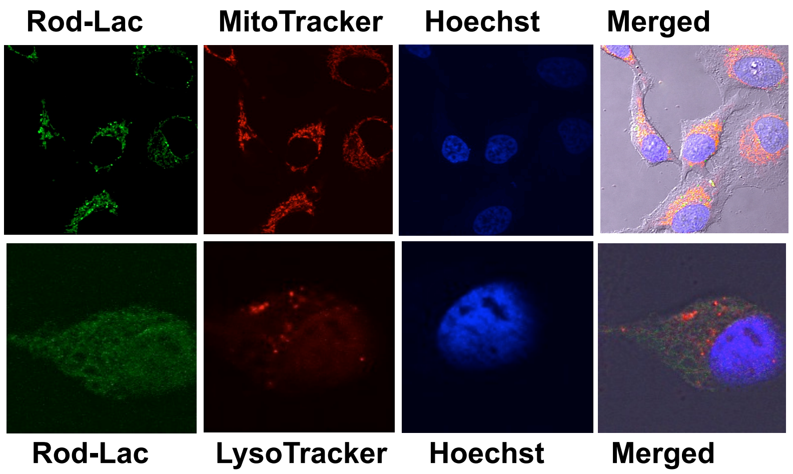

Figure S2. Confocal laser-scanning fluorescent images of Rhodamine-lactose conjugate I in HeLa cells. Top row: Rhodamine-lactose conjugate I (20 μM, green) was incubated with cells in non-FBS DMEM media for 45-min and counterstained with MitoTracker (40 nM, red), Hoechst 33342 (1 μg/mL, blue); and merged images. Bottom row: HeLa cells incubated with Rhodamine-lactose conjugate I (20 μM, green) in media for 15-min, followed by counterstain with LysoTracker (2 μM, red), Hoechst 33342 (1 μg/mL, blue); and merged images; All images were acquired with an 60 X objective.

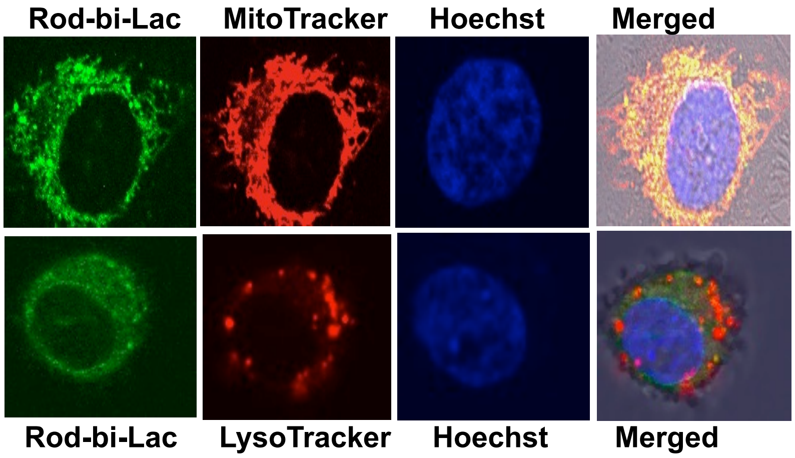

Figure S3. Confocal laser-scanning fluorescent images of Rhodamine-bi-lactose conjugate II in HeLa cells. Top row: Rhodamine-bi-lactose conjugate II (20 μM, green) was incubated with cells in non-FBS DMEM

media for 45-min and counterstained with MitoTracker (40 nM, red), Hoechst 33342 (1 μg/mL, blue); and merged images. Bottom row: HeLa cells incubated with Rhodamine-bi-lactose conjugate II (20 μM, green) in media for 15-min, followed by counterstain with LysoTracker (2 μM, red), Hoechst 33342 (1 μg/mL, blue); and merged images; All images were acquired with an 60 X objective.

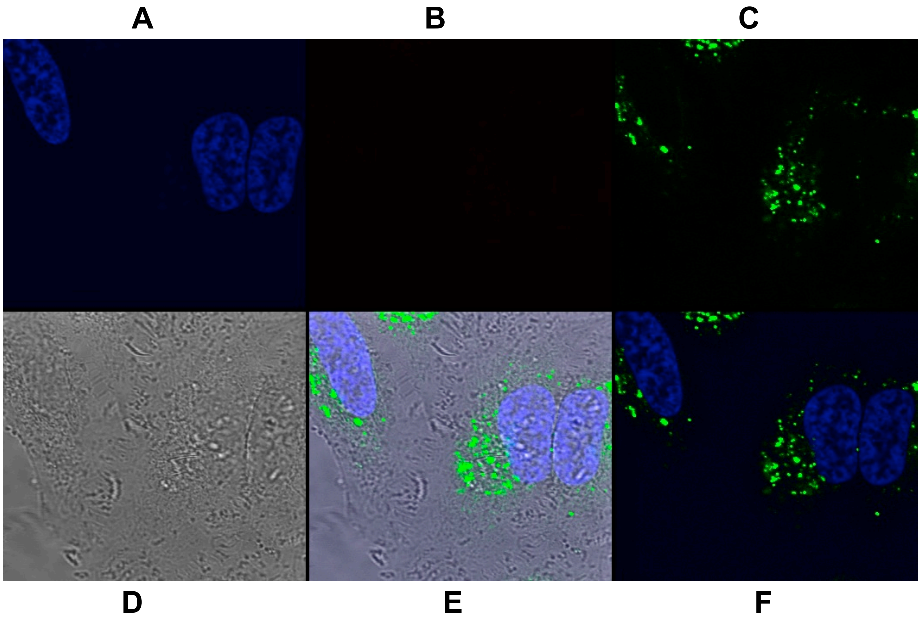

Figure S4: Confocal laser-scanning fluorescent images of LysoProbe II in HeLa cells. LysoProbe II (20 μM, green, C) was incubated with cells in non-FBS DMEM media for 15 min., and then counterstained with Lyso-Tracker (2 μM, not fluorescence, B), Hoechst 33342 (1 μg/mL, blue, A); DIC image (D), overlay (A+B+C) with DIC (E), overlay (A+B+C) without DIC image (F). All images were acquired with an 60 X objective after 24h incubation.

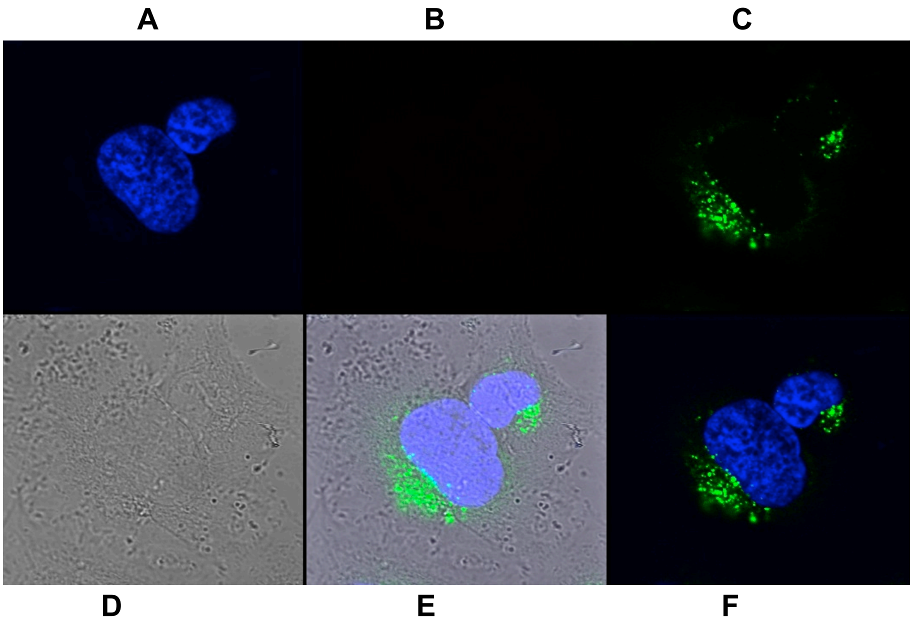

Figure S5: Confocal laser-scanning fluorescent images of LysoProbe III in HeLa cells. LysoProbe III (20 μM, green, C) was incubated with cells in non-FBS DMEM media for 15 min., and then counterstained with Lyso-Tracker (2 μM, not fluorescence, B), Hoechst 33342 (1 μg/mL, blue, A); DIC image (D), overlay (A+B+C) with DIC (E), overlay (A+B+C) without DIC image (F). All images were acquired with an 60 X objective after 24h incubation.

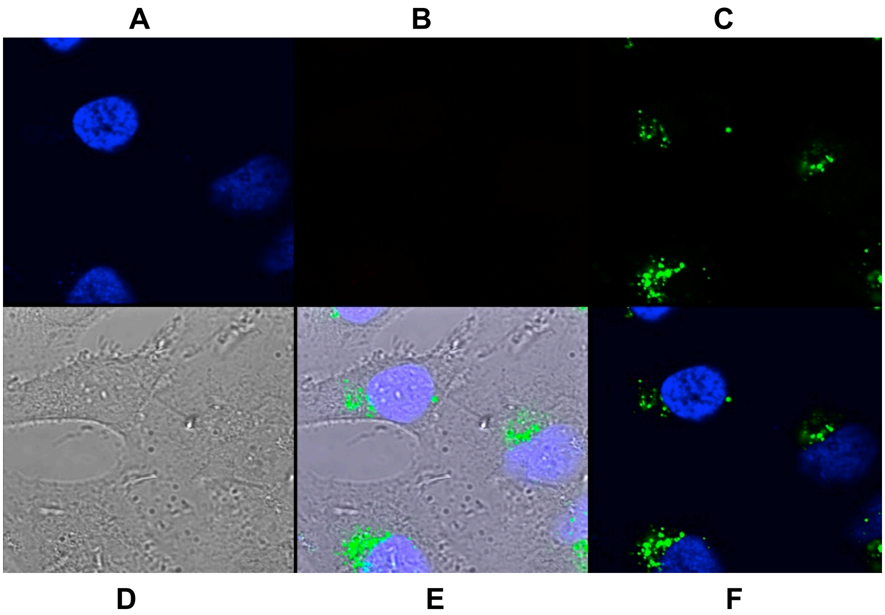

Figure S6: Confocal laser-scanning fluorescent images of LysoProbe IV in HeLa cells. LysoProbe IV (20 μM, green, C) was incubated with cells in non-FBS DMEM media for 15 min., and then counterstained with Lyso-Tracker (2 μM, not fluorescence, B), Hoechst 33342 (1 μg/mL, blue, A); DIC image (D), overlay (A+B+C) with DIC (E), overlay (A+B+C) without DIC image (F). All images were acquired with an 60 X objective after 24h incubation.

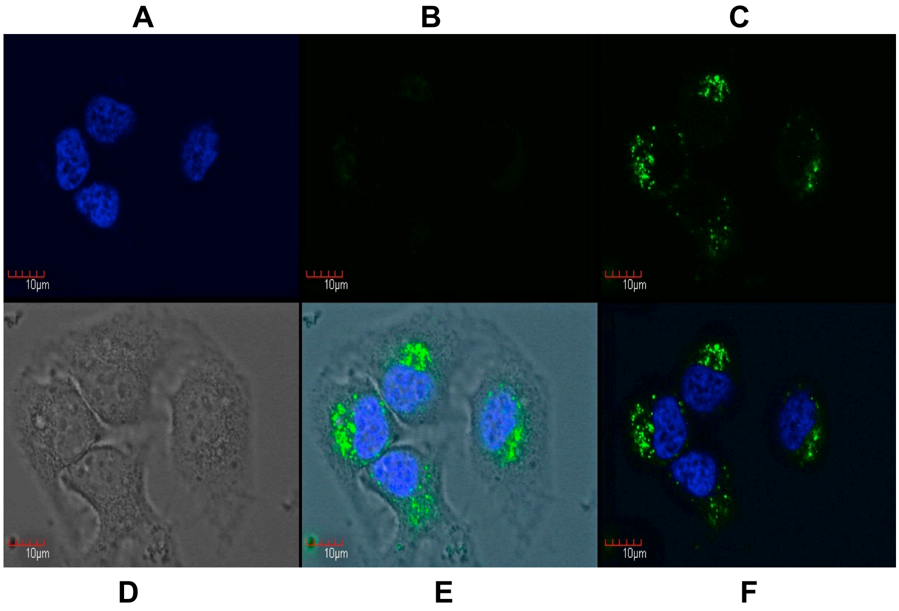

Figure S7: Confocal laser-scanning fluorescent images of LysoProbe V in HeLa cells. LysoProbe V (20 μM, green, C) was incubated with cells in non-FBS DMEM media for 15 min., and then counterstained with Lyso-Tracker (2 μM, not fluorescence, B), Hoechst 33342 (1 μg/mL, blue, A); DIC image (D), overlay (A+B+C) with DIC (E), overlay (A+B+C) without DIC image (F). All images were acquired with an 60 X objective after 24h incubation.

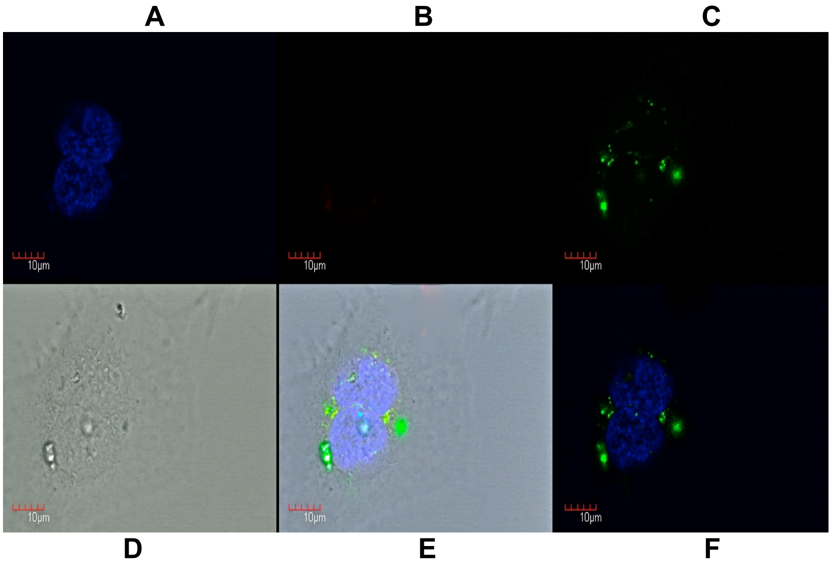

Figure S8: Confocal laser-scanning fluorescent images of LysoProbe VI in HeLa cells. LysoProbe VI (20 μM, green, C) was incubated with cells in non-FBS DMEM media for 15 min., and then counterstained with Lyso-Tracker (2 μM, not fluorescence, B), Hoechst 33342 (1 μg/mL, blue, A); DIC image (D), overlay (A+B+C) with DIC (E), overlay (A+B+C) without DIC image (F). All images were acquired with an 60 X objective after 24h incubation.

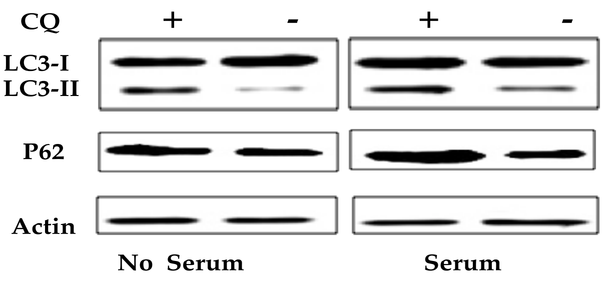

Figure S9. RBE cells were incubated in medium with or without standard serum, in the absence or presence of chloroquine (20 μM). Immunoblot analysis of LC3, p62 and actin was performed after 16h. Representative immunoblots are shown.

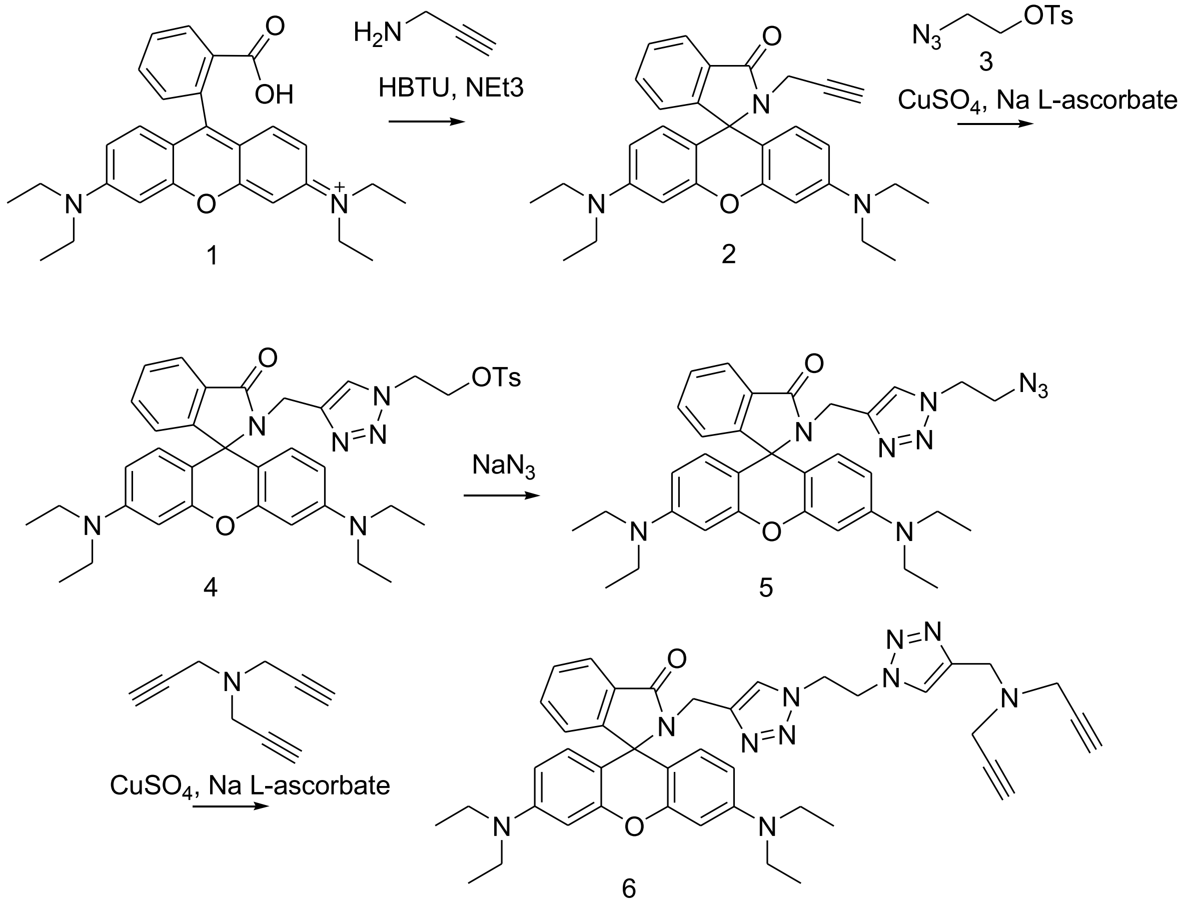

Scheme S1. Synthesis of LysoProbe precursors.

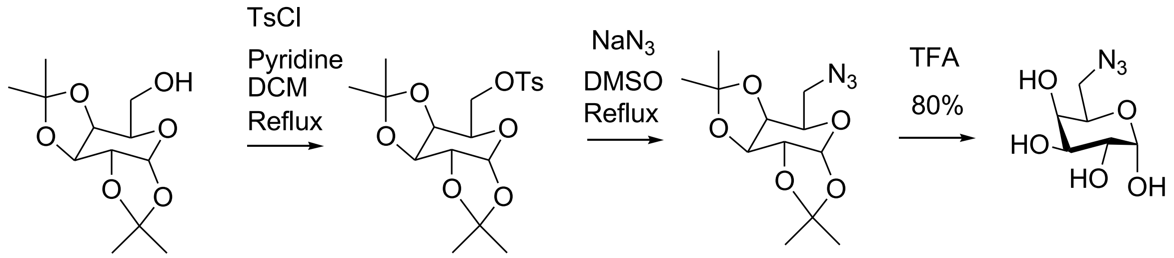

7 **8** **9**

Scheme S2. Synthesis of 6-Azido-6-deoxy-α-D-galactose (compound 9)

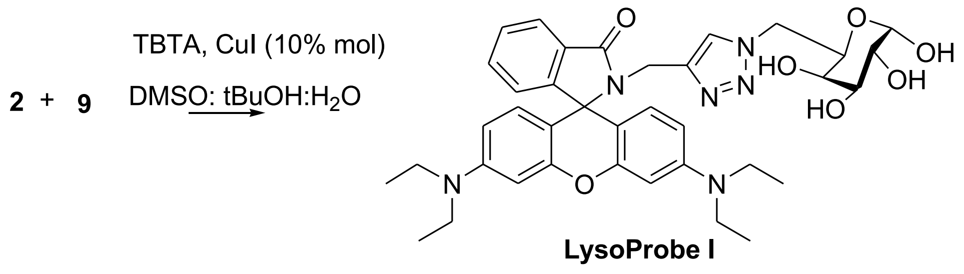
2 + 9
TBTA, CuI (10% mol)
DMSO: tBuOH:H_2O
LysoProbe I

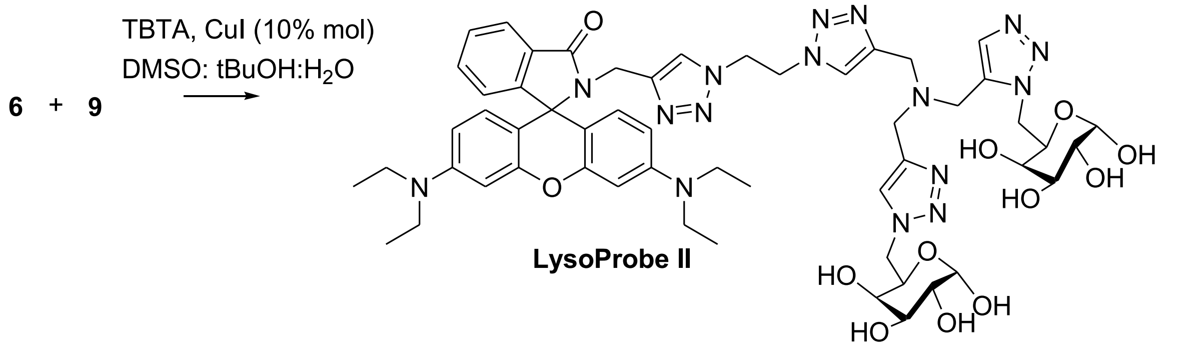
6 + 9
TBTA, CuI (10% mol)
DMSO: tBuOH:H_2O
LysoProbe II

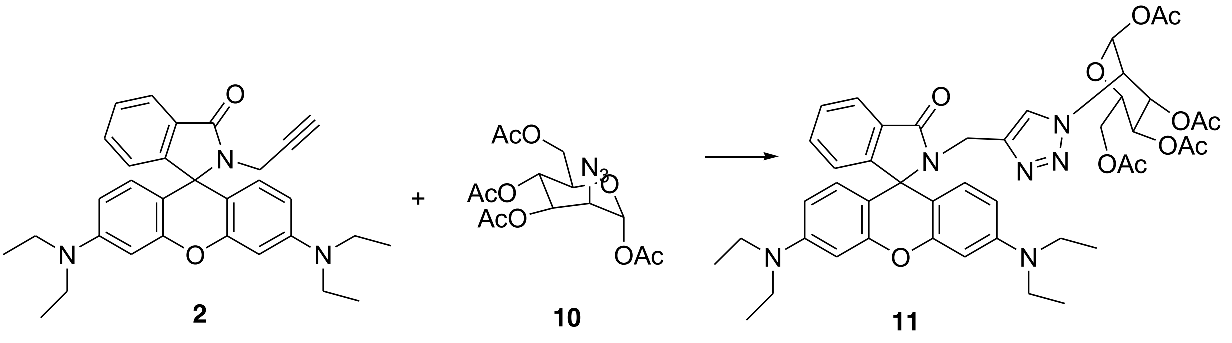
2
10
11

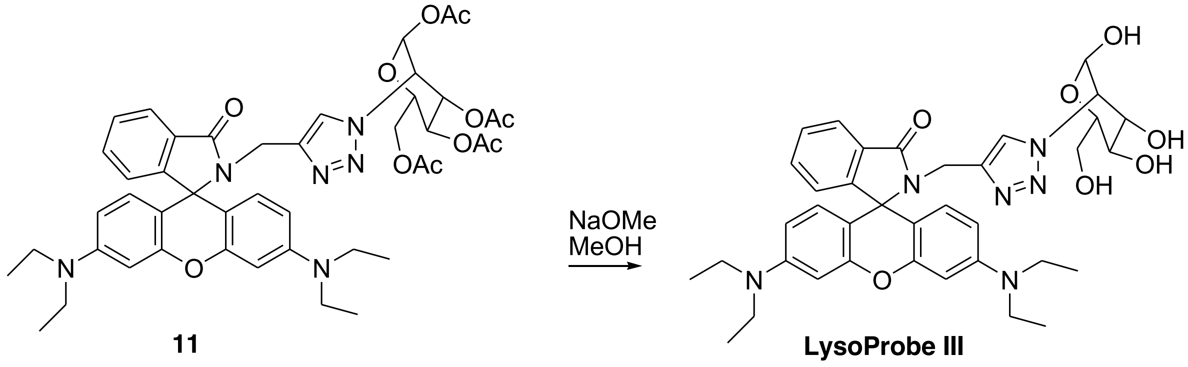
11
NaOMe
MeOH
LysoProbe III

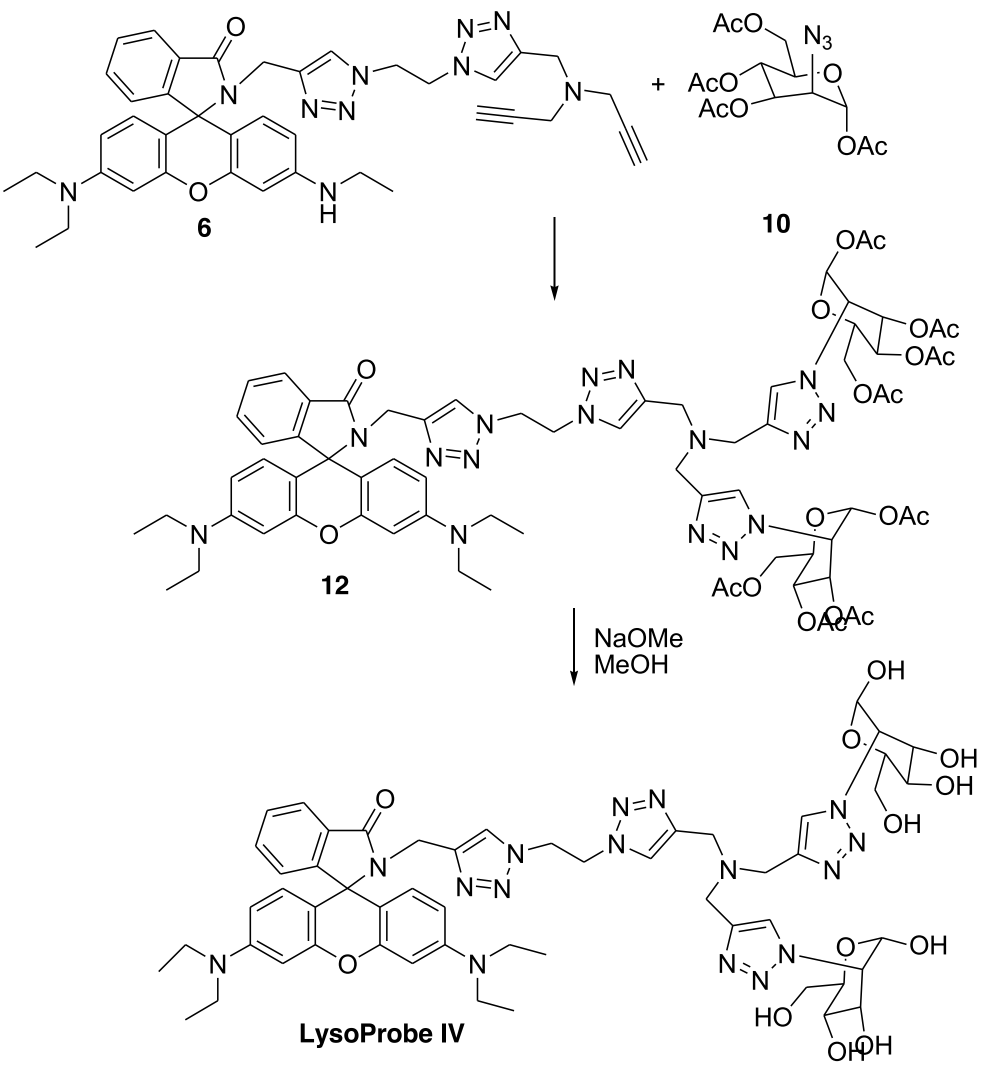
6
10
12
NaOMe
MeOH
LysoProbe IV

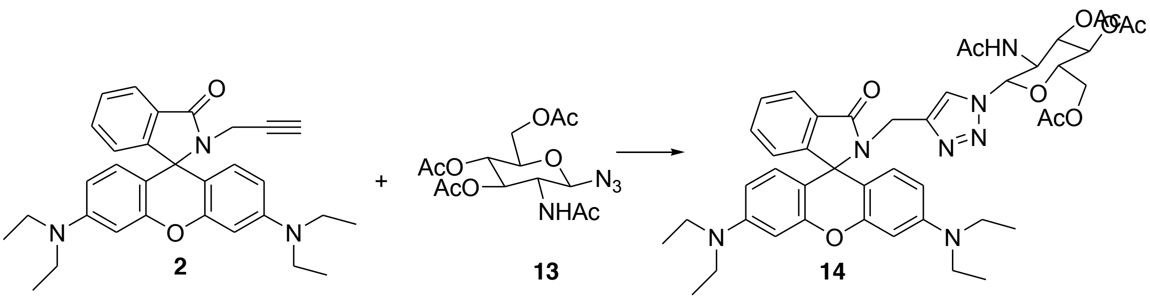
2
13
14

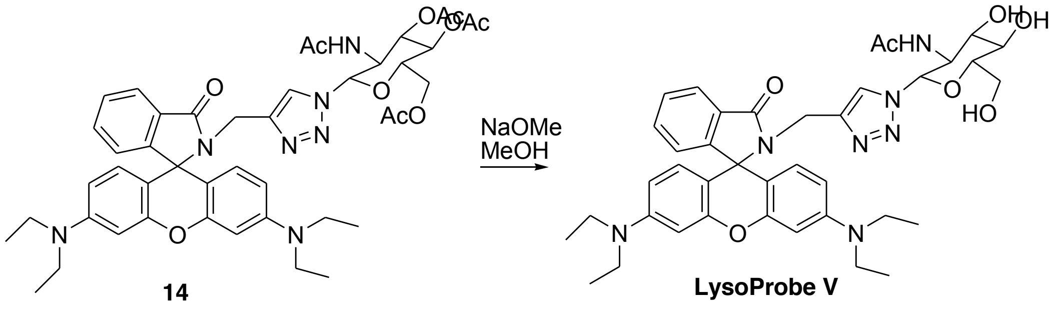
14
NaOMe
MeOH
LysoProbe V

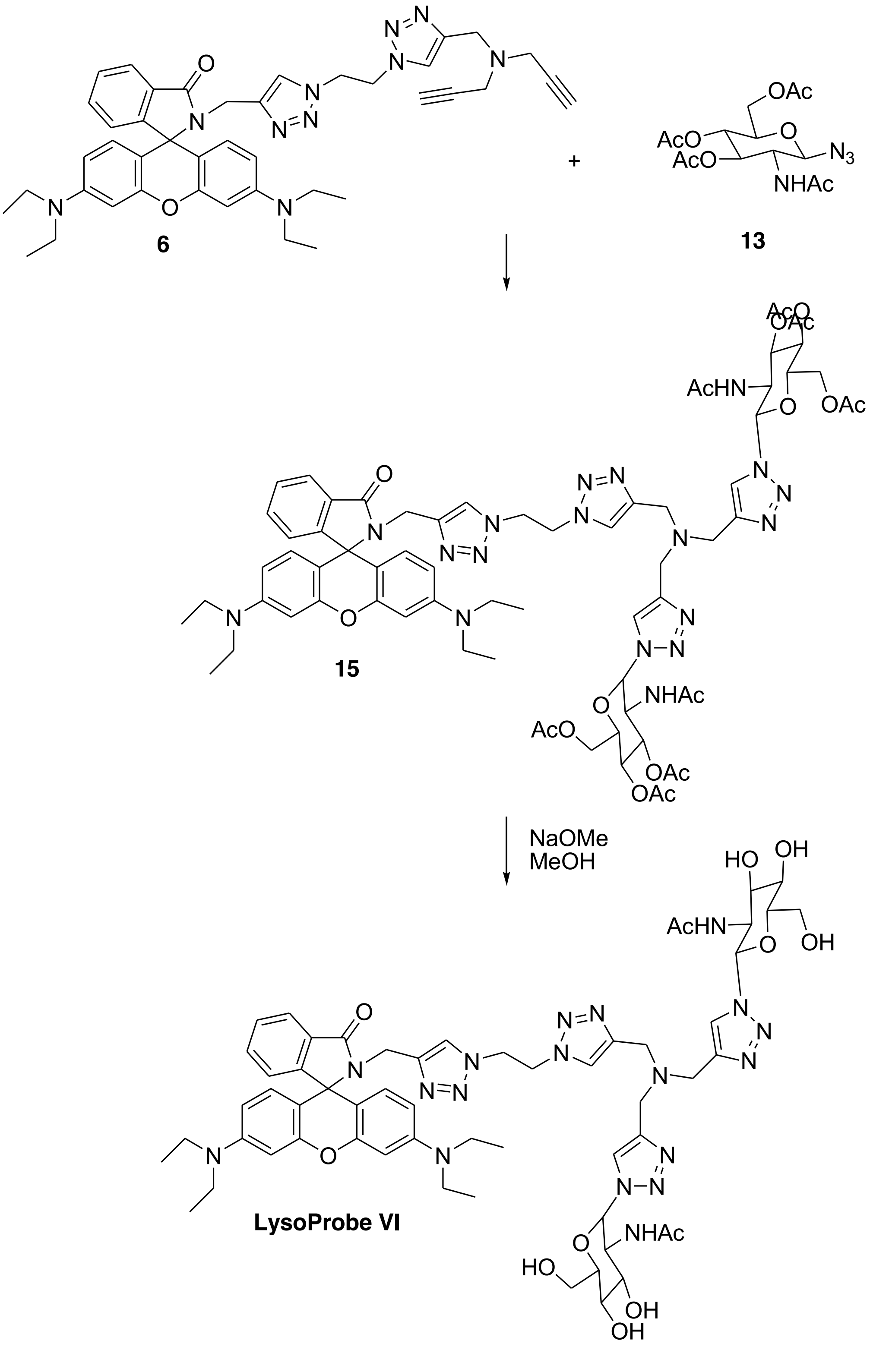

Scheme S3. Synthesis of LysoProbes **I-VI.**

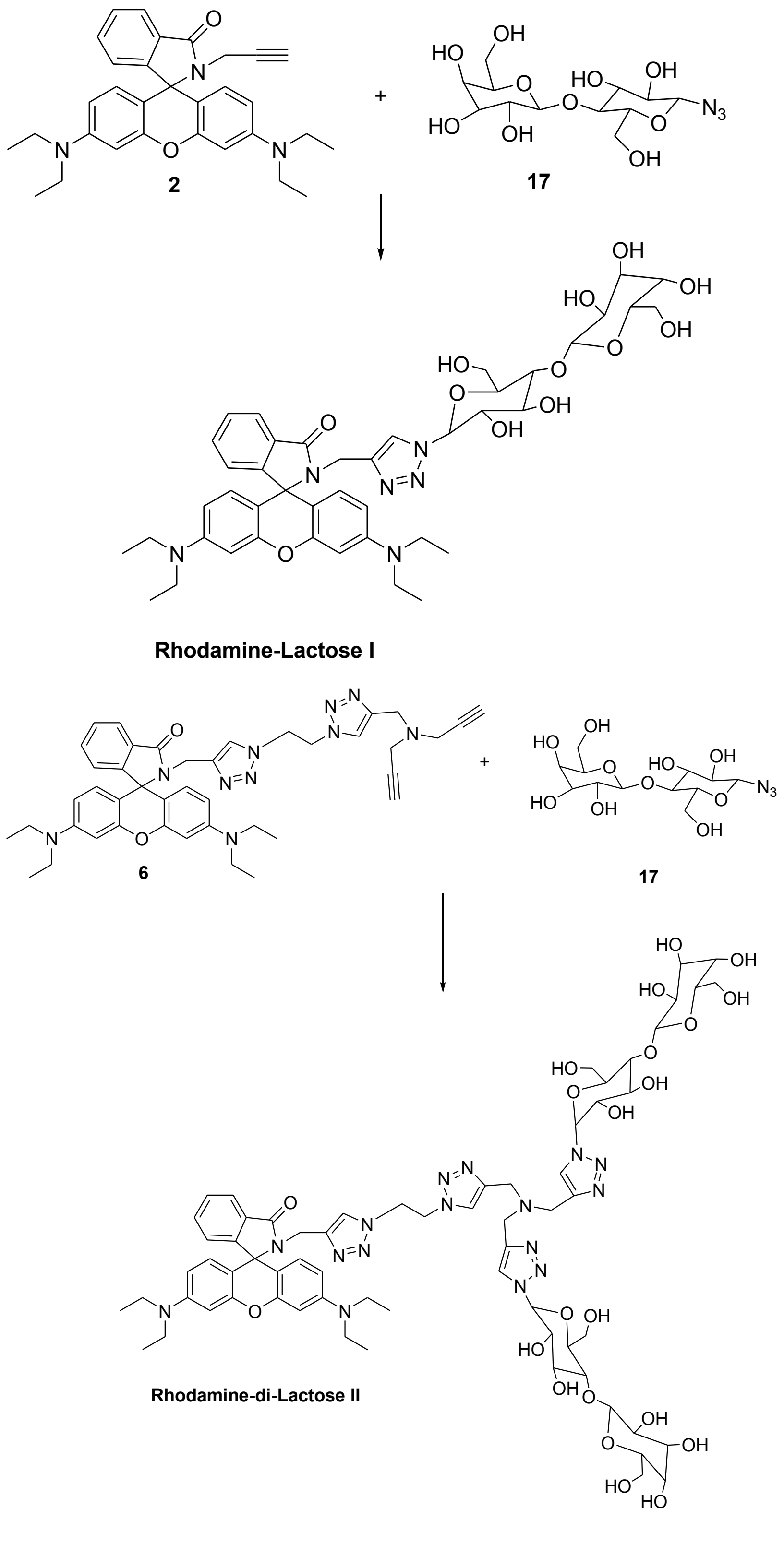

Scheme S4. Synthesis of the control compounds, Rhodamine-Lactose I and Rhodamine-di-Lactose II.

Reagents and solvents available from commercial sources were used as received unless otherwise noted. Thin layer chromatography (TLC) was performed using Sigma-Aldrich TLC plates, silica gel 60F-254 over glass support, 0.25μm thickness. Flash column chromatography was performed using Alfa Aesar silica gel, particle size 230-400 mesh. 1H and 13C NMR spectra were measured by using a Varian UNITY INOVA instrument at 400 MHz and 100 MHz respectively. The chemical shifts (δ) were reported in reference to solvent peaks (residue CHCl3 at δ 7.24 ppm for 1H and CDCl3 at δ 77.00 ppm for 13C). High-resolution mass spectra (HR-MS) were obtained on a JEOL JMS HX 110A mass spectrometer.

3',6'-bis(diethylamino)-2-(prop-2-ynyl)spiro[isoindoline-1,9'-xanthen]-3-one(**2**): To a stirring solution of rhodamine B (5.76g, 12mmol) in 20mL anhydrous dichloromethane, 2-(1H-Benzotriazol-1-yl)-1,1,3,3-tetramethyluronium hexafluoro-phosphate, HBTU) (5.45g, 14.4 mmol), propargylamine (0.925mL, 14.4 mmol) and tri-ethylamine (2.5mL, 14.4mmol) were added. The reaction mixture was continuously stirred at room temperature until TLC indicated that the starting material was absent. The reaction mixture was diluted with dichloromethane and then washed with brine. The combined organic layers were dried over anhydrous sodium sulfate, filtered and evaporated under reduced pressure. The residue was purified by flash column chromatography on silica gel (Hexane:EtOAc, 4:1) to yield compound **2** as a light pink solid (4.25g, 8.85mmol) in 74 % yield. ^{1}H NMR (400 MHz, $CDCl_3$) δ ppm 1.14 (t, J= 7.2, 12 H), 1.74 (t, J= 2.4, 1H), 3.32 (q, J= 7.2 Hz, 8H), 3.93 (d, J= 2.4 Hz, 2H), 6.25 (dd, J= 2.8, 8.8, 2H), 6.37 (d, J= 2.8 Hz, 2H), 6.45 (d, J= 8.8 Hz, 2H), 7.10 (m, 1H), 7.41 (m, 2H), 7.91 (m, 1H). ^{13}C NMR (100 MHz, $CDCl_3$) δ ppm 12.5, 28.5, 44.3, 64.8, 70.0, 78.2, 97.8, 105.1, 108.0, 123.0, 123.7, 127.9, 129.1, 130.4, 132.6, 148.8, 153.4, 153.7, 167.4.

2-azidoethyl 4-methylbenzenesulfonate (**3**): To a stirring solution of bromoethanol (12.5g, 100mmol) in 30 mL water, sodium azide (7.8g, 120mmol) was added. The reaction mixture was gently refluxed until TLC indicated that the starting material was completely consumed. The reaction mixture was extracted with dichloromethane (25 mL × 3). The combined organic layers were dried over anhydrous Na_2SO_4, filtered and concentrated. The crude product was used directly for the next reaction step without further purification. To a solution of 2-azidoethanol in dichloromethane (50mL), triethylamine (20mL, 140mmol) and toluenesulfonyl chloride (19.6g, 100mmol) were added. The reaction mixture was stirred at room temperature for 4h and then washed with 1N NaOH (100mL×2) and dried over anhydrous Na_2SO_4, filtered and concentrated. The residue was further purified by flash column chromatography on silica gel (Hexane: EtOAc, 5:1) to yield the title compound as a colorless oil (19.2g, 80mmol, 80 % yield). ^{1}H NMR (400 MHz, $CDCl_3$) δ ppm 2.43 (s, 3H), 3.45 (t, 2H, J= 4.8 Hz), 4.13 (t, 2H, J= 4.8 Hz), 7.35-7.33 (d,

2H, J= 8.0 Hz), 7.80-7.78 (d, 2H, J= 8.0 Hz). ^{13}C NMR (100 Mhz, $CDCl_3$) δ ppm 21.6, 49.5, 68.0, 127.9, 129.9, 132.5, 145.2.

2-(4-((3',6'-bis(diethylamino)-3-oxospiro [isoindoline-1, 9'-xanthene]-2-yl)methyl)-1H-1, 2, 3-triazol-1-yl)ethyl 4-methylbenzene-sulfonate (**4**): To a stirring solution of rhodamine alkyne derivative **2** (2g, 4.16mmol) and 2-azidoethyl 4-methylbenzene-sulfonate (3, 1.8g, 5mmol) in 6mL of tert-butanol: water (1:1)), copper (II) sulfate (0.21mmol, 40mg) and sodium ascorbate (0.42 mmol, 82mg) was added. The reaction mixture was continuously stirred at room temperature for 6h until TLC indicated that the starting material was completely consumed. The reaction mixture was washed with saturated aq $NaHCO_3$ and then extracted with ethyl acetate (3 × 30mL). The combined organic layers were dried over anhydrous sodium sulfate, filtered, and concentrated. The residue was then purified by flash column chromatography on silica gel (Hexane:EtOAc, 1:4) to yield the title compound as a colorless foam (2.48g) in 84.5% yield. ^{1}H NMR (400 MHz, $CDCl_3$) δ ppm 1.09 (t, J = 7.0 Hz, 12H), 2.32 (s, 3H), 3.26 (q, J = 7.0 Hz, 8H), 4.21 (t, J = 5.0 Hz, 2H), 4.29 (t, J = 5.0 Hz, 2H), 4.37 (s, 1H), 6.12 (dd, J = 8.8, 2.0 Hz, 2H), 6.24 (d, J = 8.8 Hz, 2H), 6.31 (d, J = 2.0 Hz, 2H), 6.84 (s, 1H), 7.10-6.94 (m, 1H), 7.21 (d, J = 8.5 Hz, 2H), 7.47-7.29 (m, 2H), 7.65-7.46 (m, 2H), 8.09-7.73 (m, 1H). ^{13}C NMR (100 MHz, $CDCl_3$) δ ppm 12.81, 21.84,35.38, 44.53, 48.60, 65.19, 67.48, 76,92, 98.01, 105.50,108.13, 123.09, 123.40, 124.14, 127.98, 128.32, 129.03, 130.26, 131.19, 132.23, 132.79, 144.76, 145.64, 148.95, 153.61, 153.64, 168.03.

2-((1-(2-azidoethyl)-1H-1, 2, 3-triazol-4-yl) methyl)-3', 6'-bis(diethylamino) spiro- [isoindoline-1, 9'-xanthen]-3-one (**5**): To a stirring solution of compound **4** (3.29g, 4.65mmol) in DMF (20ml), NaN_3 (453mg, 6.97mmol) was added. The reaction mixture was refluxed for 6h, and then washed with saturated aq $NaHCO_3$ and extracted with EtOAc (30ml × 3). The combined organic layers were dried over anhydrous Na_2SO_4, filtered and concentrated. The residue was further purified by flash column chromatography on silica gel (Hexane/EtOAc, 1/3) to yield compound **5** as a white foam (95.7%, 2.6g). ^{1}H NMR (400 MHz, $CDCl_3$) δ ppm 1.14 (t, J = 7.2 Hz, 12H), 3.30 (q, J = 7.2 Hz, 8H), 3.60 (t, J = 6.1 Hz, 2H), 4.20 (t, J = 6.1 Hz, 2H), 4.47 (s, 2H), 6.15 (dd, J = 8.8, J=2.3 Hz, 2H), 6.30 (d, J = 8.8 Hz, 2H), 6.33 (s, 2H), 7.06 (s, 1H), 7.18-7.06 (m, 1H), 7.60-7.36 (m, 2H), 7.90-7.94 (m, 1H). ^{13}C NMR (100 MHz, $CDCl_3$)) δ ppm 12.8, 35.5, 44.5, 48.9, 50.6, 65.2, 98.1, 105.6, 108.0, 123.1, 123.2, 124.1, 128.3, 129.0, 131.2, 132.8, 144.9, 148.9, 153.6, 153.7, 168.1. HR/MS $[M+H]^+$ Calcd: 592.3148; Found: 592.3160.

3', 6'-bis(diethylamino)-2-((1-(2-(4-((diprop-2-ynylamino) methyl)-1H-1, 2, 3-triazol-1-yl) ethyl)-1H-1,2,3-triazol-4-yl) methyl) spiro [isoindoline-1, 9'-xanthen]-3-one (**6**): To a stirring solution of **5** (4g, 6.92mmol) and tripropargylamine (1.8g, 13.8mmol) in 6mL of t-BuOH: water (1:1)), $CuSO_4$ (5% mol, 60mg) and sodium ascorbate (10%mol, 160mg) were added. The reaction mixture was continuously stirred at room temperature for 6h, subsequently washed with saturated aq. $NaHCO_3$, and then extracted with EtOAc

(30mL × 3). The combined organic layers were dried over anhydrous Na_2SO_4, filtered and concentrated. The residue was further purified by flash column chromatography on silica gel (EtOAc:MeOH, 20:1) to yield **6** as a white foam (28%, 1.4g). ^{1}H NMR (400 MHz, $CDCl_3$) δ ppm 1.11 (t, J = 7.0 Hz, 12H), 2.18 (q, J = 2.2 Hz, 2H), 3.27 (q, J = 7.0 Hz, 8H), 3.33 (d, J = 2.2 Hz, 4H), 3.71 (s, 2H), 4.37 (s, 2H), 4.52 (t, J = 6.0 Hz, 2H), 4.68 (t, J = 6.0 Hz, 2H), 6.13 (dd, J = 8.9, J=2.4 Hz, 2H), 6.24 (d, J = 8.9 Hz, 2H), 6.30 (s, 2H), 6.62 (s, 1H), 7.03-7.06 (m, 1H), 7.15 (s, 1H), 7.65-7.34 (m, 2H), 7.84-7.991 (m, 1H). ^{13}C NMR (100 MHz, $CDCl_3$)) δ ppm 12.7, 35.2, 42.1, 44.5, 47.8, 49.4, 49.5, 65.1, 73.7, 78.7, 97.9, 105.6,108.1, 123.1, 123.4, 124.1, 124.2, 128.4, 129.1, 131.7, 132.8, 144.8, 144.9, 148.9, 153.4,153.6, 168.0. HR/MS $[M+H]^+$ Calcd: 723.3883; Found: 723.3911.

p-Toluenesulfonyl-1,2:3,4-di-O-isopropyliden-D-galactopyranose (**7**): To a solution of 1,2:3,4-Di-O-isopropyliden-D-galactopyranose (1.8g, 70 mmol) in pyridine (anhydrous, 8ml) and CH_2Cl_2 (anhydrous, 4 ml) under nitrogen gas, p-toluenesulfonyl chloride (2.8 g, 147 mmol) was added dropwise, followed by addition of DMAP (catalytic amount) with overnight reflux. Water (1 mL) and toluene were added to the above reaction mixture and evaporated. The residue was dissolved in CH_2Cl_2 and extracted with saturated aqueous $NaHCO_3$. The combined organic layer was dried over Na_2SO_4, then filtered and concentrated. The residue was purified by flash chromatography using EtOAc: Hexane (1:3) to give the title compound as a syrup in 78.5% yield (2.27g). ^{1}H NMR (400 MHz, $CDCl_3$) δ ppm 1.25 (s, 3H), 1.29 (s, 3H), 1.32 (s, 3H), 1.47 (s, 3H), 2.42 (s, 3H), 4.06 (m, 2H), 4.18 (m, 2H), 4.27 (m, 1H), 4.56 (m, 1H), 5.43 (d, J = 4.87 Hz, 1H), 7.30 (d, J = 8.58 Hz, 2H), 7.78 (dd, J = 8.26 Hz, J =1.32, 2H).

6-Azido-6-deoxy-1,2:3,4-di-O-isopropylidene-α-D-galactopyranose (**8**): To a stirring solution of compound **7** (2.27g, 55 mmol) in DMF (anyhdrous, 20ml), NaN_3 (712 mg, 111 mmol) was added and then refluxed under nitrogen atmosphere for 18h. The reaction mixture was extracted with $NaHCO_3$ and CH_2Cl_2. The combined organic layers were dried over Na_2SO_4 and concentrated. The residue was further purified by flash chromatography using EtOAc:Hex (1:4) to give the title compound (8) as a syrup in 64% yield (1g). ^{1}H NMR (400 MHz, CDCl3) δ ppm 1.29 (d, J = 2.27 Hz, 6H), 1.41 (s, 3H), 1.50 (s, 3H), 3.31 (dd, J = 12.71, 5.28 Hz, 1H), 3.46 (dd, J = 12.70, 7.90 Hz, 1H), 3.86 (ddd, J = 7.34, 5.28, 1.92 Hz, 1H), 4.14 (dd, J = 7.88, 1.92 Hz, 1H), 4.29 (ddd, J = 5.03, 2.49, 0.74 Hz, 1H), 4.58 (dd, J = 7.88, 2.46 Hz, 1H), 5.50 (d, J = 5.02 Hz, 1H). ^{13}C NMR (100 MHz, $CDCl_3$): δ ppm 24.6, 25.1, 26.1, 26.2, 50.8, 67.2, 70.5, 70.9, 71.3, 96.5, 108.9, 109.8.

6-azido-6-deoxy-D-galactopyranose (**9**): Compound **8** (200mg, 70mmol) was dissolved in 80% TFA and then stirred for 30 min. The reaction mixture was concentrated and then co-evaporated three times with H_2O and EtOAc, respectively. Compound **9** was obtained as an off-white solid (140 mg, 68mmol, 97% yield). ^{1}H NMR (400 MHz, D_2O) δ ppm 3.66-3.13 (m, 2H), 3.83 (ddd, J = 25.27, 10.34, 3.52 Hz, 2H), 3.96 (d, J = 2.95 Hz, 1H), 4.20 (dd, J = 7.91, 4.68 Hz, 1H), 5.27 (d, J = 3.74 Hz, 1H).

LysoProbe I: To a solution of compound **2** (1mmol) in DMSO: t-BuOH: H_2O (8:4:7), CuI (0.01mmol), TBTA (0.01mmol) and compound **9** (1.2mmol) were added and stirred overnight. The reaction mixture pH was adjusted to neutrality and extracted with CH_2Cl_2. The combined organic layer was dried over Na_2SO_4 and evaporated. The residue was purified by fluorisil using EtOAc:Hex: DCM (3:1:1). The title compound was obtained as a light yellow solid in 71% yield. ^{1}H NMR (400 MHz, CD_3OD) δ ppm 1.11 (t, J = 6.87 Hz, 12H), 3.31 (q, 8H), 3.42-3.48 (m, 1H), 3.73 (s, 2H), 4.12-4.64 (m, 4H), 6.18 (bs, 4H), 6.34 (bs, 2H), 6.94 (d, J = 6.68 Hz, 1H), 7.21-7.30 (m, 1H), 7.42-7.49 (m, 2H), 7.84-7.90 (m, 1H). ^{13}C NMR (100 MHz, CD_3OD): δ ppm 11.8, 34.5, 44.2, 51.1, 65.7, 68.8,68.9, 69.4,69.7, 70.2, 72.2, 73.5, 93.03, 97.4, 97.8, 104.7, 108.1, 108.2, 122.6, 123. 9, 128.4, 128.5, 128.6, 130.7, 133.1, 149.1, 153.6, 153.7, 168.4. HR/MS $[M+H]^+$ Calcd: 685.3350; Found: 685.3358.

LysoProbe II: To a solution of compound **6** (1mmol) in DMSO: t-BuOH: H_2O (8:4:7), CuI (0.02mmol), TBTA (0.02mmol) and compound **9** were added and continuously stirred overnight. The reaction mixture pH was adjusted to neutrality and then concentrated. The residue was purified on florisil (60-100 mesh, fisher scientific) using MeOH: DCM (1:25) and then MeOH: DCM (50:50). The title compound was obtained as a yellow syrup in 25% yield. ^{1}H NMR (400 MHz, CD3OD) δ ppm 1.12 (t, J = 6.96 Hz, 12H), 3.32 (q, J= 7.34 Hz, 8H), 3.50 (m, 1H), 3.63 (s, 6H), 3.77 (dd, J = 4.63, 3.04 Hz, 2H), 3.84 (d, J = 3.06 Hz, 1H), 3.94 (m, 1H), 4.27 (s, 2H), 4.38 (dd, J = 8.64, 5.14 Hz, 2H), 4.60 (m, 6H), 4.76 (m, 2H), 5.12 (d, J = 3.25 Hz, 1H), 6.19 (dd, J = 28.17, 9.51 Hz, 4H), 6.34 (d, J = 2.47 Hz, 2H), 7.02 (m, 2H), 7.49 (m, 2H), 7.65 (m, 1H), 7.84 (m, 1H), 7.93 (m, 2H). 13C NMR (100 MHz, CD3OD)) δ ppm 11.7, 34.3, 44.2, 65.6, 68, 9, 69.0, 69.1, 69.4, 69.7, 70.1, 72.3, 73.5, 91.4, 92.5, 93.1, 97.5, 97.8, 104.7, 108.2, 122.6, 123.7, 123.8, 124.9 125.2, 125.4, 130.6, 133.1, 149.1, 153.6, 153. 7, 168.4. MS $[M+H]^+$ Calcd: 1133.5203; Found: 1133.5374.

LysoProbe III: To a solution of compound **2** (0.643 mmol) in DMSO: t-BuOH: H_2O (8:4:7), $CuSO_4$, Na ascorbate and TBTA (0.06 mmol) were added. Then 1,3,4,6-tetra-O-acetyl-2-azido-2-deoxy-α-D-mannopyranose (**10**, 0.536 mmol) was added and stirred overnight. The reaction mixture pH was adjusted to neutrality and then extracted with CH_2Cl_2. The combined organic layer was dried over Na_2SO_4 and evaporated. The residue was purified on fluorisil using EtOAc:Hex (1:2). Compound **11** was obtained in 32 % yield. ^{1}H NMR (400 MHz, $CDCl_3$) δ ppm 1.03 (m, 12H), 1.74 (d, J = 20.3 Hz, 3H), 1.90 (d, J = 3.70 Hz, 3H), 2.03 (s, 3H), 2.10 (d, J = 3.6 Hz, 3H), 3.19 (m, 8H), 4.11 (m, 3H), 4.43 (t, J = 4.3 Hz, 2H), 5.06 (d, J = 5.1 Hz, 1H), 5.13 (t, J = 10.2 Hz, 1H), 5.27 (dd, J = 10.2, 5.1 Hz, 1H), 6.18 (m, 7H), 6.95 (dd, J = 5.94, 2.71 Hz, 1H), 7.32 (m, 2H), 7.55 (s, 1H), 7.79 (dd, J = 7.5, 4.0 Hz, 1H). ^{13}C NMR (100 MHz, CD_3Cl_3) δ ppm 12.8, 20.7, 35.4, 58.9, 61.5, 62.8, 64.3, 65.0, 66.0, 68.5, 70.8,71.6, 81.7, 90.8, 92.2, 98.0, 98.3, 104.9, 105.3, 105.8, 108.0, 108.3, 122.1, 123.1, 124.1,128.1, 128.7, 129.1,130.7, 132.7, 145.1, 146.8, 148.8, 153.6, 154.1, 169.2, 170.3, 170.9, 171.1. Compound **11** was dissolved in anhydrous MeOH and NaOMe (10% by weight) was

added. After stirring overnight, the reaction mixture was neutralized with Dowex 50WX8 proton exchange resin and filtered. The crude residue was purified using fluorisil to generate LysoProbe III as a white solid. MS $[M+H]^+$ Calcd: 685.3271; Found: 685.3386.

LysoProbe IV: To a solution of compound **6** (0.387 mmol) in DMSO: t-BuOH: H_2O (8:4:7), $CuSO_4$, Na ascorbate and TBTA (0.004 mmol) were added. Then 1,3,4,6-tetra-O-acetyl-2-azido-2-deoxy-α-D-mannopyranose (**10**, 0.804 mmol) was added and stirred overnight. The reaction mixture pH was adjusted to neutrality and extracted with CH2Cl2. The combined organic layer was dried over Na_2SO_4 and concentrated. The crude product was purified on Fluorisil with MeOH:EtOAc (1:10). Compound **12** was obtained as a colorless solid in 38 % yield. ^1H NMR (400 MHz, $CDCl_3$) δ ppm 1.11 (t, J = 7.0 Hz, 12H), 1.92 (s, 6H), 2.05 (s, 6H), 2.01 (s, 6H), 2.19 (s, 6H), 3.28 (q, J = 7.0 Hz, 8H), 3.68 (s, 2H), 3.82 (dd, J = 23.5, 14.2 Hz, 4H), 4.18 (ddd, J = 8.21, 4.46, 2.28 Hz, 4H), 4.32 (dd, J = 12.25, 3.87 Hz, 4H), 4.39 (s, 2H), 4.60 (td, J = 13.73, 7.42 Hz, 4H), 5.34 (m, 2H), 5.48 (dd, J = 9.90, 5.10 Hz, 2H), 6.14 (m, 2H), 6.24 (s, 2H), 6.27 (s, 2H), 6.30 (d, J = 2.36 Hz, 2H), 6.36 (s, 2H), 6.82 (s, 1H), 7.06 (ddd, J = 5.62, 3.13, 0.67 Hz, 1H), 7.41 (dd, J = 5.62, 3.12 Hz, 2H), 7.56 (s, 1H), 7.87 (ddd, J = 5.62, 3.13, 0.67 Hz, 1H), 8.07 (s, 2H). ^{13}C NMR (100 MHz, CD_3Cl_3) δ ppm 12.8, 20.8, 21.0, 35.2, 44.5, 47.2, 48.8, 49.2, 59.5, 61.9, 64.9, 68.6, 70.9, 90.8, 97.9, 105.6, 108.0, 123.2, 123.6, 124.1, 128.3, 129.1, 131.2, 132.7, 144.6, 144.7, 145.1, 148.9, 153.4, 153.6, 167.8, 168.0, 169.4, 170.3, 170.7. Compound **12** was dissolved in anhydrous MeOH, and NaOMe (10% by weight) was added. After stirring overnight, the reaction mixture was neutralized with Dowex 50WX8 proton exchange resin and filtered. The crude product was further purified by Fluorisil to yield LysoProbe IV. ^1H NMR (400 MHz, CD_3OD) δ: 1.12 (t, *J* = 6.8 Hz, 12H), 3.15 (m, 2H), 3.34 (m,12H), 3.440-3.58 (m,4H), 3.60-3.80 (m, 12H), 3.84 (m, 2H), 4.28 (m, 2H), 4.63 (m, 2H), 4.76 (m, 2H), 5.12 (d, *J* = 8.4 Hz, 1H), 5.27 (m, 1H), 6.10-6.24 (m, 4H), 6.34 (m, 2H), 6.97 (m, 1H), 7.03 (m, 1H), 7.46 (m, 2H), 7.64 (m, 1H), 7.84 (m, 1H), 8.05 (s, 1H) , 8.12(s, 1H). MS $[M+H]^+$ Calcd: 1133.5203; Found: 1133.5546.

LysoProbe V: To a solution of compound **2** (0.967 mmol) in DMSO: t-BuOH: H_2O (8:4:7), $CuSO_4$, Na ascorbate and TBTA (0.01mmol) were added. Then 2-acetamido-3,4,6-tri-O-acetyl-2-deoxy-β-D-glucopranosyl azide (**13**, 0.793 mmol) was added and stirred overnight. The reaction mixture pH was adjusted to neutrality and then extracted with CH_2Cl_2. The combined organic layer was dried over Na_2SO_4 and evaporated. The residue was purified on Fluorisil using EtOAc:Hex (1:2). Compound **14** was obtained as a colorless solid in 46 % yield. ^1H NMR (400 MHz, $CDCl_3$) δ ppm 1.12 (m, 12H), 1.65 (s, 3H), 1.96 (s, s, s, too close peaks, 9H), 3.31 (q, J = 5.86 Hz, 8H), 4.19 (m, 6H), 5.04 (t, J = 9.70 Hz, 1H), 5.53 (t, J = 9.70 Hz, 1H), 6.02 (d, J = 9.95 Hz, 1H), 6.18 (dd, J = 8.80, 2.03 Hz, 1H), 6.35 (m, 1H), 6.97 (s, 1H), 7.04 (dd, J = 5.06, 2.19 Hz, 1H), 7.23 (dd, J = 13.49, 5.35 Hz, 1H), 7.36 (m, 2H), 7.76 (dd, J = 6.50, 4.54 Hz, 1H). ^{13}C NMR (100 MHz, CD_3Cl_3)) δ ppm 12.8, 12.9, 20.8, 22.9, 35.9, 41.3, 44.5, 53.6, 62.5 65.5, 68.7, 72.8, 74.6, 85.1,98.0, 98.3, 105.3, 108.4, 108.5, 121.7, 122.8, 124.1, 128.2, 129.1, 129.4, 131.1, 132.7, 144.8, 149.0, 153.6,

168.0, 169.7, 170.4, 170.8. To a solution of compound **14** in anhydrous MeOH, NaOMe (10% by weight) was added. The reaction mixture was continuously stirred until TLC indicated that the starting material was completely consumed, and then neutralized with Dowex 50WX8 proton exchange resin and filtered. The crude product was further purified using Fluorisil. LysoProbe V was obtained as an off-white solid in 37% yield. ^{1}H NMR (400 MHz, CD_3OD) δ ppm 1.14 (dt, J = 7.00, 3.38 Hz, 12H), 1.71 (s, 3H), 1.99 (s, 1H), 3.35 (dd, J = 13.78, 7.0 Hz, 8H), 3.46 (s, 2H), 3.67 (m, 2H), 3.86 (dd, J = 12.41, 1.46 Hz, 1H), 3.94 (t, J = 9.83 Hz, 1H), 4.22 (d, J = 15.97 Hz, 1H), 4.42 (d, J = 15.59 Hz, 1H), 5.59 (d, J = 9.83 Hz, 1H), 6.28 (m, 4H), 6.41 (dd, J = 18.78, 2.09 Hz, 2H), 7.04 (dd, J = 6.85, 2.34 Hz, 1H), 7.38 (s, 1H), 7.50 (m, 2H), 7.85 (dd, J = 6.31, 1.66 Hz, 1H). HR/MS $[M+H]^+$ Calcd: 725.3537; Found: 725.3563.

LysoProbe VI: To a solution of compound 6 (0.339 mmol) in DMSO: t-BuOH: H_2O (8:4:7), $CuSO_4$, Na ascorbate and TBTA (0.01mmol) were added. Then 2-acetamido-3,4,6-tri-O-acetyl-2-deoxy-β-D-glucopranosyl azide (**13**, 0.672 mmol) was added and continuously stirred overnight. The reaction mixture pH was adjusted to neutrality and extracted with CH_2Cl_2. The combined organic layers were dried over Na_2SO_4 and evaporated, and the crude product was further purified on fluorisil using EtOAc/MeOH (1:10). Compound **15** was obtained as a yellow solid in 43.5% yield. ^{1}H NMR (400 MHz, $CDCl_3$) δ ppm 1.10 (dt, J = 7.05, 3.16 Hz, 12H), 1.67 (s, 6H), 1.99 (m, 18H), 3.27 (dd, J = 7.02, 3.40 Hz, 8H), 3.63 (s, 6H), 4.11 (dd, J = 11.62, 5.09 Hz, 4H), 4.25 (dd, J = 13.86, 6.14 Hz, 2H), 4.31 (s, 1H), 4.57 (m, 7H), 5.21 (t, J = 9.72 Hz, 2H), 5.64 (t, J = 9.72 Hz, 2H), 6.10 (dd, J = 8.99, 2.51 Hz, 1H), 6.15 (dd, J = 8.99, 2.56 Hz, 1H), 6.25 (m, 6H), 6.64 (s, 1H), 7.04 (dd, J = 6.17, 3.00 Hz, 1H), 7.39 (m, 5H), 7.86 (dd, J = 6.15, 2.91 Hz, 1H), 7.93 (s, 2H). ^{13}C NMR (100 MHz, CD_3Cl_3): δ ppm 12.8, 14.4, 20.8, 23.1, 34.7, 44.5, 47.8, 49.3, 53.7, 60.6, 62.2, 65.2, 68.7, 72.7, 74.8, 85.6, 97.7, 105.3, 108.1, 122.9, 123.1, 123.2, 124.1, 124.5, 128.5, 129.2, 131.1, 132.9, 144.5, 144.8, 145.5, 148.9, 149.0, 153.4, 153.5, 153.6, 167.8, 169.6, 170.6, 170.9, 171.0. Compound **15** was dissolved in anhydrous MeOH and NaOMe (10% by weight) was added. After stirring overnight, the reaction mixture was neutralized with Dowex 50WX8 proton exchange resin and filtered. This crude product was further purified using fluorisil. LysoProbe VI was obtained as a light yellow solid in 42% yield. ^{1}H NMR (400 MHz, CD_3OD): δ ppm 1.13 (t, J = 7.05 Hz, 12H), 1.71 (s, 6H), 1.87 (s, 2H), 3.34 (m, 8H), 3.66 (m, 12H), 3.89 (d, J = 12.01, 2H), 4.26 (t, J = 10.01 Hz, 2H), 4.31 (d, J = 2.46 Hz, 2H), 4.64 (t, J = 5.6 Hz, 2H), 4.75 (t, J=5.6 Hz, 4H), 5.77 (d, J = 9.78 Hz, 2H), 6.21-6.25 (m, 4H), 6.36 (t, J = 2.35 Hz, 2H), 7.01-7.06 (m, 2H), 7.51 (ddd, J = 6.08, 4.29, 1.50 Hz, 2H), 7.77 (s, 1H), 7.87 (dd, J = 6.46, 2.90 Hz, 1H), 8.15 (s, 1H). HR/MS $[M+H]^+$ Calcd: 1467.6367. Found: 1467.6388.

Rhodamine-Lactose I: To a solution of compound **2** (0.839 mmol) in DMSO: t-BuOH: H_2O (8:4:7), $CuSO_4$, Na ascorbate and TBTA (0.08 mmol) were added sequentially. Then 1-azido-1-deoxy-β-D-lactopyranoside (**17**, 0.700 mmol) was added with continuous stirring overnight. The reaction mixture

pH was adjusted to neutrality and extracted with CH_2Cl_2. The combined organic layers were dried over Na_2SO_4 and evaporated. The crude product was purified on fluorisil using MeOH:CH_2Cl_2 (1:20-1:10). The title compound was obtained as a colorless syrup in 74% yield. ^{1}H NMR (400 MHz, CD_3OD) δ ppm 1.14 (dt, J = 6.96, 2.10 Hz, 12H), 3.36 (m, 8H), 3.66 (m, 12H), 4.36 (m, 3H), 5.38 (d, J = 8.82 Hz, 1H), 6.27 (m, 4H), 6.40 (m, 2H), 7.05 (dd, J = 6.46, 2.12 Hz, 1H), 7.28 (s, 1H), 7.50 (m, 2H), 7.86 (dd, J = 6.11, 1.91 Hz, 1H). HR/MS $[M+H]^+$ Calcd: 846.3800; Found: 847.3779.

Rhodamine-di-Lactose II: To a solution of compound **6** (0.439 mmol) in DMSO: t-BuOH: H_2O (8:4:7), $CuSO_4$, Na ascorbate and TBTA (0.05 mmol) were added. Then 1-azido-1-deoxy-β-D-lactopyranoside (**17**, 0.929 mmol) was added with overnight stirring. The reaction mixture pH was adjusted to neutrality and extracted with CH_2Cl_2. The combined organic layers were dried over Na_2SO_4 and evaporated. The crude product was purified on fluorisil using MeOH:DCM (1:20-1:10-50:50). The title compound was obtained as a syrup in 25% yield. ^{1}H NMR (400 MHz, DMSO) δ ppm 1.03 (t, J = 6.90 Hz, 12H), 3.55 (m, 24H), 3.75 (d, J = 10.63 Hz, 2H), 3.84 (t, J = 9.07 Hz, 2H), 4.18 (s, 2H), 4.23 (d, J = 7.01 Hz, 2H), 4.62 (td, J = 14.63, 6.73 Hz, 4H), 5.60 (d, J = 8.56 Hz, 2H), 6.17 (td, J = 21.88, 5.55 Hz, 4H), 6.27 (d, J = 2.19 Hz, 2H), 6.97 (m, 1H), 7.32 (s, 1H), 7.47 (dd, J = 5.71, 3.09 Hz, 2H), 7.78 (dd, J = 6.14, 2.88 Hz, 1H), 7.91 (s, 1H), 8.19 (s, 2H).

Absorbance and Fluorescence Spectroscopy: Probes were dissolved in DMSO and diluted with water to prepare stock solutions. After dilution with different pH buffers to the final concentration of measurement, UV-vis spectra were recorded using a Perkin Elmer Lambda 35 UV/Vis Spectrometer equipped with PTP 1+1 Peltier Temperature Programmer at room temperature. The slit width was 4 nm. A 10 × 10 mm quartz cell was used for all absorbance measurements. Final concentration for absorbance was 10 μM. Fluorescence spectra were obtained by using a Horiba Jobin Yvon Fluoromax-4 spectrofluorometer. The slit width was 5 nm for both excitation and emission. All samples were excited at 480 nm. A 10 × 10 mm quartz cell was used for all fluorescence measurements. Final concentration of probe was 1 μM.

Quantum Yield: The slit width was 5 nm for both excitation and emission. All the samples were excited at 540 nm. A 10x10 mm quartz cell was used for all fluorescence measurements. Relative quantum yields of LysoProbes I-VI were compared to Rhodamine B. The following equation was used to determine relative quantum yields:

ϕF = (A standard/A sample) (F sample/F standard)(n sample/n standard)2 ϕF*standard (Eq. 1)

ϕF* (Rhodamine B) = 0.69 in EtOH

F is the area under the emission curve of probes and A is the absorbance value at the excitation wavelength. The same solution was utilized during the experiments for probes and RhB. Accordingly, the refractive index ratio was eliminated from equation 1, with a value equivalent to 1.

Table S1. Quantum yields of LysoProbes I-VII measured at pH4.6 (citric acid-phosphate buffer).

LysoProbe I	0.16
LysoProbe II	0.53
LysoProbe III	0.64
LysoProbe IV	0.52
LysoProbe V	0.89
LysoProbe VI	0.93

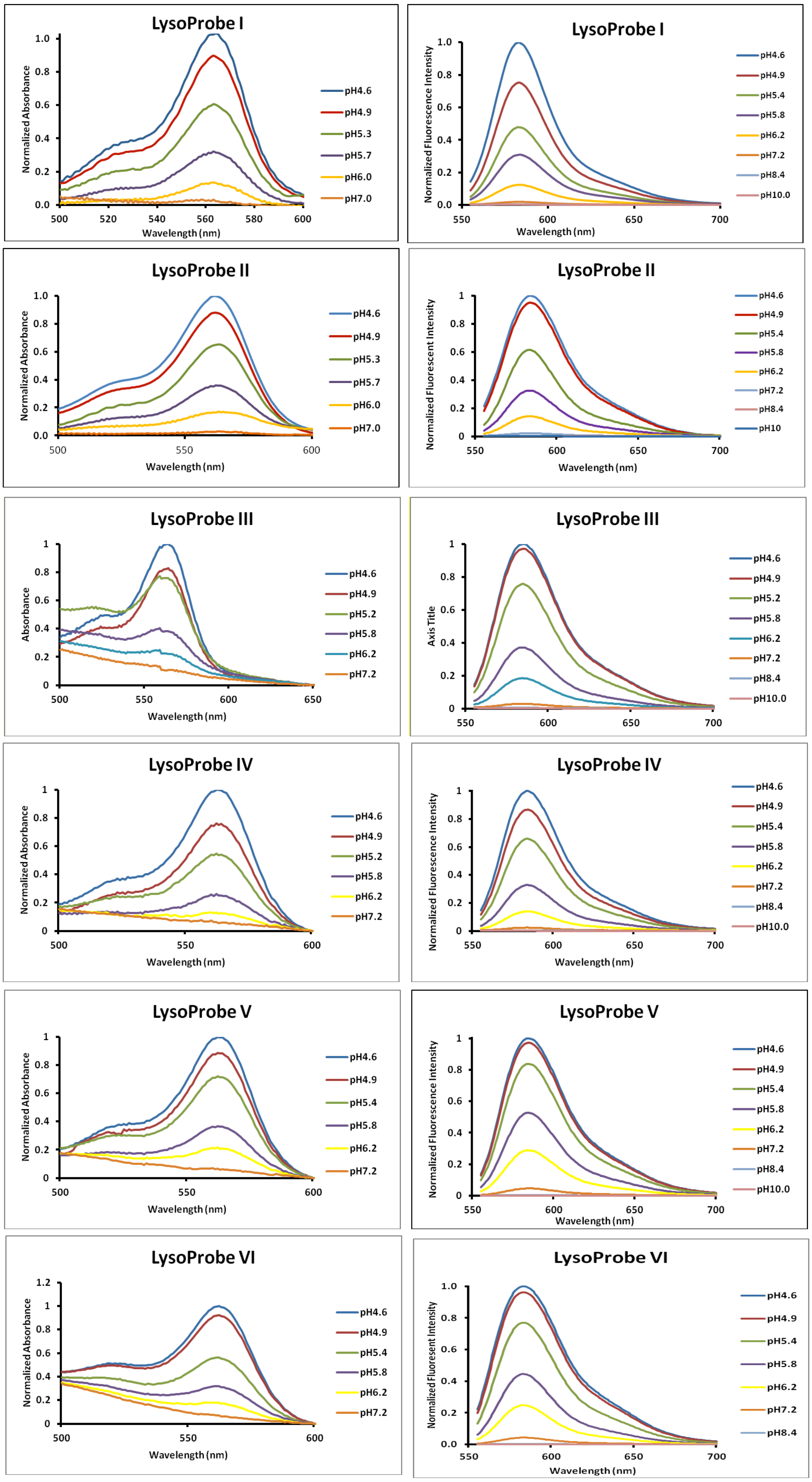

Figure S10. Absorbance and fluorescence spectra of LysoProbes I-VI.

NMR Spectra:

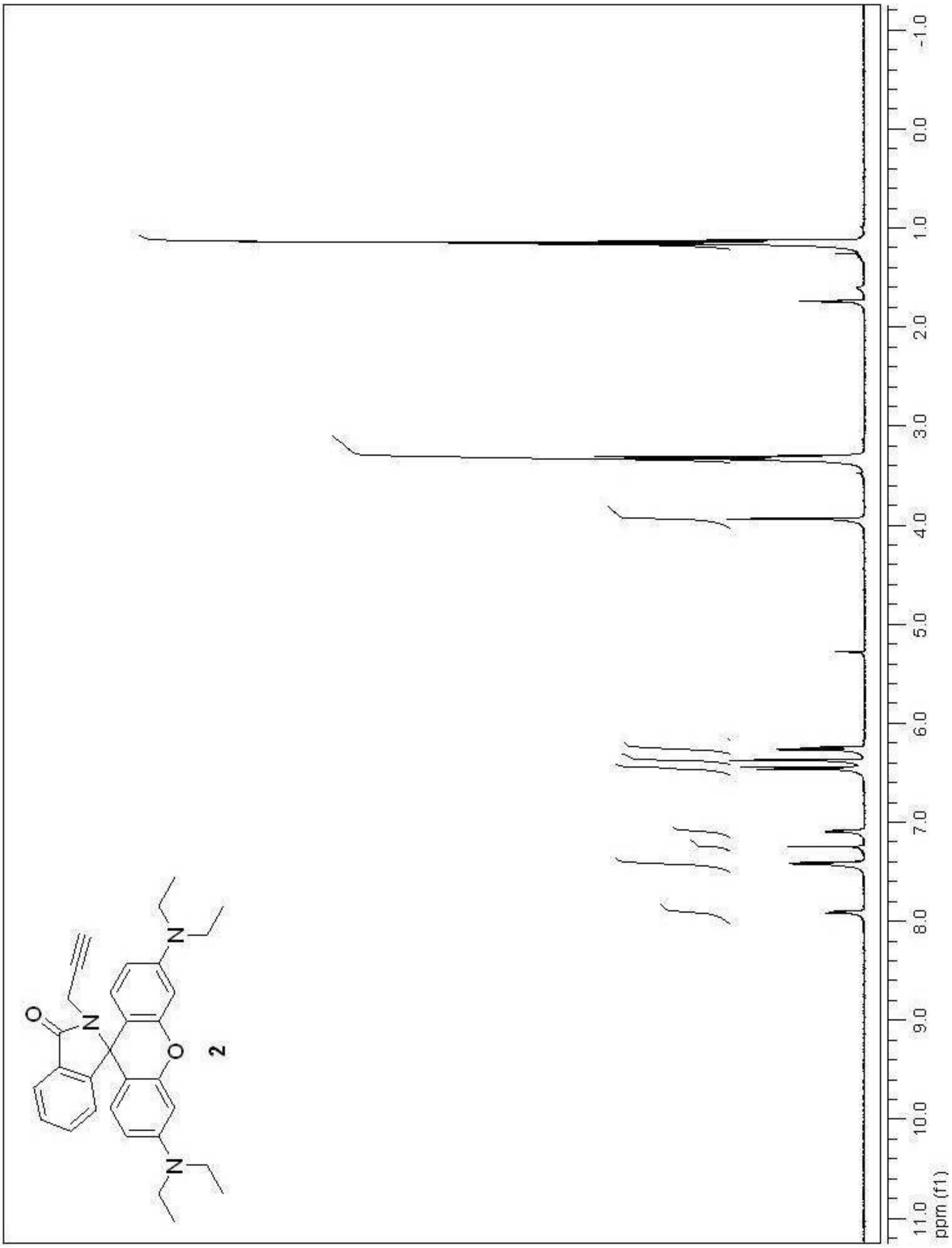

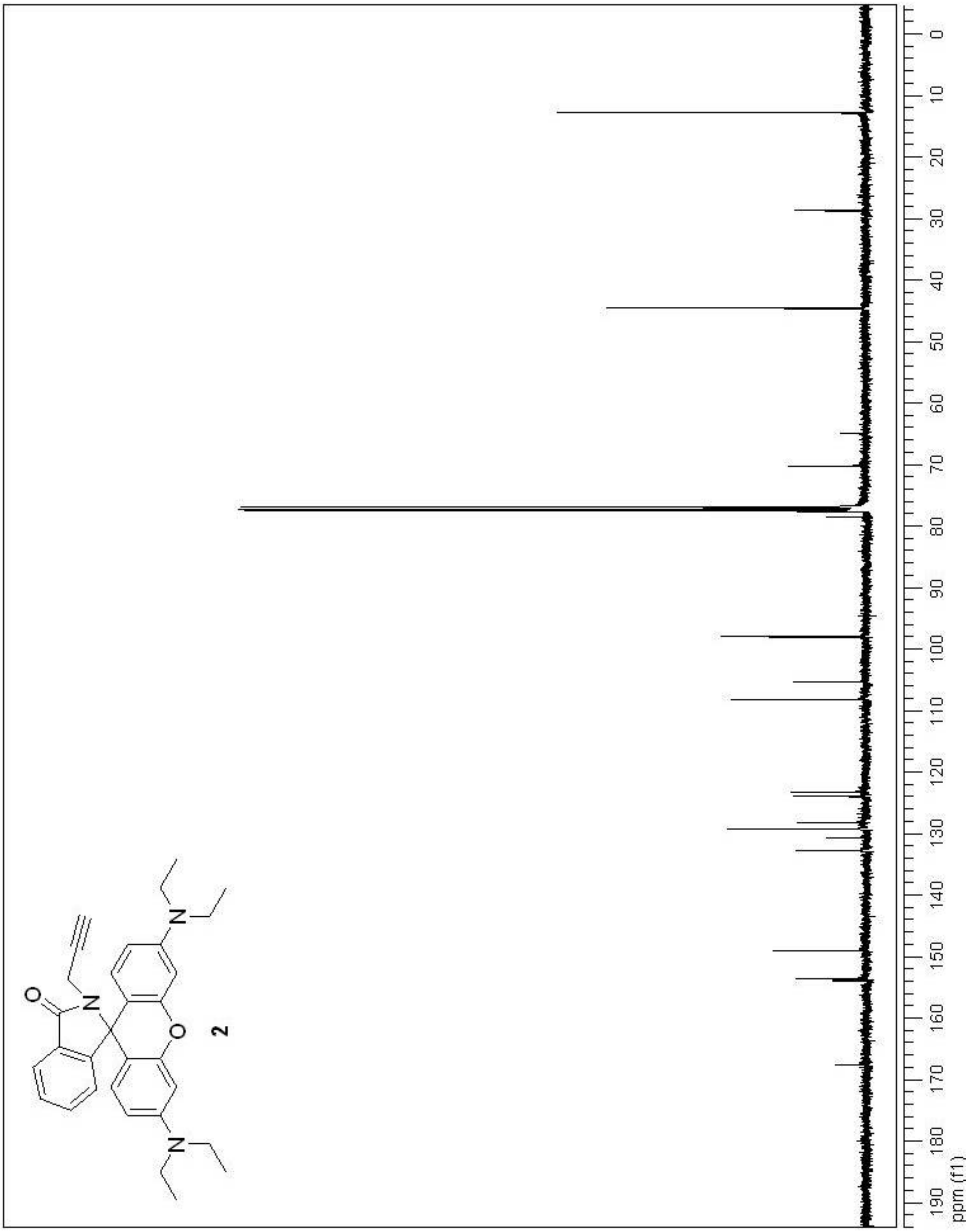

2
ppm (f1)
0
10
20
30
40
50
60
70
80
90
100
110
120
130
140
150
160
170
180
190

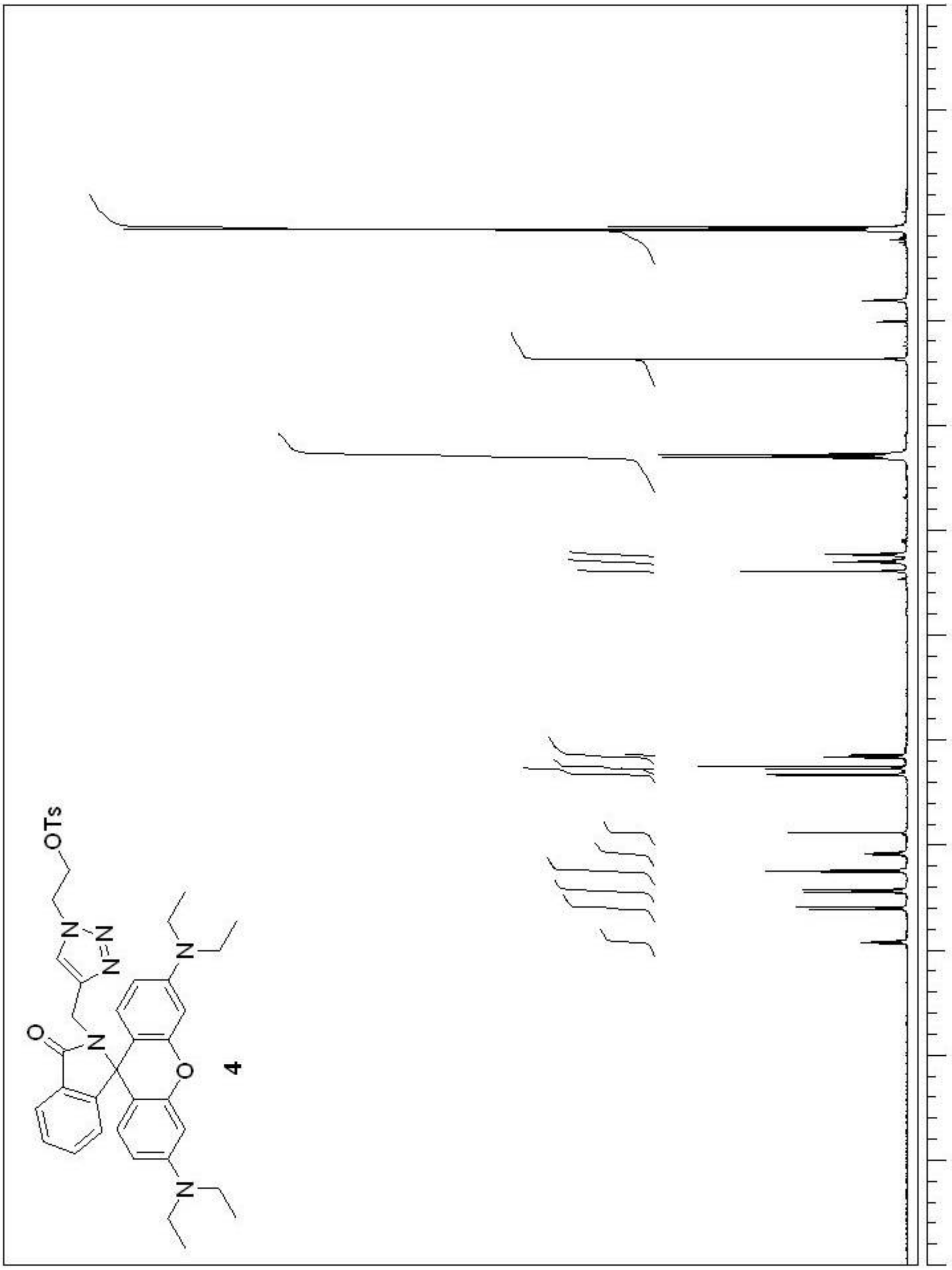

OTs
4
ppm (t1)
10.0
9.0
8.0
7.0
6.0
5.0
4.0
3.0
2.0
1.0
0.0

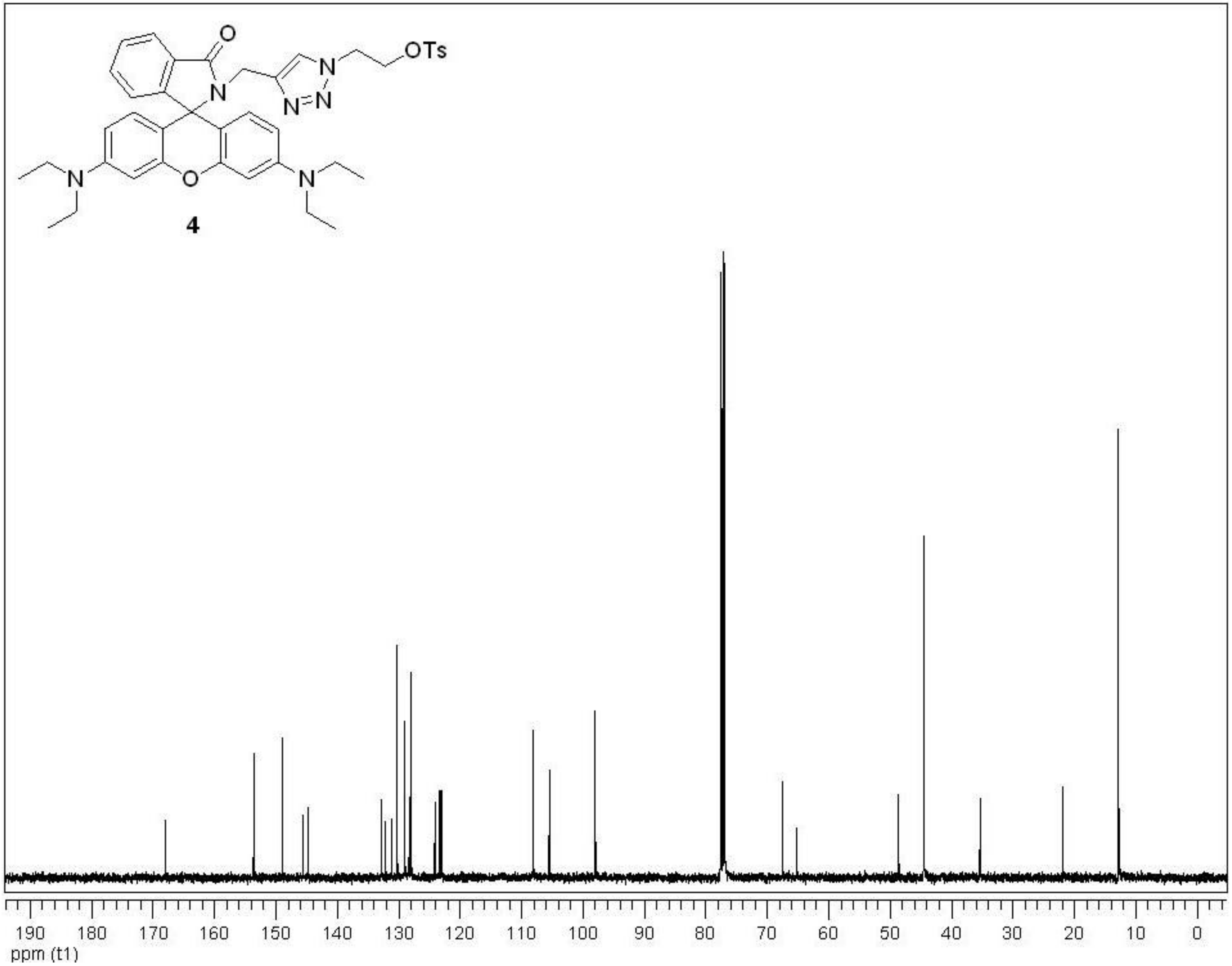
OTs
190 180 170 160 150 140 130 120 110 100 90 80 70 60 50 40 30 20 10 0
ppm (t1)

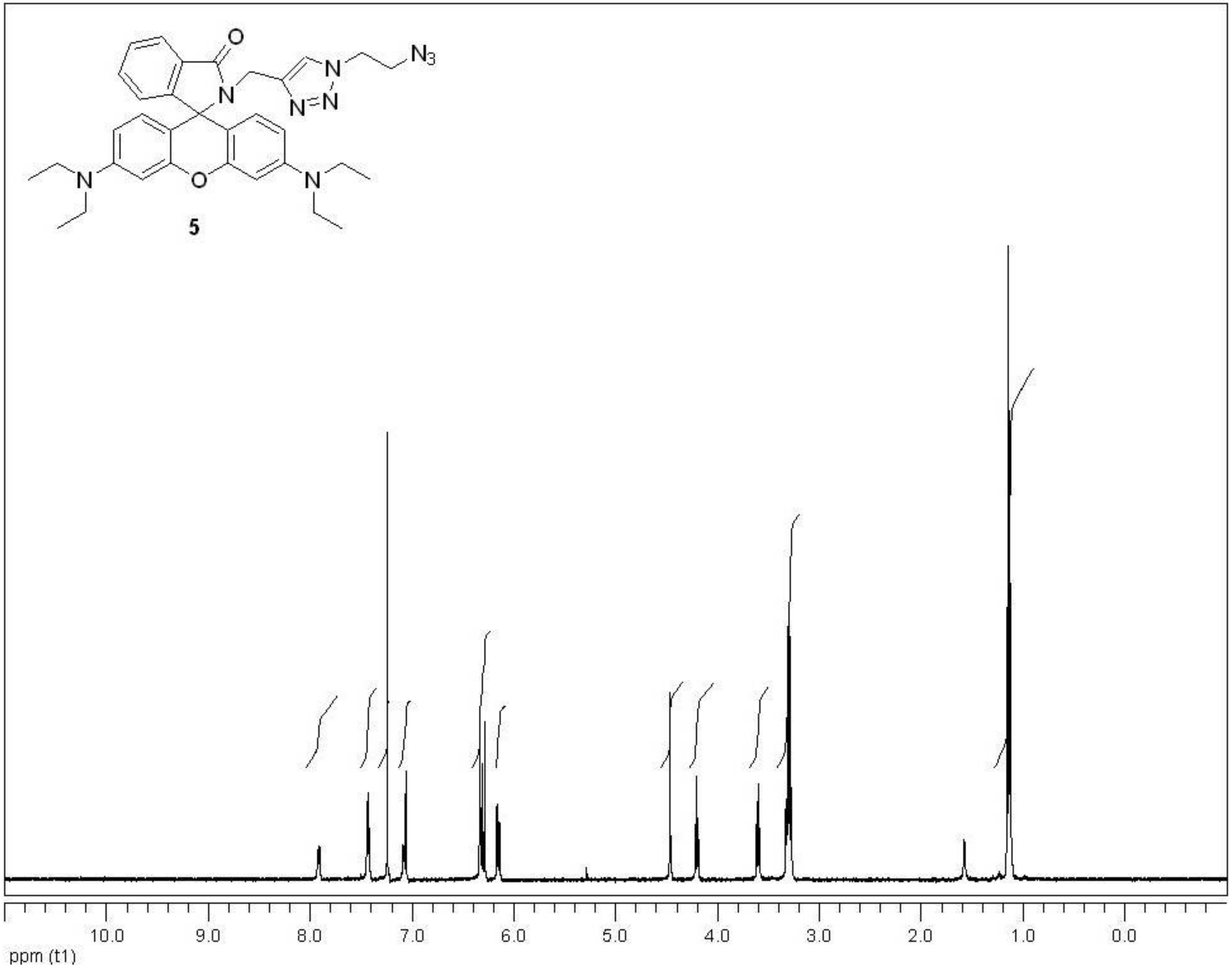

5
ppm (t1)
10.0
9.0
8.0
7.0
6.0
5.0
4.0
3.0
2.0
1.0
0.0

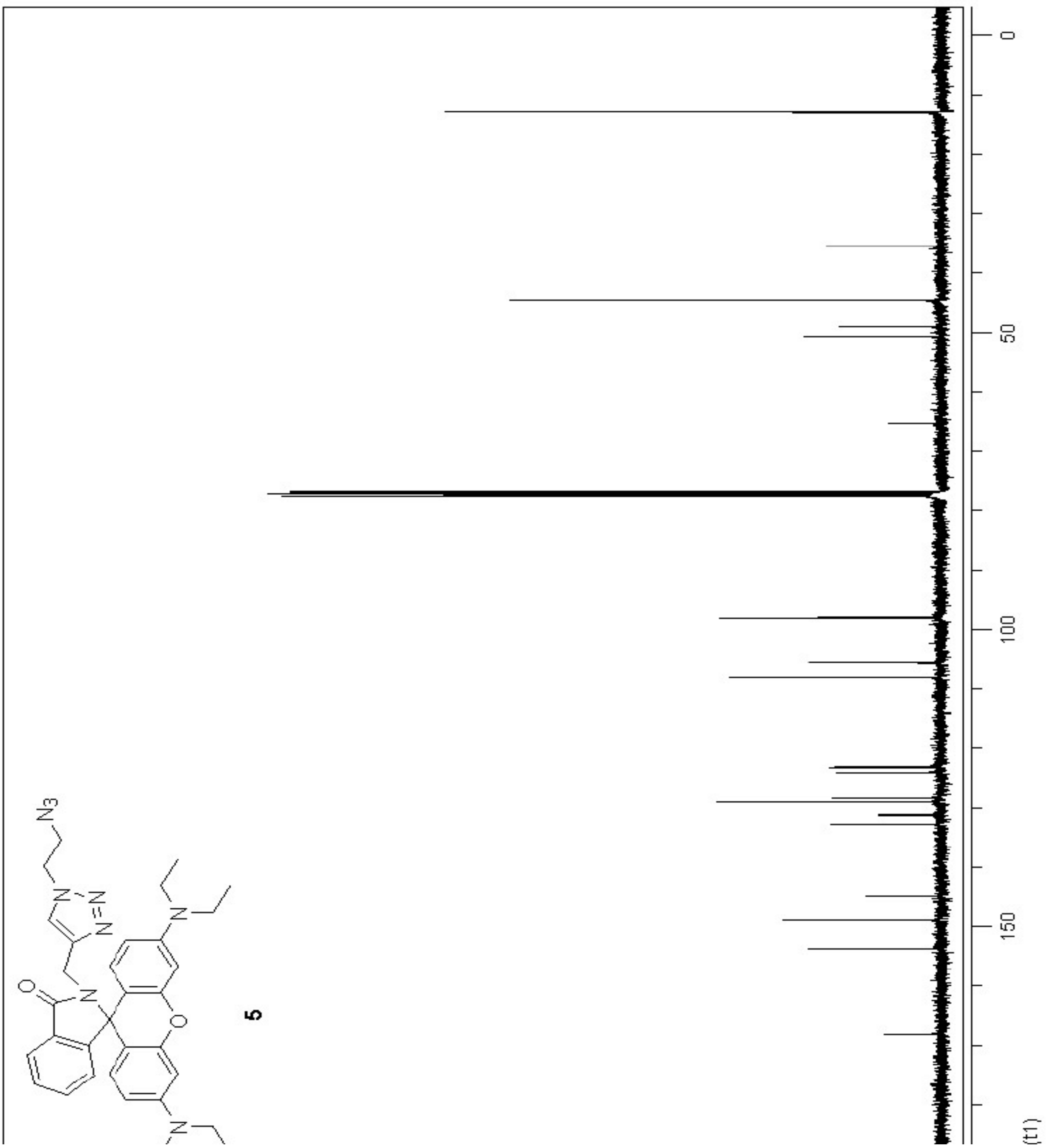
N3
5
0
50
100
150
(t1)

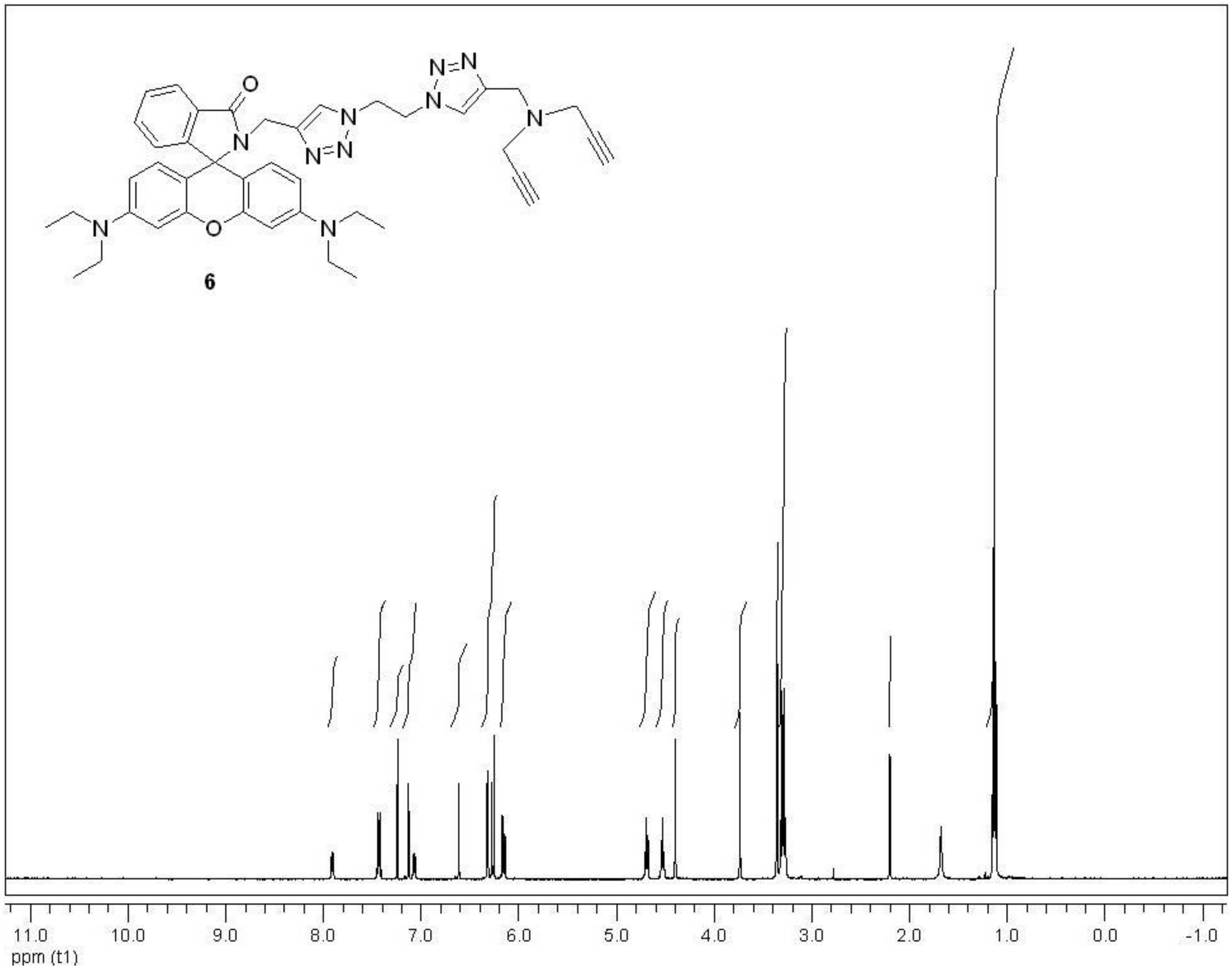
6
11.0 10.0 9.0 8.0 7.0 6.0 5.0 4.0 3.0 2.0 1.0 0.0 -1.0
ppm (t1)

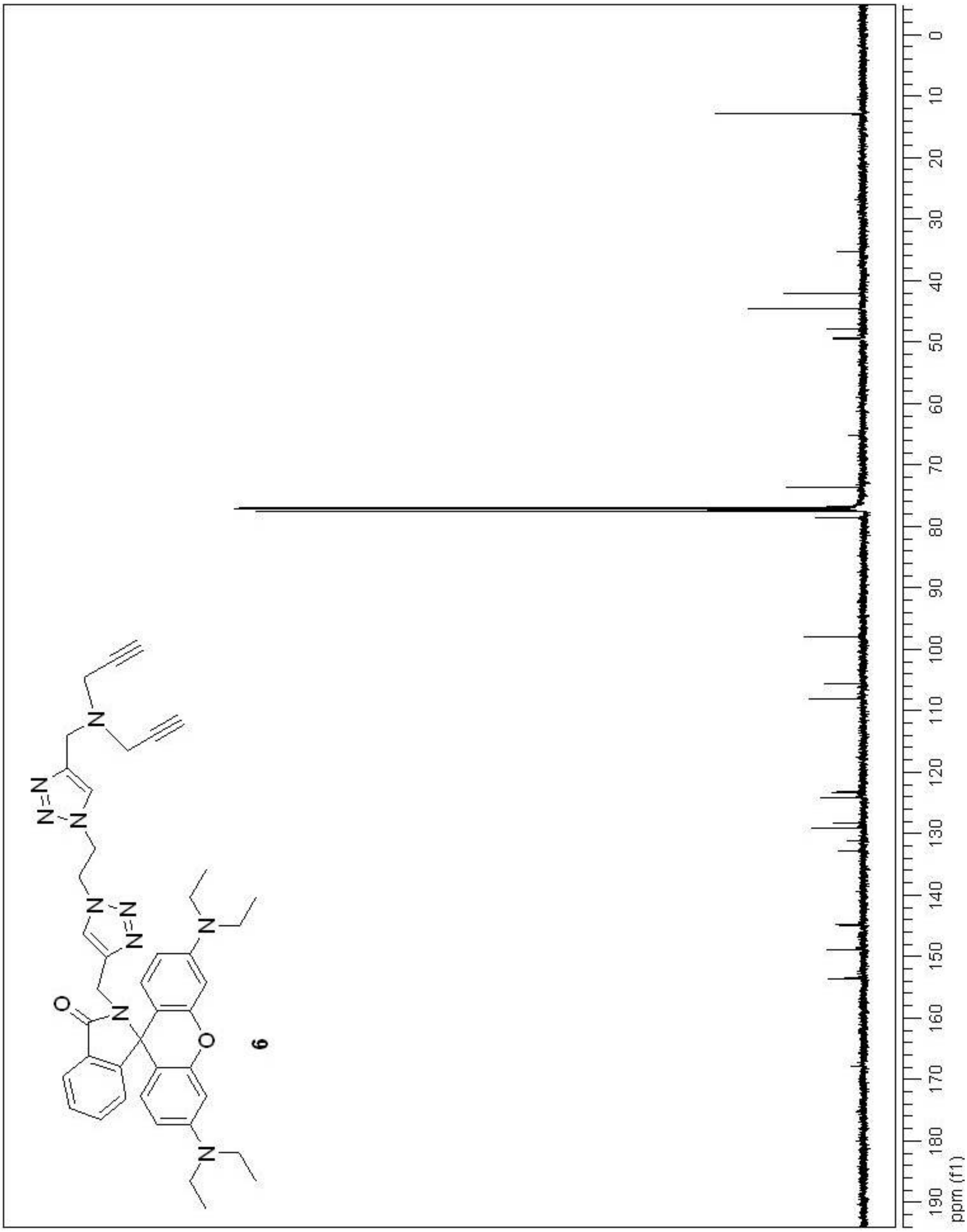
6
ppm (f1)
0 10 20 30 40 50 60 70 80 90 100 110 120 130 140 150 160 170 180 190

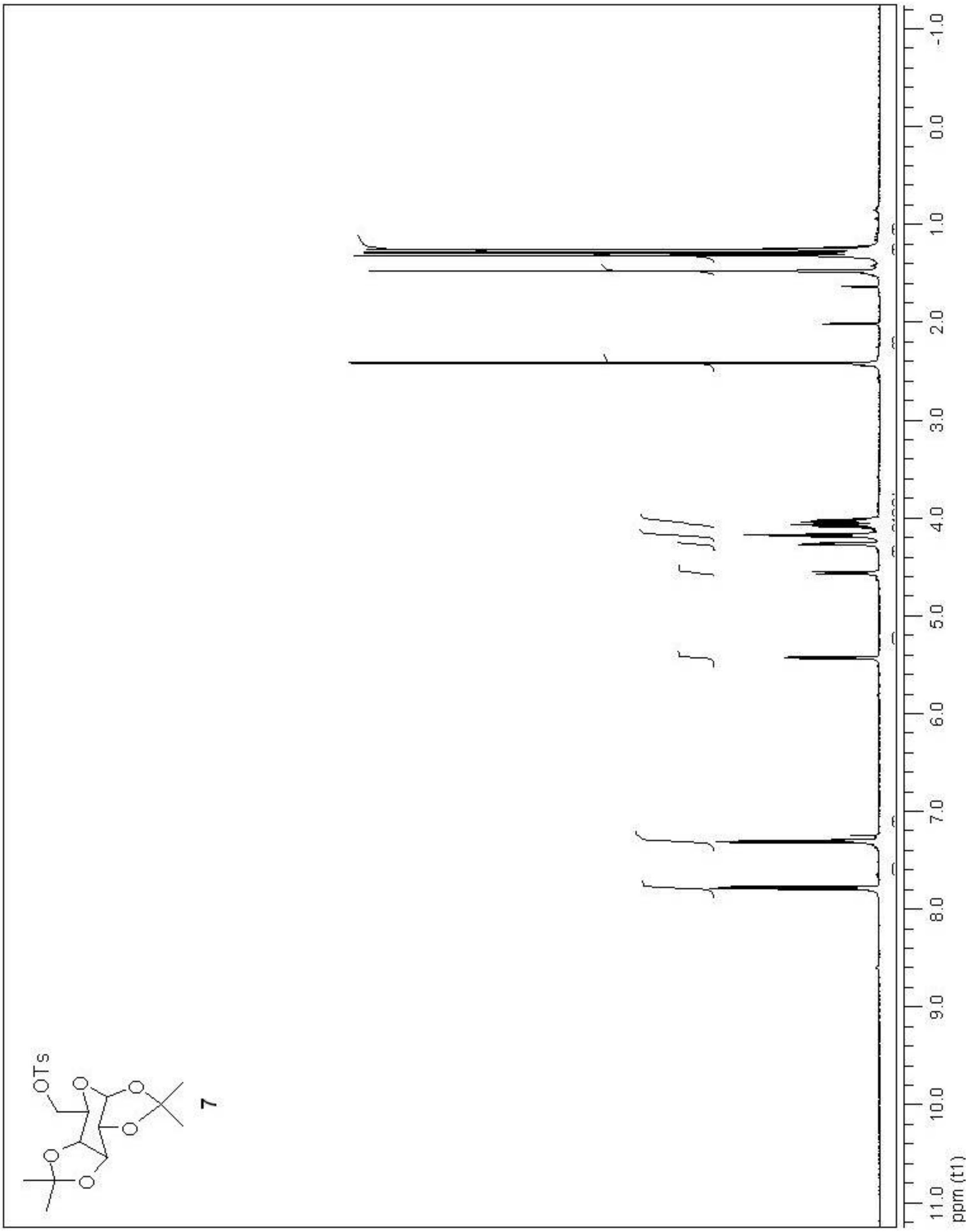
OTs
7
-1.0
0.0
1.0
2.0
3.0
4.0
5.0
6.0
7.0
8.0
9.0
10.0
11.0
ppm (t1)

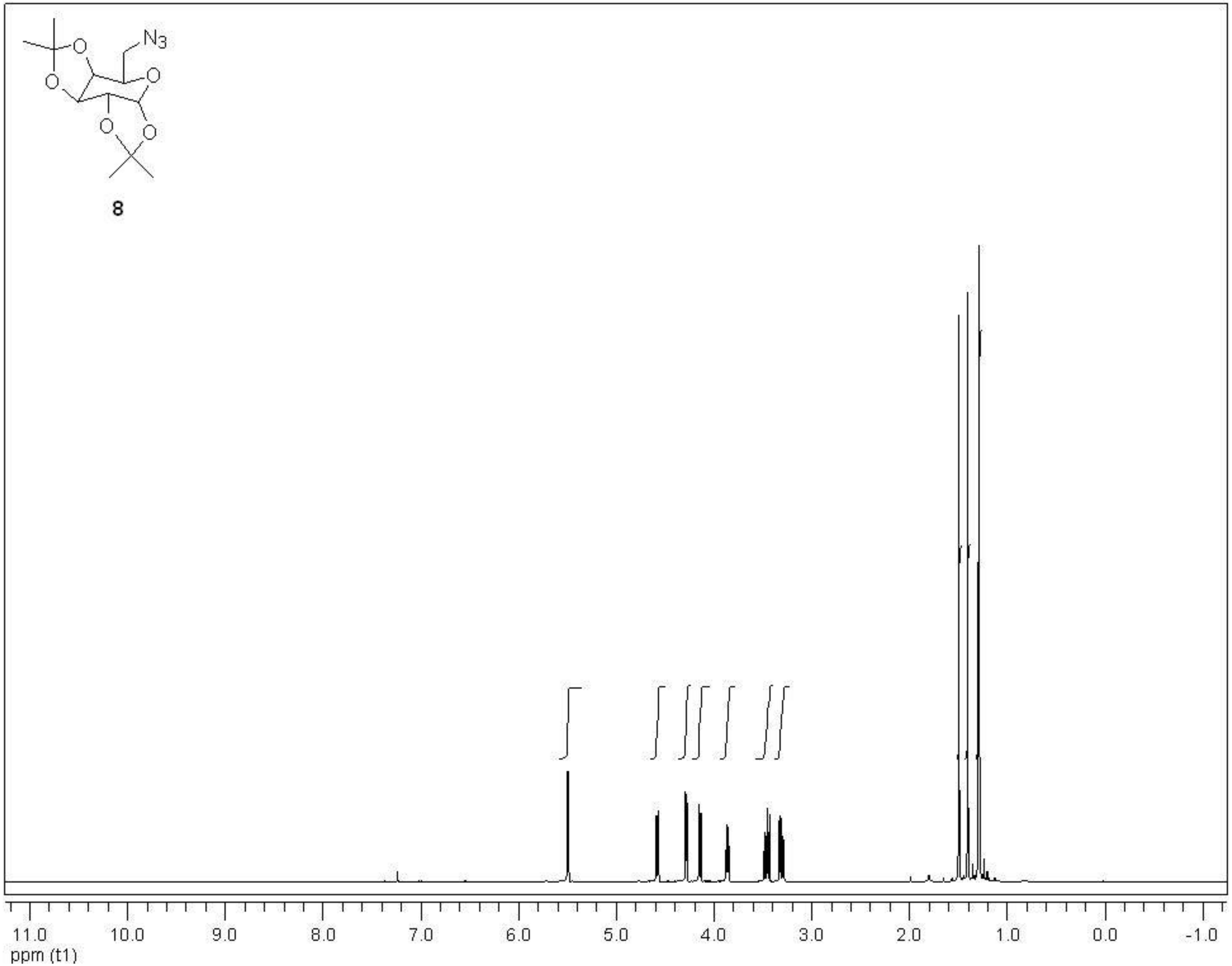
N3
8
11.0
10.0
9.0
8.0
7.0
6.0
5.0
4.0
3.0
2.0
1.0
0.0
-1.0
ppm (t1)

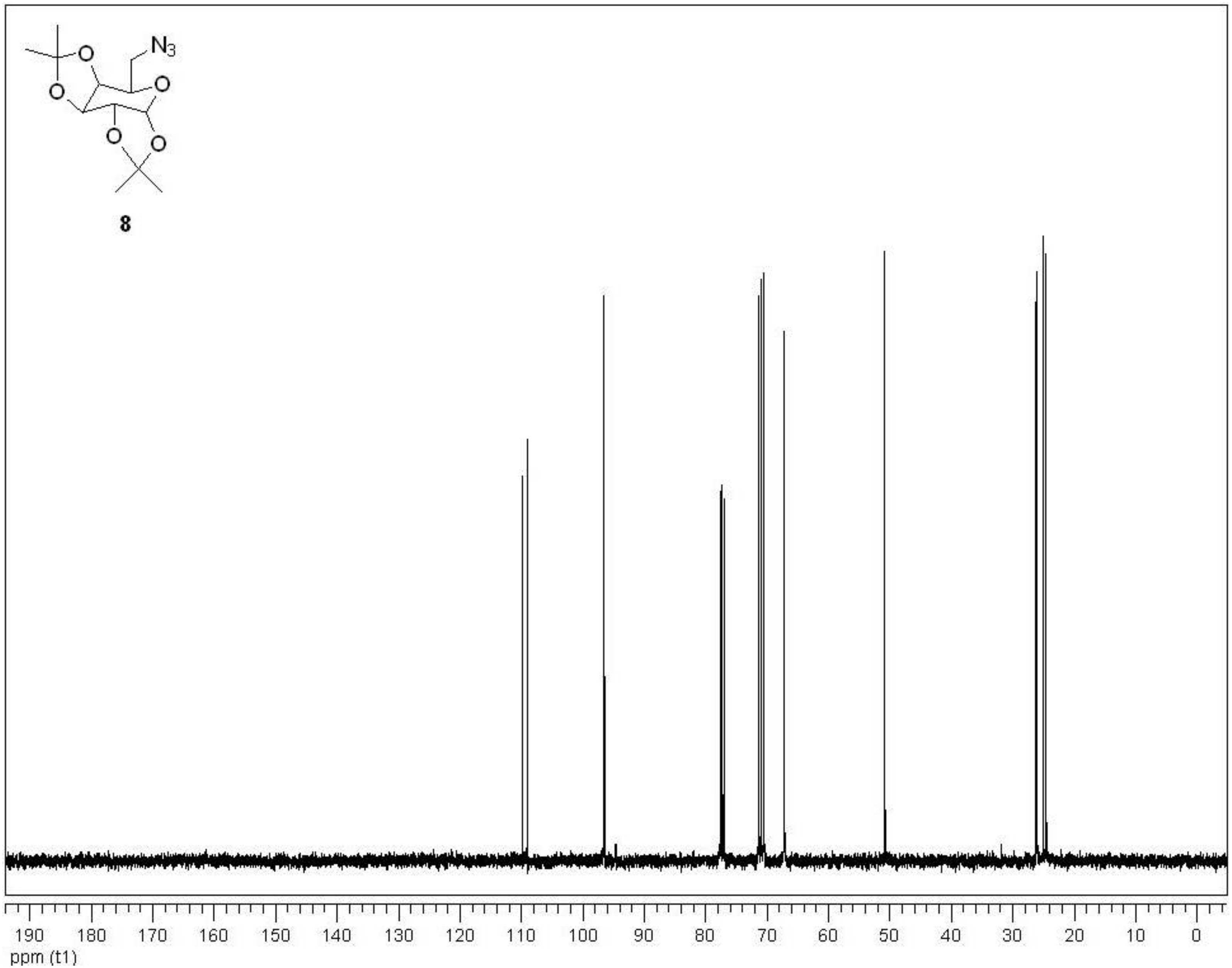
N3
8
190 180 170 160 150 140 130 120 110 100 90 80 70 60 50 40 30 20 10 0
ppm (t1)

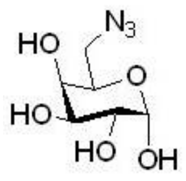
HO
N3
O
HO
HO
OH

9

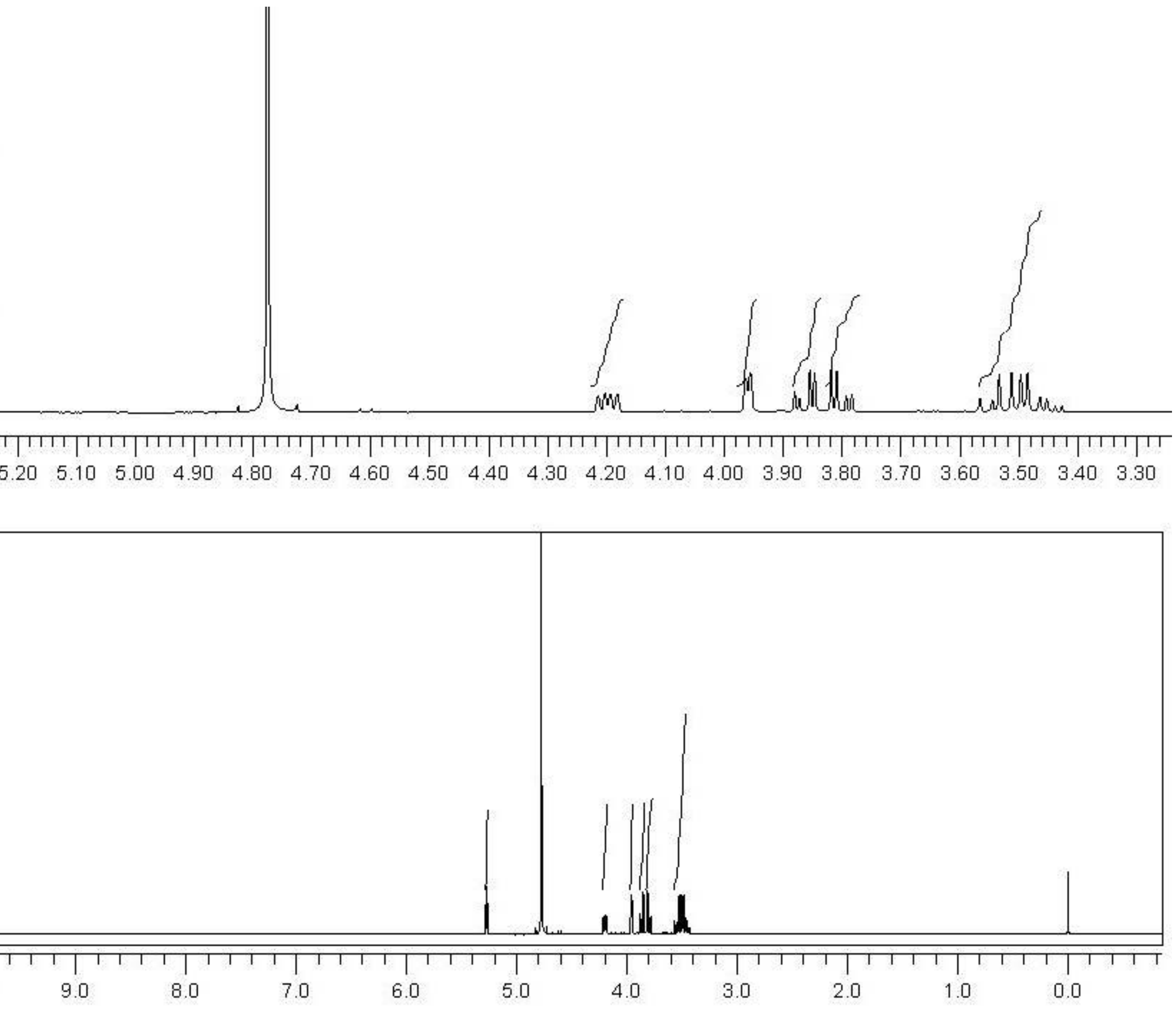
5.40 5.30 5.20 5.10 5.00 4.90 4.80 4.70 4.60 4.50 4.40 4.30 4.20 4.10 4.00 3.90 3.80 3.70 3.60 3.50 3.40 3.30
ppm (f1)
10.0 9.0 8.0 7.0 6.0 5.0 4.0 3.0 2.0 1.0 0.0
ppm (f1)

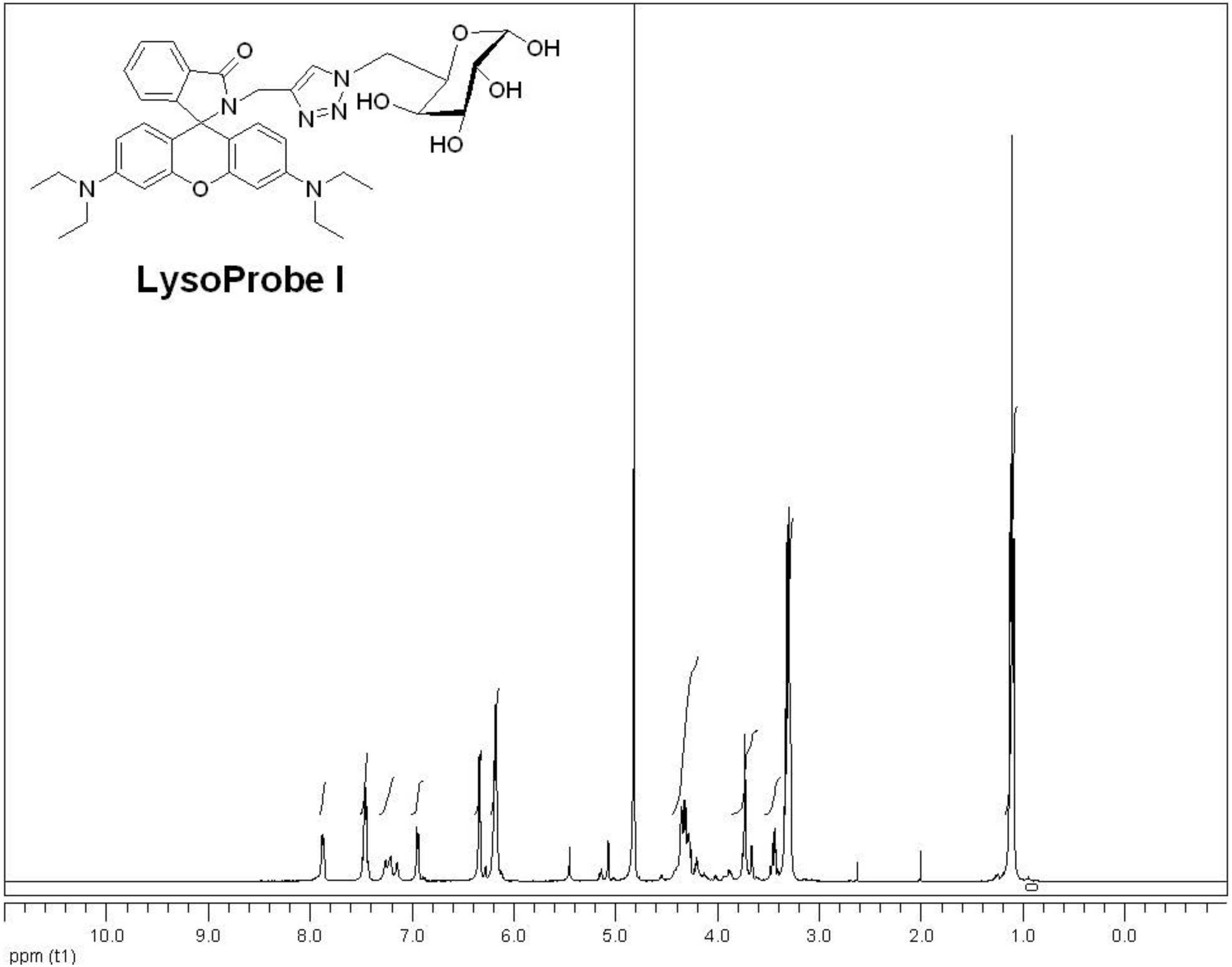

LysoProbe I

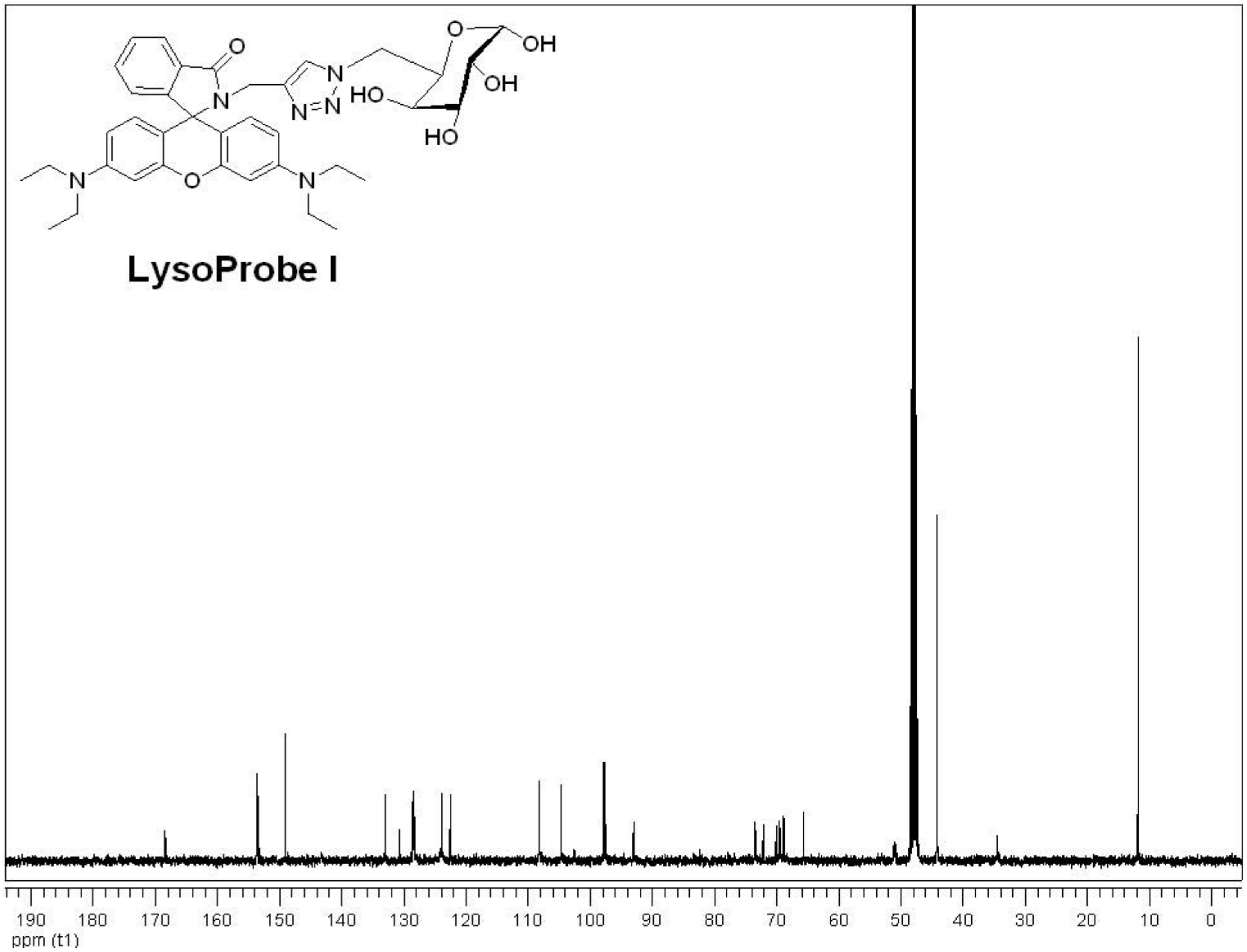
LysoProbe I
OH
OH
HO
HO
190 180 170 160 150 140 130 120 110 100 90 80 70 60 50 40 30 20 10 0
ppm (t1)

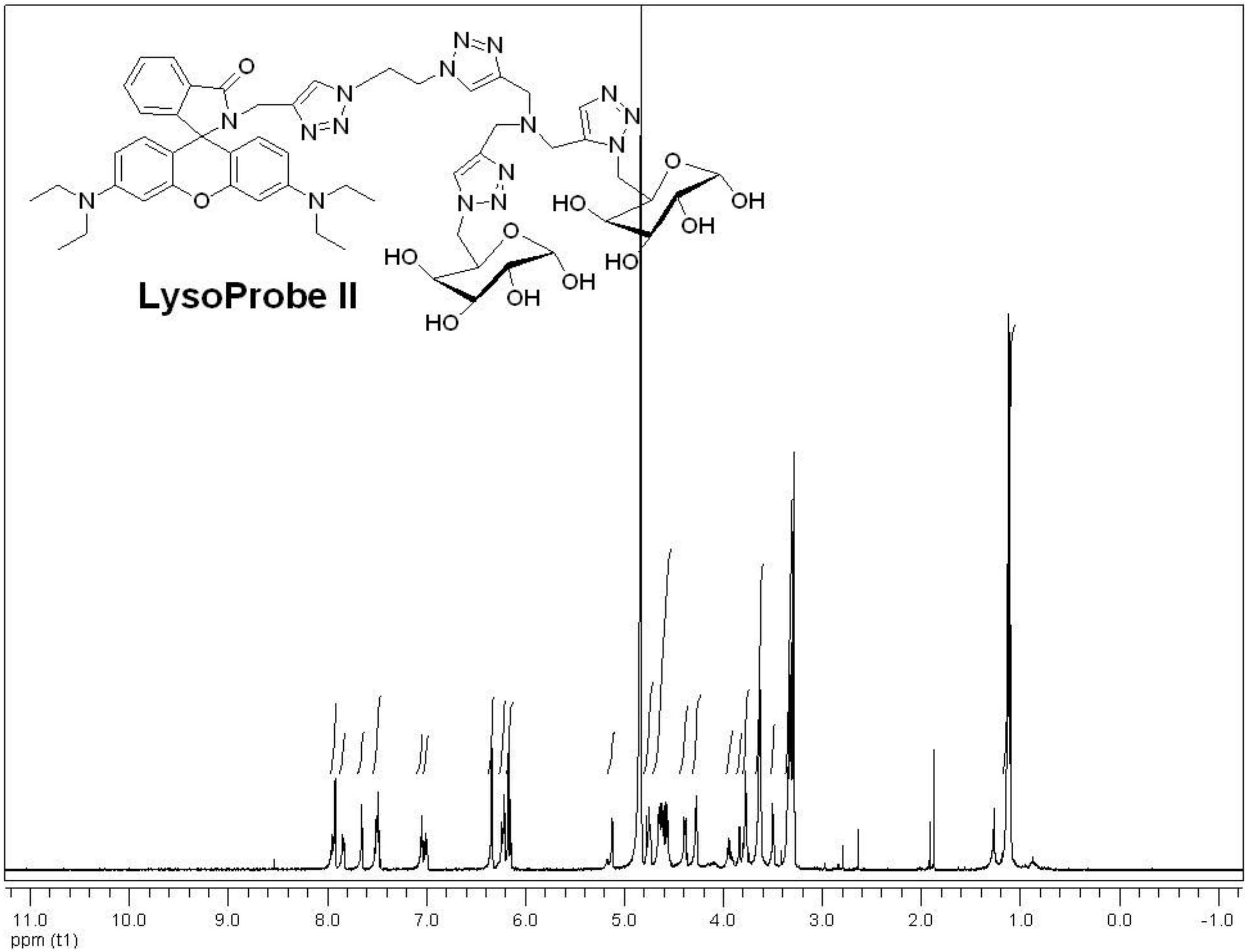

LysoProbe II
11.0
10.0
9.0
8.0
7.0
6.0
5.0
4.0
3.0
2.0
1.0
0.0
-1.0
ppm (t1)

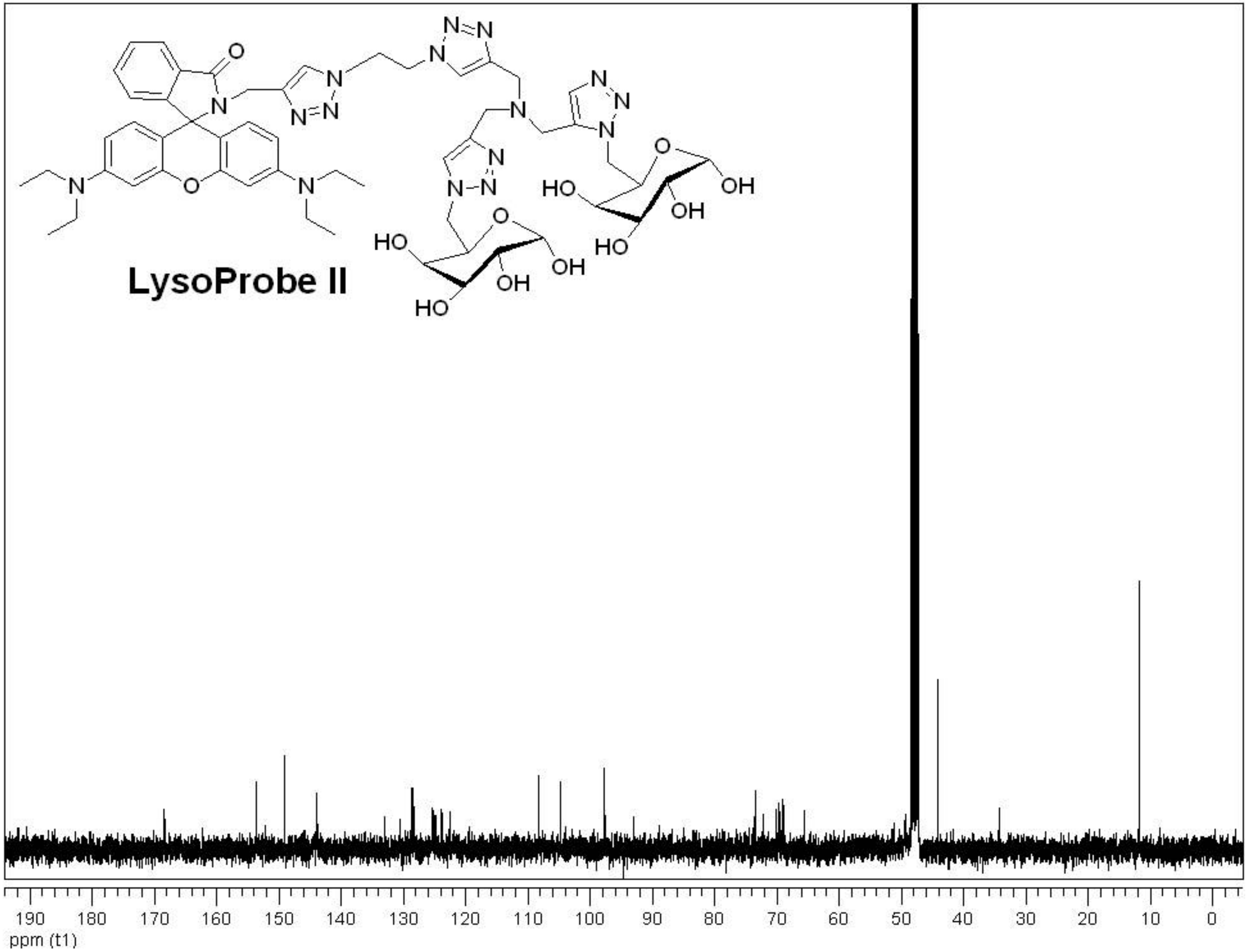
LysoProbe II
190 180 170 160 150 140 130 120 110 100 90 80 70 60 50 40 30 20 10 0
ppm (t1)

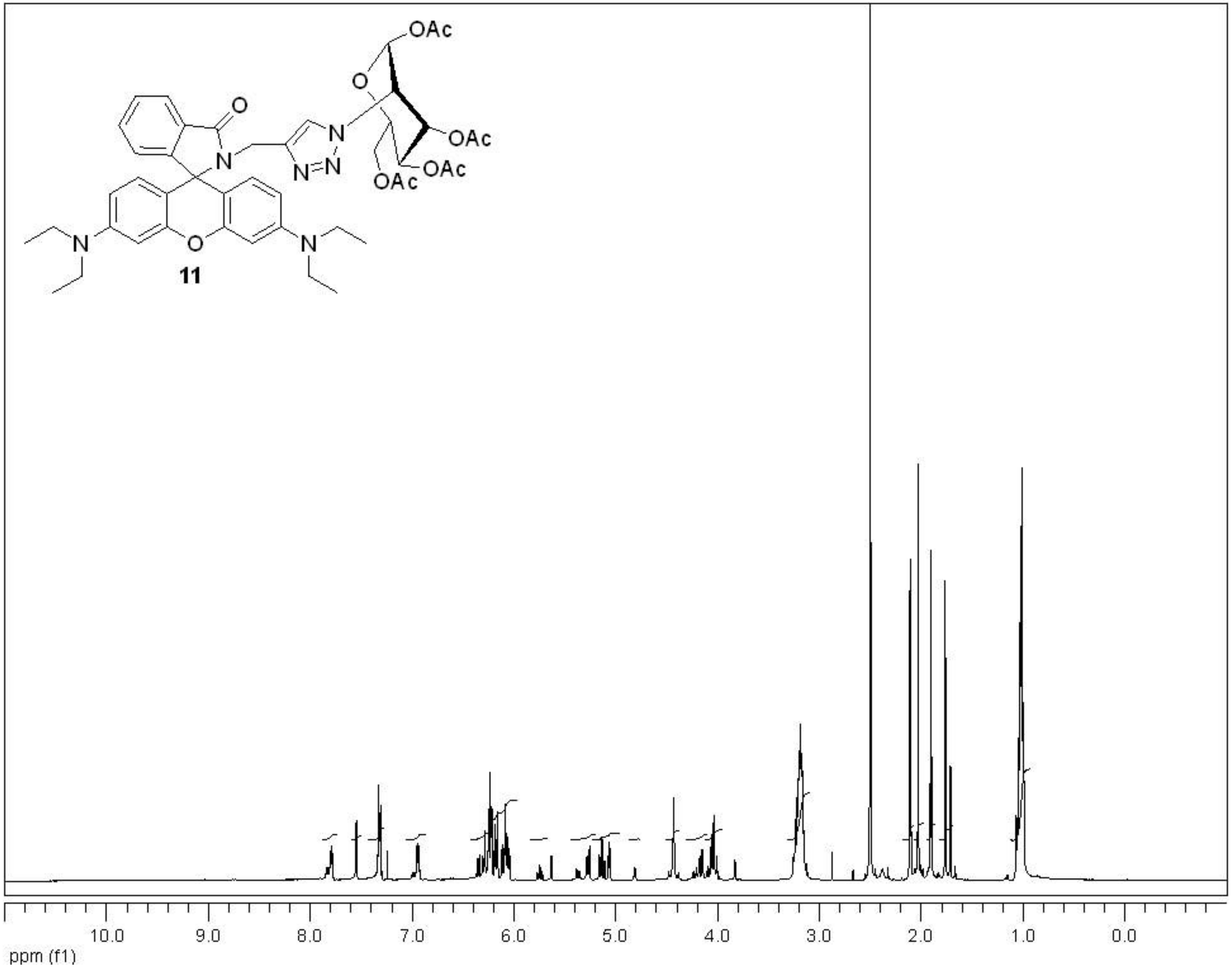

OAc
OAc
OAc
OAc
O
N
N
N=N
O
N
N
O
11
10.0
9.0
8.0
7.0
6.0
5.0
4.0
3.0
2.0
1.0
0.0
ppm (f1)

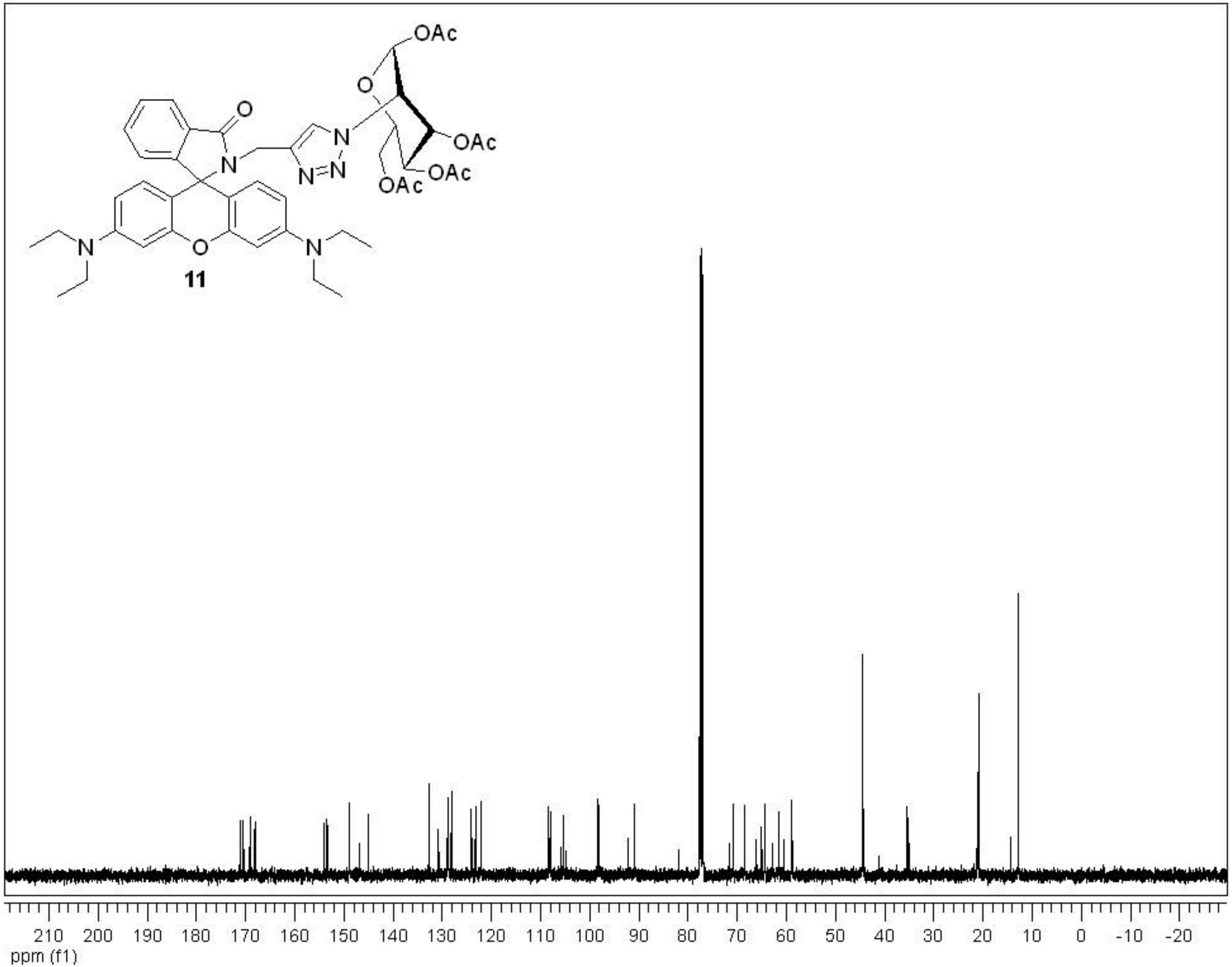
OAc
OAc
OAc
OAc
OAc
11
210 200 190 180 170 160 150 140 130 120 110 100 90 80 70 60 50 40 30 20 10 0 -10 -20
ppm (f1)

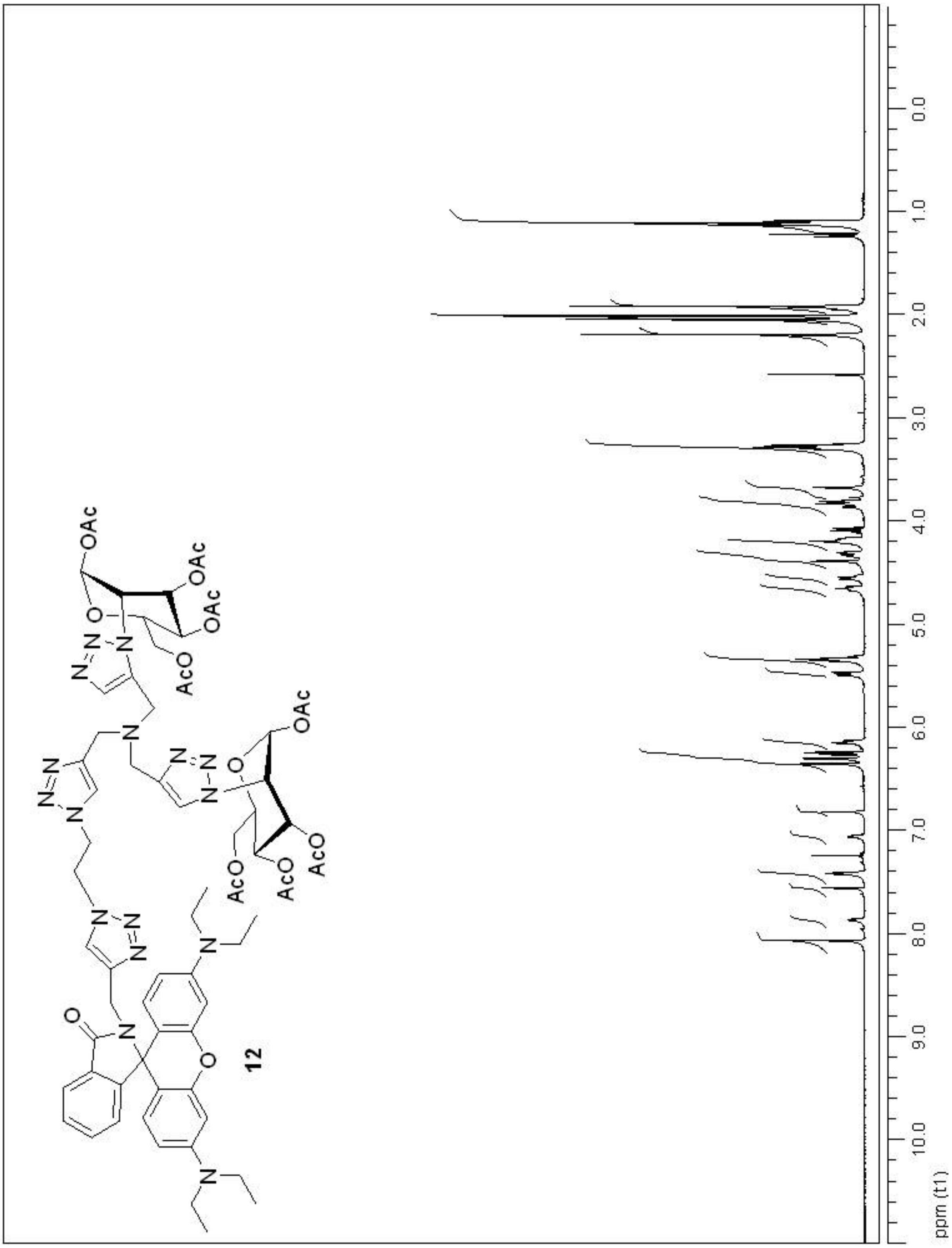
12
ppm (t1)
0.0
1.0
2.0
3.0
4.0
5.0
6.0
7.0
8.0
9.0
10.0
OAc
AcO

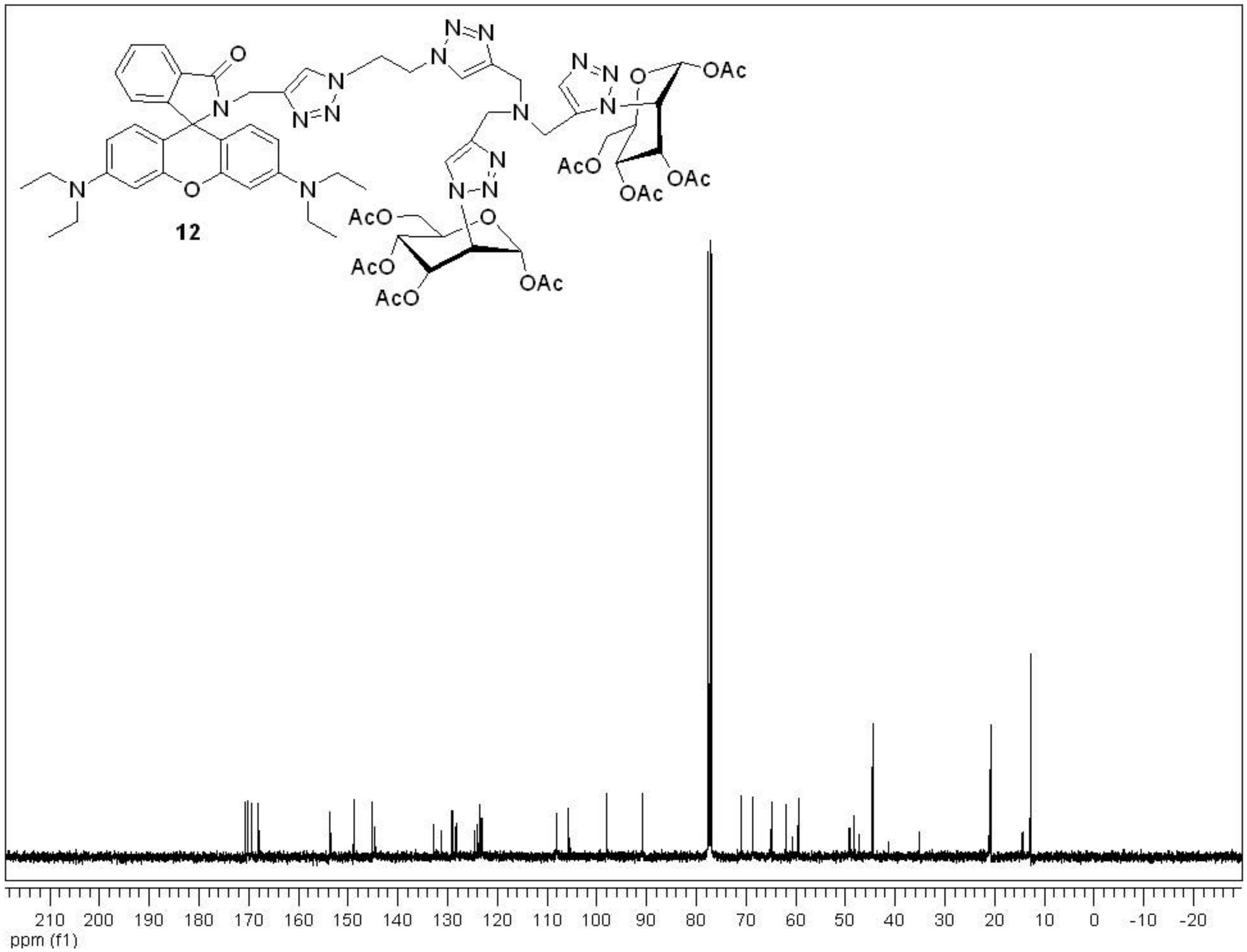
12
OAc
AcO
210 200 190 180 170 160 150 140 130 120 110 100 90 80 70 60 50 40 30 20 10 0 -10 -20
ppm (f1)

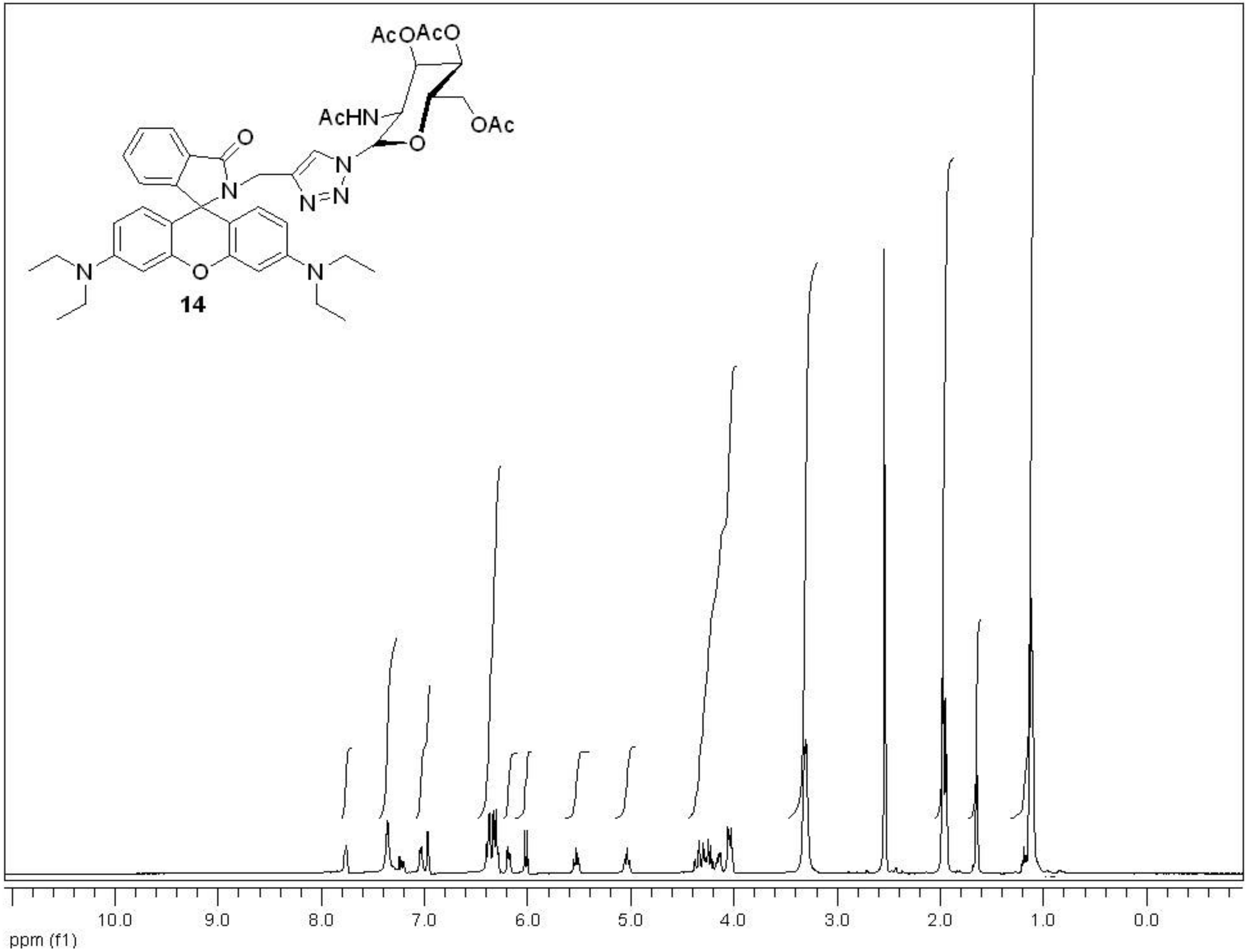
AcO AcO
AcHN
OAc
O
N
N
N
N
O
N
N
O
14
10.0
9.0
8.0
7.0
6.0
5.0
4.0
3.0
2.0
1.0
0.0
ppm (f1)

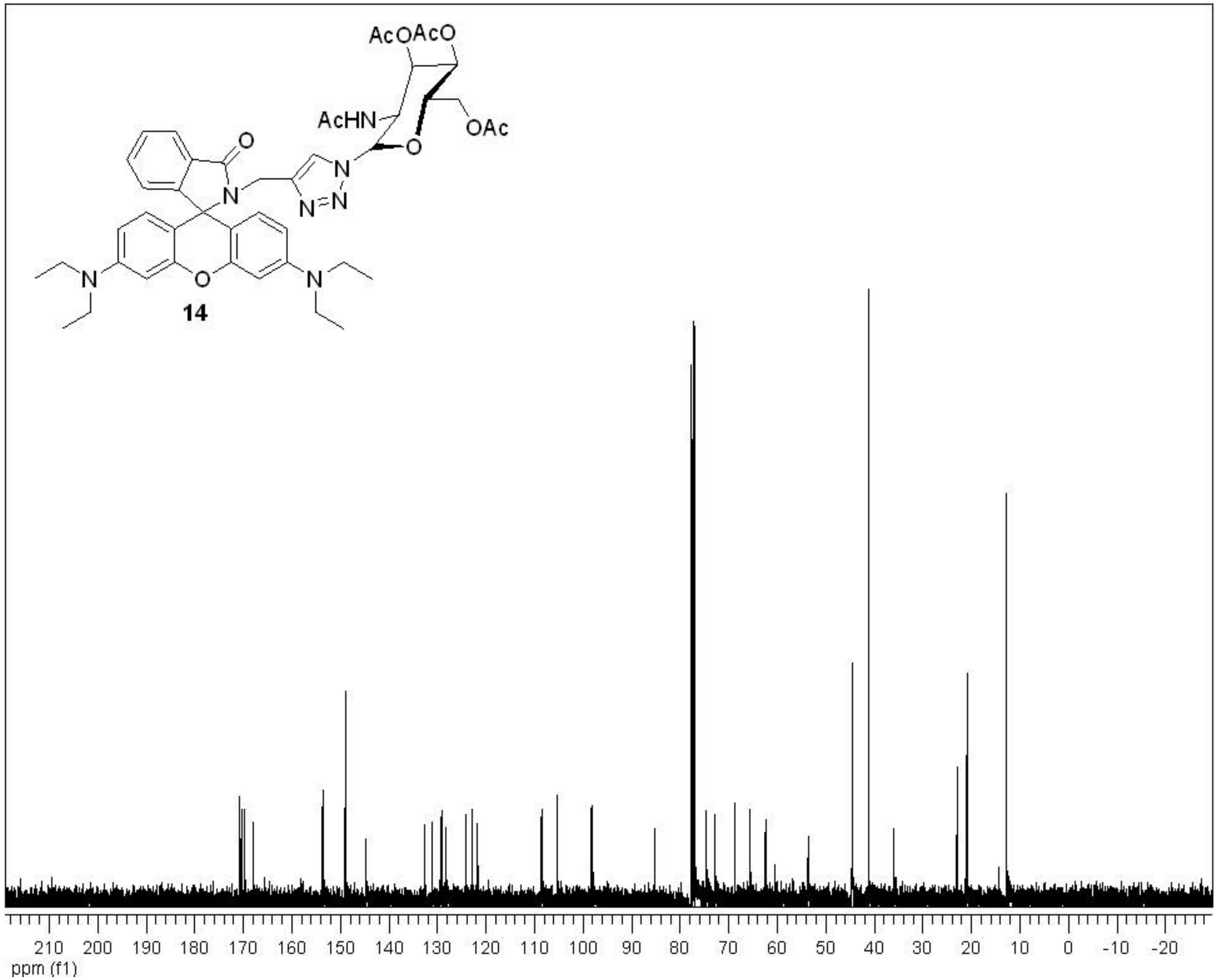

AcOAcO
OAc
AcHN
N
N=N
O
N
O
N
N
14
210 200 190 180 170 160 150 140 130 120 110 100 90 80 70 60 50 40 30 20 10 0 -10 -20
ppm (f1)

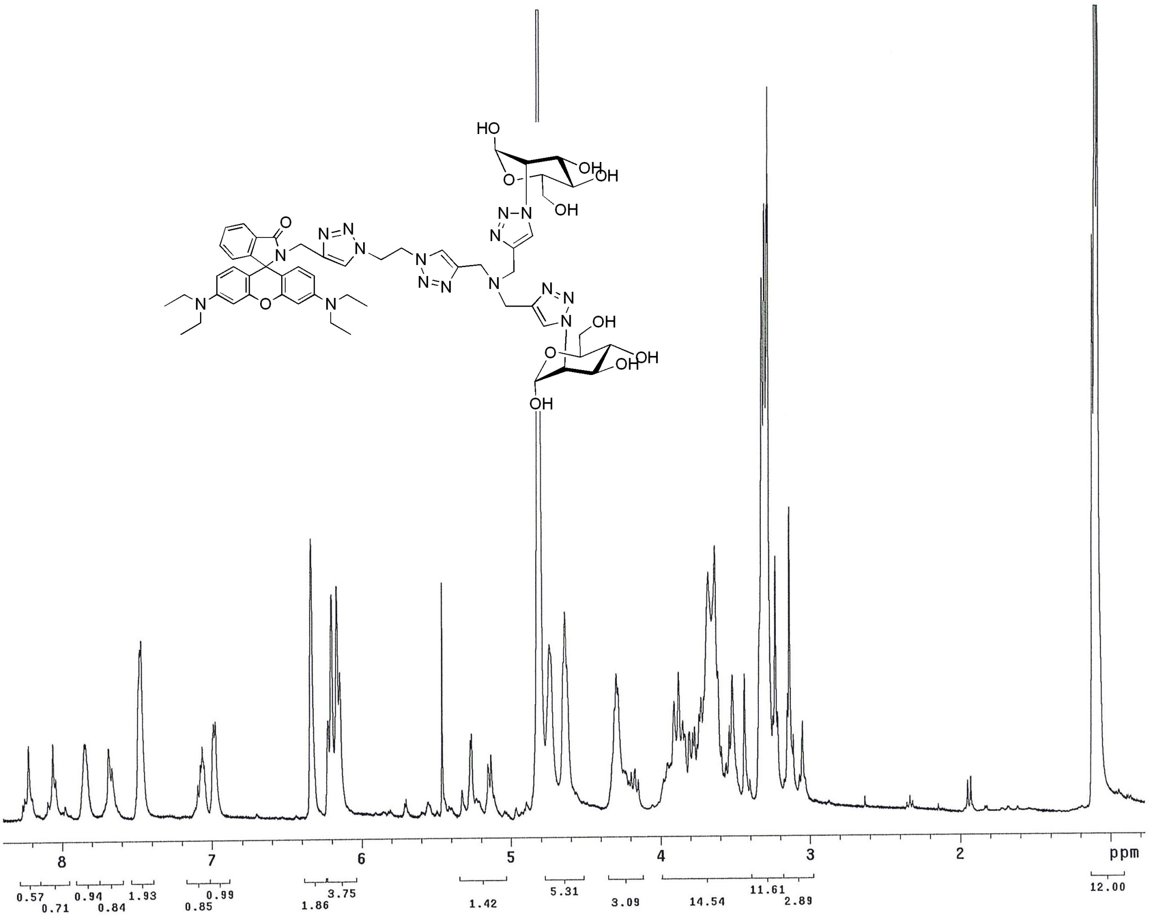
8
7
6
5
4
3
2
ppm
0.57
0.71
0.94
0.84
1.93
0.85
0.99
1.86
3.75
1.42
5.31
3.09
14.54
11.61
2.89
12.00

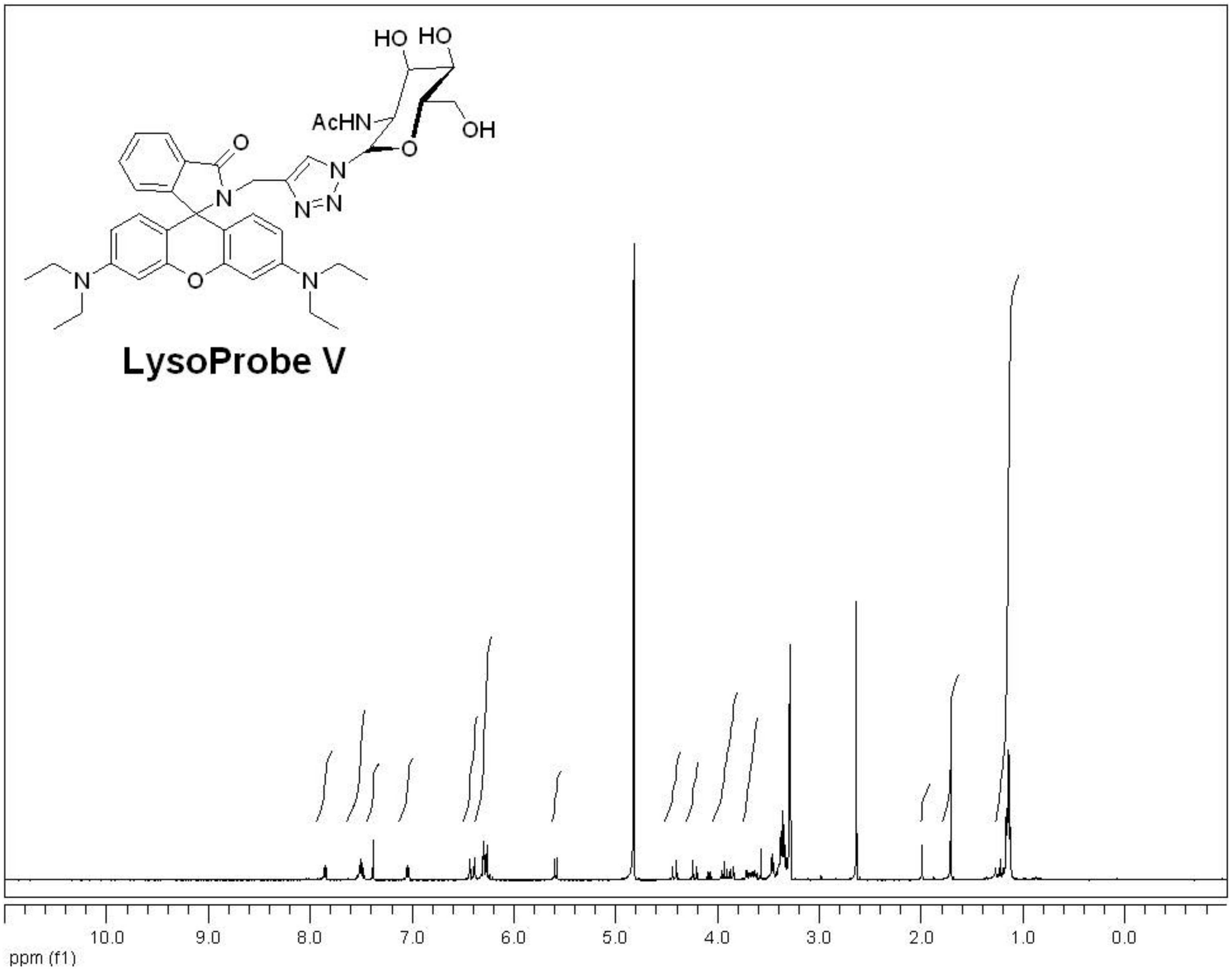
HO HO
AcHN
OH
LysoProbe V
10.0
9.0
8.0
7.0
6.0
5.0
4.0
3.0
2.0
1.0
0.0
ppm (f1)

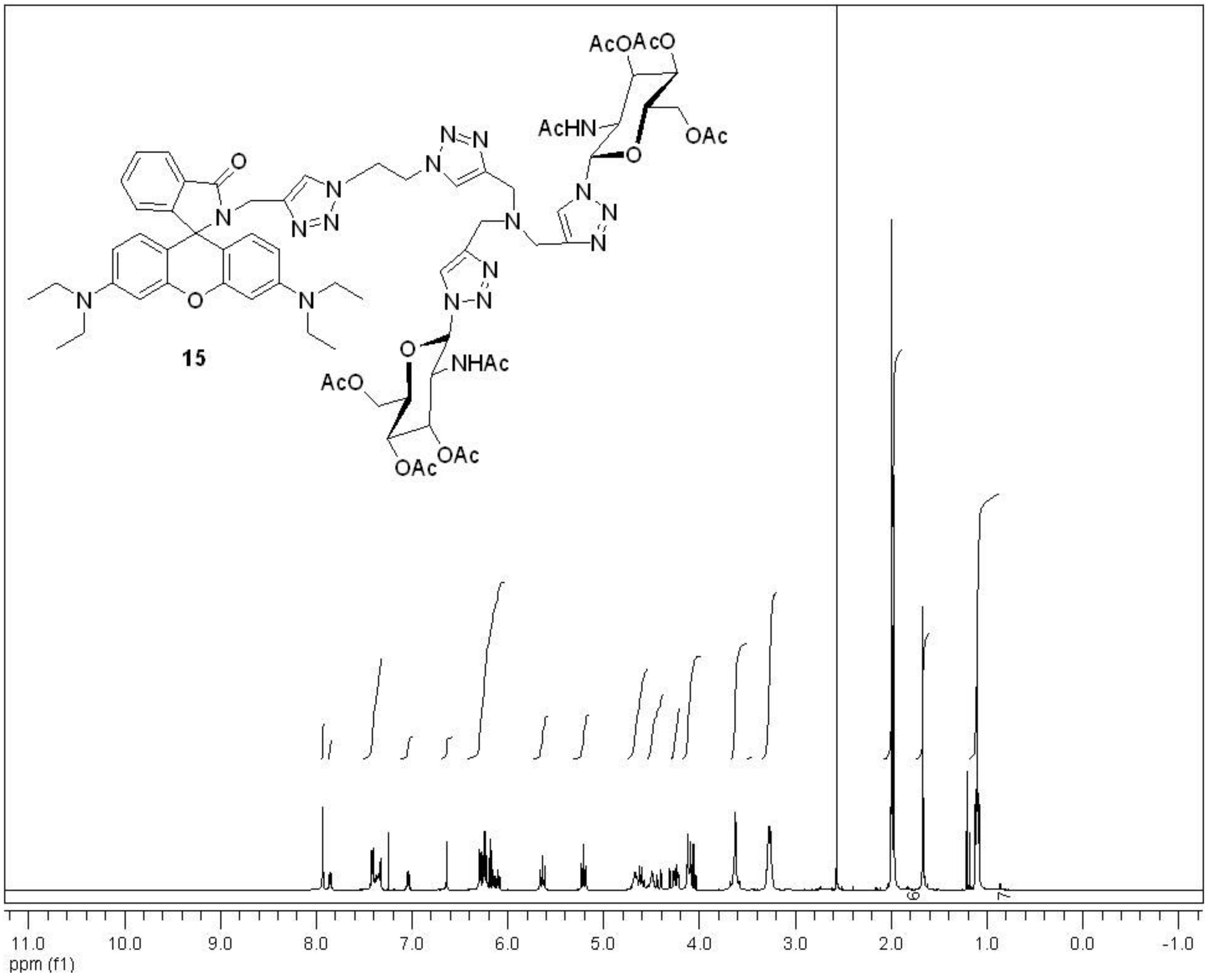
AcOAcO
AcHN
OAc
O
N
15
AcO
NHAc
OAc
OAc
11.0
10.0
9.0
8.0
7.0
6.0
5.0
4.0
3.0
2.0
1.0
0.0
-1.0
ppm (f1)

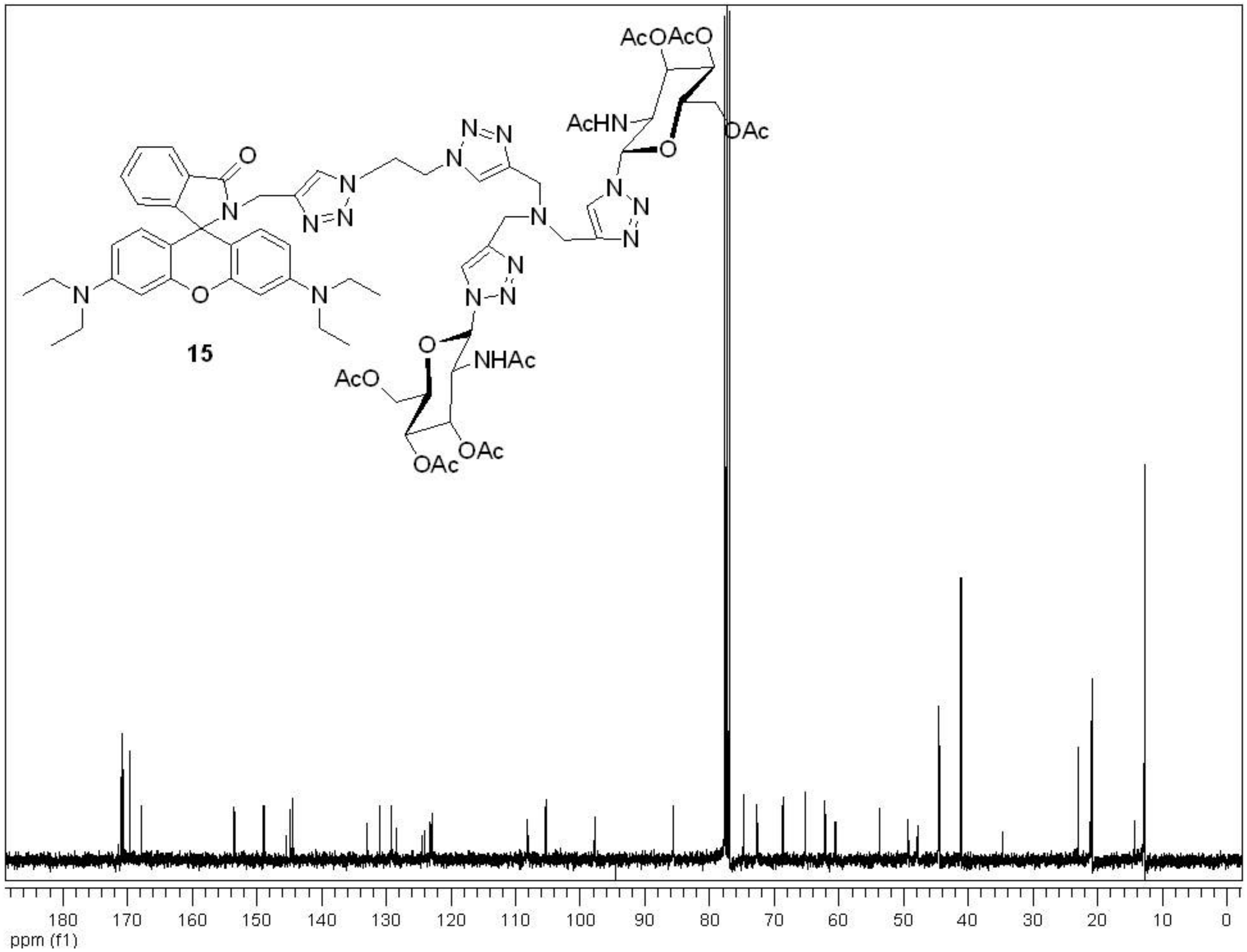

15
AcOAcO
AcHN
OAc
NHAc
AcO
OAc
OAc
180 170 160 150 140 130 120 110 100 90 80 70 60 50 40 30 20 10 0
ppm (f1)

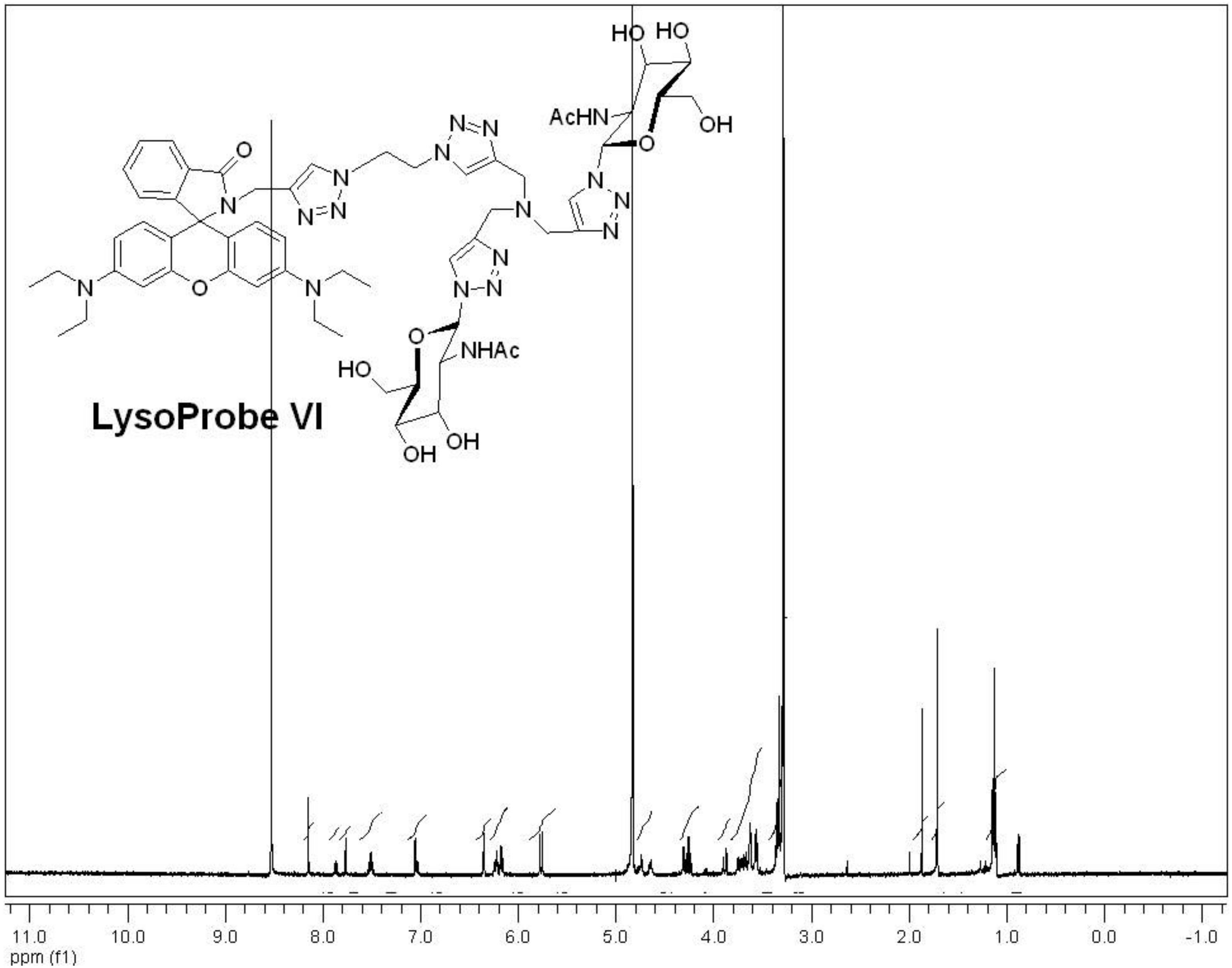
LysoProbe VI
AcHN
NHAc
HO
OH
11.0
10.0
9.0
8.0
7.0
6.0
5.0
4.0
3.0
2.0
1.0
0.0
-1.0
ppm (f1)

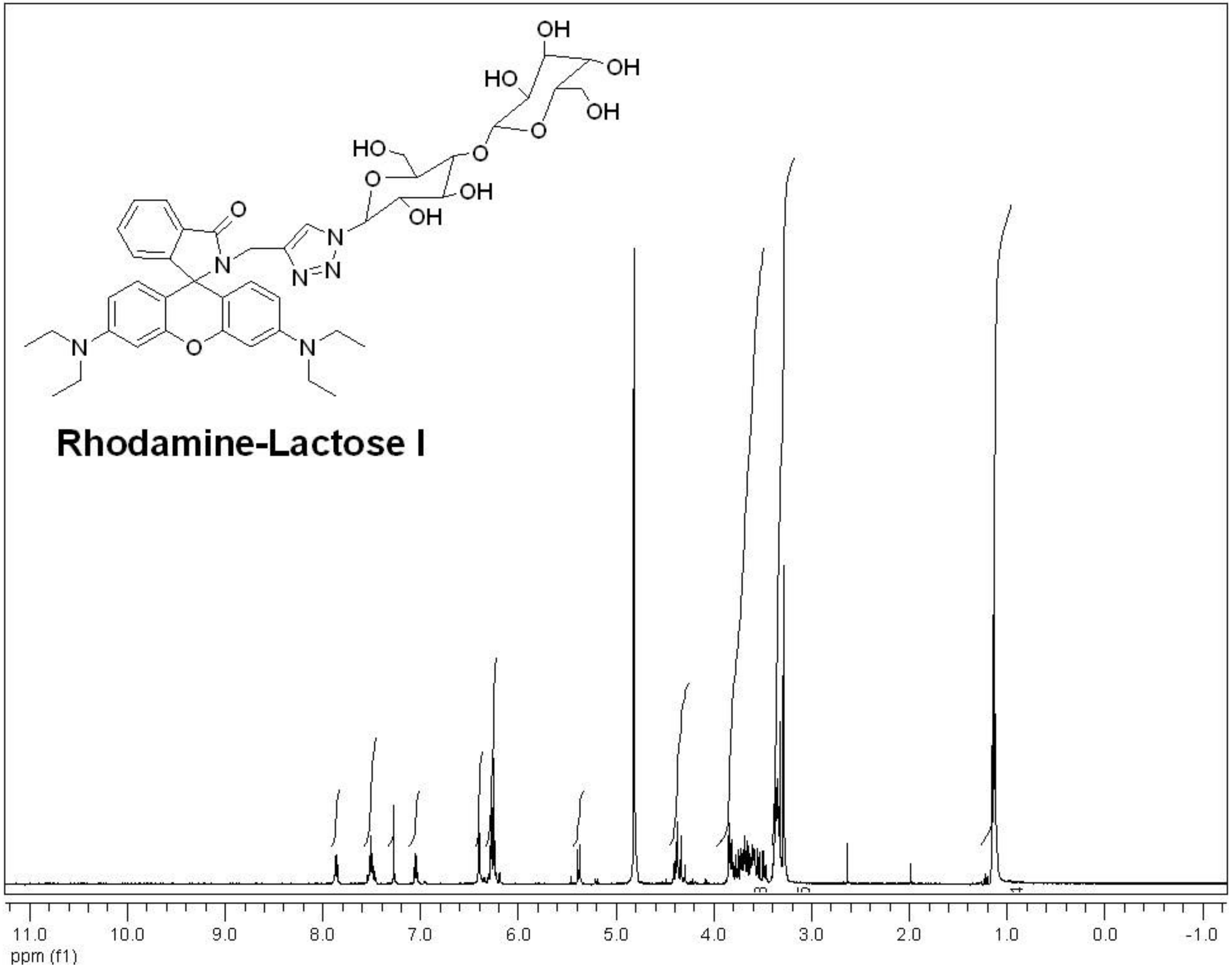
Rhodamine-Lactose I
ppm (f1)
11.0
10.0
9.0
8.0
7.0
6.0
5.0
4.0
3.0
2.0
1.0
0.0
-1.0

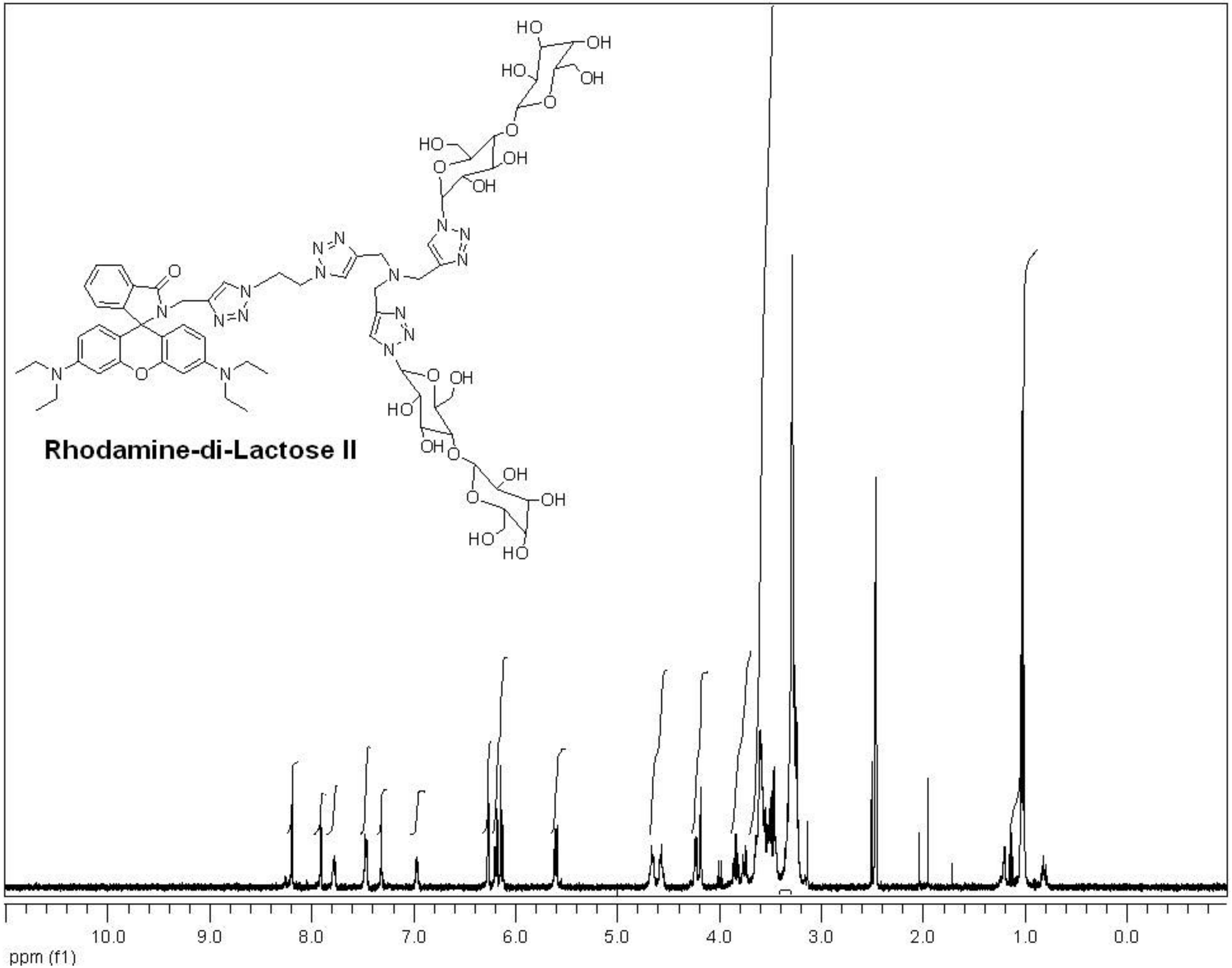
Rhodamine-di-Lactose II
10.0
9.0
8.0
7.0
6.0
5.0
4.0
3.0
2.0
1.0
0.0
ppm (f1)
